# Supplementary material for: Use of Secondary Metabolites Profiling and Antioxidant Activity to Unravel the Differences between Two Species of Nettle
Source: Plants (Basel). 2023 Sep 11;12(18):3233. doi: 10.3390/plants12183233 (PMC10535656; doi:10.3390/plants12183233)
Supplement: Supplementary file 1 [file plants-12-03233-s001.zip › plants-2585213-supplementary.pdf]

Varno Supplementary Material

Supplenmentary material Table S1. Factor Loadings obtained from PCA with varimax rotation analysis based on data from TLC fingerprints of separated polyphenols on Silicagel and RP-18 chromatographic plates using detection on UV254nm and UV366 nm (before and after pulverization of chromatographic plate). (The highest contribution for values >0.9)

Variable/ Rf	Factor Loadings (Varimax normalized, data from silicagel plates) Extraction:Principal components (Marked loadings are >.70)			
	Factor1	Factor2	Factor3	Factor4
0.000	0.78	0.14	0.18	0.59
0.002	0.57	-0.04	0.78	0.27
0.004	-0.33	-0.12	0.77	-0.54
0.005	-0.26	0.01	0.60	-0.76
0.007	0.31	0.22	0.30	-0.88
0.009	0.30	0.64	0.11	-0.69
0.011	-0.22	0.85	-0.49	-0.05
0.012	-0.88	0.26	-0.39	-0.06
0.014	-0.44	0.39	0.28	0.76
0.016	-0.77	0.47	0.22	0.36
0.018	-0.27	-0.49	-0.54	-0.63
0.020	0.83	-0.21	-0.24	-0.45
0.021	0.96	0.03	-0.11	-0.26
0.023	0.93	0.33	-0.05	-0.16
0.025	0.85	0.38	-0.17	0.33
0.027	-0.41	0.64	-0.47	0.44
0.028	-0.80	-0.08	-0.57	0.16
0.030	0.43	0.25	-0.83	0.26
0.032	-0.39	0.31	-0.80	-0.34
0.034	-0.00	-0.70	-0.38	-0.61
0.035	0.94	0.23	-0.26	0.06
0.037	0.85	0.28	-0.36	0.28
0.039	0.75	0.31	-0.36	0.45
0.041	0.74	0.21	-0.44	0.47
0.043	0.86	0.19	-0.35	0.31
0.044	0.94	0.00	-0.28	0.20
0.046	0.90	-0.06	-0.34	0.28
0.048	0.89	-0.05	-0.34	0.29
0.050	0.91	-0.05	-0.28	0.29
0.051	0.91	-0.07	-0.23	0.35
0.053	0.93	0.02	-0.18	0.31

0.055	0.95	0.10	-0.15	0.27
0.057	0.96	0.17	-0.12	0.20
0.059	0.96	0.22	-0.08	0.15
0.060	0.96	0.25	-0.07	0.13
0.062	0.97	0.21	-0.10	0.11
0.064	0.97	0.18	-0.15	0.09
0.066	0.98	0.11	-0.18	0.03
0.067	0.98	0.08	-0.16	-0.07
0.069	0.98	0.01	-0.15	-0.12
0.071	0.97	-0.08	-0.19	-0.09
0.073	0.96	-0.12	-0.24	-0.03
0.074	0.95	-0.10	-0.31	0.02
0.076	0.93	-0.10	-0.35	0.02
0.078	0.92	-0.13	-0.37	0.02
0.080	0.94	-0.05	-0.34	-0.04
0.082	0.94	-0.04	-0.33	-0.01
0.083	0.93	0.02	-0.36	0.04
0.085	0.93	0.03	-0.36	0.06
0.087	0.93	0.02	-0.37	0.03
0.089	0.91	0.02	-0.40	0.02
0.090	0.91	0.00	-0.42	-0.00
0.092	0.89	0.01	-0.45	-0.03
0.094	0.86	0.08	-0.50	-0.01
0.096	0.87	0.08	-0.49	0.02
0.098	0.87	0.08	-0.48	-0.01
0.099	0.87	0.07	-0.49	-0.03
0.101	0.85	0.06	-0.52	-0.04
0.103	0.84	0.05	-0.53	-0.03
0.105	0.86	0.06	-0.51	-0.03
0.106	0.87	0.05	-0.47	-0.09
0.108	0.87	-0.01	-0.47	-0.14
0.110	0.87	-0.02	-0.46	-0.17
0.112	0.88	-0.03	-0.45	-0.18
0.113	0.89	-0.01	-0.41	-0.19
0.115	0.92	-0.00	-0.35	-0.19
0.117	0.93	-0.01	-0.34	-0.12
0.119	0.94	-0.02	-0.32	-0.11
0.121	0.94	-0.02	-0.33	-0.11
0.122	0.91	-0.08	-0.39	-0.09
0.124	0.92	-0.14	-0.35	-0.12
0.126	0.96	-0.09	-0.26	-0.09

0.128	0.96	-0.06	-0.25	-0.08
0.129	0.96	-0.06	-0.25	-0.09
0.131	0.97	-0.09	-0.21	-0.08
0.133	0.97	-0.11	-0.23	-0.04
0.135	0.96	-0.13	-0.26	-0.05
0.137	0.96	-0.12	-0.23	-0.08
0.138	0.97	-0.10	-0.22	-0.06
0.140	0.97	-0.07	-0.20	-0.12
0.142	0.96	-0.08	-0.19	-0.17
0.144	0.97	-0.06	-0.17	-0.16
0.145	0.98	-0.04	-0.13	-0.15
0.147	0.98	-0.06	-0.10	-0.14
0.149	0.98	-0.12	-0.09	-0.14
0.151	0.98	-0.09	-0.07	-0.17
0.152	0.98	-0.12	-0.11	-0.12
0.154	0.98	-0.08	-0.06	-0.16
0.156	0.98	-0.06	-0.02	-0.20
0.158	0.98	-0.07	-0.01	-0.18
0.160	0.99	-0.10	-0.01	-0.11
0.161	1.00	-0.05	0.01	-0.08
0.163	1.00	0.01	0.02	-0.10
0.165	0.99	0.04	0.04	-0.13
0.167	0.99	0.03	0.07	-0.14
0.168	0.98	0.03	0.11	-0.18
0.170	0.98	0.01	0.11	-0.19
0.172	0.98	0.02	0.08	-0.16
0.174	0.99	0.03	0.08	-0.13
0.176	0.98	0.07	0.08	-0.14
0.177	0.97	0.09	0.12	-0.17
0.179	0.96	0.09	0.17	-0.19
0.181	0.96	0.12	0.18	-0.20
0.183	0.96	0.12	0.15	-0.21
0.184	0.96	0.11	0.17	-0.21
0.186	0.96	0.09	0.20	-0.19
0.188	0.95	0.07	0.19	-0.22
0.190	0.94	0.06	0.21	-0.25
0.191	0.93	0.09	0.26	-0.24
0.193	0.94	0.06	0.27	-0.22
0.195	0.96	0.07	0.22	-0.18
0.197	0.96	0.05	0.21	-0.17
0.199	0.97	0.05	0.19	-0.14

0.200	0.98	0.07	0.16	-0.12
0.202	0.98	0.08	0.16	-0.10
0.204	0.98	0.10	0.15	-0.05
0.206	0.98	0.10	0.15	-0.04
0.207	0.98	0.08	0.15	-0.04
0.209	0.97	0.05	0.24	-0.06
0.211	0.97	0.05	0.22	-0.07
0.213	0.98	0.09	0.18	-0.05
0.215	0.98	0.11	0.14	-0.01
0.216	0.98	0.08	0.15	0.04
0.218	0.98	0.08	0.15	0.07
0.220	0.98	0.12	0.15	0.05
0.222	0.98	0.11	0.15	0.04
0.223	0.98	0.09	0.15	0.04
0.225	0.99	0.07	0.15	0.02
0.227	0.99	0.04	0.15	0.05
0.229	0.99	0.01	0.14	0.09
0.230	0.99	-0.01	0.12	0.08
0.232	0.99	0.00	0.14	0.07
0.234	0.99	0.02	0.15	0.07
0.236	0.99	0.03	0.10	0.09
0.238	0.98	-0.02	0.14	0.12
0.239	0.99	-0.04	0.11	0.12
0.241	0.98	-0.06	0.12	0.13
0.243	0.98	-0.07	0.13	0.15
0.245	0.97	-0.12	0.14	0.18
0.246	0.95	-0.18	0.14	0.20
0.248	0.95	-0.21	0.14	0.20
0.250	0.94	-0.22	0.15	0.20
0.252	0.93	-0.27	0.17	0.17
0.254	0.92	-0.31	0.17	0.18
0.255	0.89	-0.37	0.17	0.21
0.257	0.87	-0.39	0.19	0.21
0.259	0.87	-0.41	0.19	0.20
0.261	0.85	-0.43	0.22	0.20
0.262	0.83	-0.43	0.24	0.25
0.264	0.81	-0.44	0.24	0.30
0.266	0.79	-0.44	0.30	0.29
0.268	0.78	-0.46	0.31	0.29
0.270	0.77	-0.48	0.32	0.27
0.271	0.78	-0.47	0.31	0.26

0.273	0.79	-0.45	0.33	0.26
0.275	0.77	-0.45	0.37	0.25
0.277	0.75	-0.45	0.41	0.25
0.278	0.76	-0.43	0.43	0.22
0.280	0.78	-0.42	0.44	0.18
0.282	0.77	-0.41	0.45	0.20
0.284	0.80	-0.37	0.42	0.23
0.285	0.85	-0.31	0.36	0.23
0.287	0.86	-0.27	0.36	0.24
0.289	0.84	-0.27	0.35	0.31
0.291	0.85	-0.20	0.21	0.44
0.293	0.89	-0.06	0.07	0.45
0.294	0.88	0.08	-0.05	0.46
0.296	0.87	0.13	-0.03	0.47
0.298	0.83	0.16	-0.09	0.53
0.300	0.79	0.15	-0.16	0.57
0.301	0.80	0.12	-0.17	0.56
0.303	0.82	0.13	-0.12	0.55
0.305	0.83	0.16	-0.09	0.52
0.307	0.82	0.13	-0.11	0.55
0.309	0.85	0.11	-0.10	0.50
0.310	0.92	0.13	-0.05	0.36
0.312	0.91	0.15	-0.05	0.38
0.314	0.89	0.12	-0.05	0.44
0.316	0.91	0.07	-0.01	0.42
0.317	0.93	0.04	0.02	0.37
0.319	0.92	0.05	0.03	0.38
0.321	0.93	0.08	0.05	0.37
0.323	0.93	0.10	0.12	0.33
0.324	0.93	0.14	0.14	0.32
0.326	0.93	0.16	0.15	0.31
0.328	0.94	0.10	0.19	0.28
0.330	0.95	0.06	0.21	0.23
0.332	0.96	0.04	0.23	0.17
0.333	0.96	0.05	0.24	0.16
0.335	0.95	0.07	0.26	0.16
0.337	0.93	0.01	0.37	0.08
0.339	0.94	-0.00	0.34	0.02
0.340	0.95	-0.03	0.32	-0.07
0.342	0.94	-0.03	0.30	-0.12
0.344	0.95	0.01	0.31	-0.09

0.346	0.95	0.05	0.31	-0.06
0.348	0.95	0.04	0.28	-0.08
0.349	0.96	0.02	0.27	-0.11
0.351	0.96	0.06	0.27	-0.02
0.353	0.96	0.09	0.26	-0.06
0.355	0.96	0.12	0.23	-0.12
0.356	0.96	0.14	0.19	-0.11
0.358	0.97	0.16	0.19	-0.06
0.360	0.97	0.18	0.18	-0.01
0.362	0.97	0.14	0.20	-0.02
0.363	0.97	0.11	0.21	-0.07
0.365	0.96	0.11	0.25	-0.09
0.367	0.97	0.13	0.18	-0.09
0.369	0.98	0.14	0.14	-0.07
0.371	0.97	0.15	0.16	-0.07
0.372	0.96	0.15	0.22	-0.11
0.374	0.95	0.18	0.22	-0.16
0.376	0.94	0.17	0.21	-0.19
0.378	0.95	0.16	0.20	-0.20
0.379	0.95	0.14	0.21	-0.21
0.381	0.94	0.22	0.22	-0.14
0.383	0.94	0.21	0.24	-0.12
0.385	0.94	0.18	0.21	-0.21
0.387	0.90	0.21	0.20	-0.33
0.388	0.90	0.19	0.24	-0.32
0.390	0.93	0.10	0.27	-0.25
0.392	0.93	0.12	0.26	-0.23
0.394	0.91	0.16	0.26	-0.28
0.395	0.89	0.18	0.28	-0.29
0.397	0.86	0.16	0.34	-0.34
0.399	0.86	0.16	0.33	-0.36
0.401	0.89	0.20	0.27	-0.29
0.402	0.88	0.20	0.24	-0.36
0.404	0.86	0.15	0.27	-0.41
0.406	0.85	0.19	0.32	-0.38
0.408	0.87	0.19	0.22	-0.41
0.410	0.85	0.21	0.22	-0.43
0.411	0.83	0.19	0.24	-0.46
0.413	0.82	0.18	0.27	-0.47
0.415	0.81	0.19	0.27	-0.49
0.417	0.80	0.19	0.28	-0.50

0.418	0.80	0.18	0.28	-0.50
0.420	0.79	0.19	0.27	-0.52
0.422	0.77	0.13	0.29	-0.54
0.424	0.77	0.12	0.31	-0.55
0.426	0.76	0.11	0.31	-0.57
0.427	0.74	0.11	0.32	-0.58
0.429	0.74	0.11	0.32	-0.58
0.431	0.73	0.11	0.34	-0.58
0.433	0.75	0.10	0.34	-0.56
0.434	0.74	0.16	0.34	-0.56
0.436	0.71	0.14	0.35	-0.59
0.438	0.71	0.16	0.34	-0.59
0.440	0.72	0.18	0.32	-0.59
0.441	0.72	0.19	0.32	-0.59
0.443	0.71	0.18	0.35	-0.59
0.445	0.70	0.16	0.38	-0.59
0.447	0.69	0.17	0.40	-0.58
0.449	0.70	0.16	0.40	-0.56
0.450	0.70	0.17	0.41	-0.56
0.452	0.73	0.16	0.39	-0.54
0.454	0.74	0.13	0.37	-0.55
0.456	0.76	0.10	0.32	-0.56
0.457	0.80	0.06	0.23	-0.55
0.459	0.84	0.05	0.08	-0.53
0.461	0.85	0.09	-0.15	-0.50
0.463	0.84	0.02	-0.29	-0.45
0.465	0.80	0.03	-0.42	-0.43
0.466	0.75	0.01	-0.52	-0.40
0.468	0.68	0.01	-0.65	-0.34
0.470	0.65	0.02	-0.70	-0.29
0.472	0.64	0.05	-0.72	-0.26
0.473	0.62	0.10	-0.75	-0.22
0.475	0.63	0.09	-0.75	-0.18
0.477	0.65	0.10	-0.73	-0.18
0.479	0.61	0.11	-0.77	-0.18
0.480	0.59	0.11	-0.78	-0.20
0.482	0.61	0.12	-0.76	-0.20
0.484	0.63	0.12	-0.74	-0.20
0.486	0.63	0.14	-0.74	-0.19
0.488	0.59	0.16	-0.77	-0.18
0.489	0.58	0.21	-0.78	-0.14

0.491	0.60	0.24	-0.75	-0.15
0.493	0.67	0.33	-0.64	-0.17
0.495	0.67	0.38	-0.62	-0.13
0.496	0.67	0.50	-0.54	-0.04
0.498	0.65	0.65	-0.39	-0.02
0.500	0.56	0.82	-0.12	-0.02
0.502	0.44	0.88	0.14	0.06
0.504	0.39	0.87	0.29	0.05
0.505	0.40	0.88	0.26	-0.02
0.507	0.50	0.83	0.22	-0.04
0.509	0.46	0.85	0.25	-0.08
0.511	0.43	0.84	0.31	-0.06
0.512	0.51	0.78	0.36	-0.00
0.514	0.51	0.75	0.42	-0.03
0.516	0.54	0.70	0.43	-0.18
0.518	0.56	0.70	0.39	-0.20
0.520	0.61	0.69	0.37	-0.11
0.521	0.56	0.73	0.40	0.00
0.523	0.58	0.70	0.42	-0.04
0.525	0.63	0.66	0.39	0.09
0.527	0.66	0.60	0.34	0.31
0.528	0.71	0.52	0.31	0.36
0.530	0.78	0.45	0.31	0.29
0.532	0.80	0.43	0.21	0.36
0.534	0.75	0.42	0.08	0.50
0.535	0.77	0.42	0.08	0.48
0.537	0.81	0.44	0.05	0.38
0.539	0.84	0.46	0.07	0.27
0.541	0.83	0.50	0.17	0.19
0.543	0.78	0.57	0.25	0.12
0.544	0.73	0.62	0.29	0.04
0.546	0.72	0.62	0.30	0.06
0.548	0.78	0.53	0.32	0.12
0.550	0.75	0.56	0.34	-0.07
0.551	0.66	0.60	0.45	-0.09
0.553	0.53	0.67	0.50	-0.16
0.555	0.49	0.70	0.46	-0.25
0.557	0.54	0.68	0.46	-0.15
0.559	0.52	0.64	0.55	-0.09
0.560	0.50	0.67	0.54	-0.14
0.562	0.47	0.70	0.47	-0.28

0.564	0.45	0.73	0.45	-0.26
0.566	0.48	0.71	0.49	-0.15
0.567	0.45	0.68	0.58	-0.08
0.569	0.42	0.68	0.60	-0.05
0.571	0.41	0.68	0.60	-0.11
0.573	0.48	0.67	0.56	-0.09
0.574	0.60	0.60	0.52	-0.07
0.576	0.67	0.56	0.49	0.03
0.578	0.72	0.57	0.39	0.05
0.580	0.69	0.57	0.43	0.15
0.582	0.68	0.55	0.43	0.21
0.583	0.72	0.53	0.40	0.23
0.585	0.75	0.53	0.33	0.21
0.587	0.74	0.58	0.24	0.25
0.589	0.72	0.57	0.25	0.30
0.590	0.65	0.62	0.36	0.26
0.592	0.66	0.59	0.44	0.16
0.594	0.53	0.69	0.42	0.26
0.596	0.50	0.69	0.43	0.30
0.598	0.53	0.67	0.47	0.24
0.599	0.43	0.70	0.50	0.28
0.601	0.34	0.57	0.62	0.41
0.603	0.38	0.55	0.64	0.38
0.605	0.59	0.74	0.30	0.14
0.606	0.56	0.76	0.32	-0.07
0.608	0.28	0.77	0.57	0.02
0.610	0.21	0.78	0.58	-0.08
0.612	0.36	0.80	0.46	-0.13
0.613	0.29	0.79	0.53	0.02
0.615	0.17	0.83	0.53	-0.01
0.617	0.11	0.91	0.34	-0.20
0.619	0.07	0.91	0.20	-0.36
0.621	0.35	0.78	0.34	-0.38
0.622	0.38	0.76	0.49	-0.18
0.624	0.39	0.76	0.44	-0.29
0.626	0.35	0.73	0.22	-0.55
0.628	0.35	0.79	0.31	-0.40
0.629	0.33	0.70	0.50	-0.40
0.631	0.37	0.60	0.54	-0.46
0.633	0.39	0.60	0.50	-0.48
0.635	0.40	0.68	0.60	-0.13

0.637	0.35	0.80	0.43	-0.23
0.638	0.20	0.81	0.33	-0.43
0.640	0.23	0.78	0.44	-0.38
0.642	0.47	0.72	0.50	-0.10
0.644	0.52	0.61	0.56	-0.22
0.645	0.41	0.61	0.56	-0.39
0.647	0.26	0.87	0.42	-0.02
0.649	0.29	0.76	0.58	-0.07
0.651	0.29	0.75	0.53	-0.28
0.652	0.27	0.76	0.40	-0.44
0.654	0.26	0.84	0.32	-0.36
0.656	0.27	0.88	0.35	-0.16
0.658	0.21	0.85	0.47	-0.05
0.660	0.01	0.85	0.51	-0.13
0.661	-0.12	0.88	0.45	-0.12
0.663	0.04	0.76	0.56	-0.32
0.665	-0.10	0.88	0.46	-0.05
0.667	-0.25	0.89	0.37	0.05
0.668	-0.13	0.92	0.32	-0.21
0.670	0.01	0.90	0.31	-0.30
0.672	-0.04	0.91	0.33	-0.24
0.674	-0.25	0.85	0.43	-0.18
0.676	-0.45	0.72	0.49	-0.21
0.677	-0.43	0.83	0.25	-0.24
0.679	-0.20	0.93	0.28	-0.07
0.681	-0.03	0.92	0.39	-0.06
0.683	-0.12	0.81	0.52	-0.25
0.684	-0.19	0.68	0.61	-0.35
0.686	-0.17	0.71	0.59	-0.33
0.688	-0.28	0.77	0.44	-0.38
0.690	-0.38	0.76	0.36	-0.38
0.691	-0.40	0.78	0.36	-0.33
0.693	-0.44	0.71	0.54	0.03
0.695	-0.44	0.71	0.55	0.01
0.697	-0.41	0.75	0.47	-0.21
0.699	-0.29	0.81	0.50	-0.10
0.700	-0.17	0.91	0.38	0.05
0.702	-0.08	0.99	0.14	-0.06
0.704	-0.11	0.99	0.03	-0.13
0.706	-0.36	0.86	0.34	-0.10
0.707	0.02	0.74	0.66	-0.10

0.709	0.01	0.81	0.50	-0.30
0.711	0.30	0.81	0.33	-0.38
0.713	0.53	0.62	0.42	-0.40
0.715	0.25	0.70	0.52	-0.43
0.716	0.17	0.81	0.38	-0.41
0.718	0.56	0.76	0.26	-0.20
0.720	0.34	0.83	0.14	-0.42
0.722	0.56	0.72	0.28	-0.29
0.723	0.71	0.58	0.31	-0.26
0.725	0.76	0.54	0.18	-0.30
0.727	0.69	0.64	0.14	-0.30
0.729	0.68	0.67	0.14	-0.24
0.730	0.71	0.63	0.15	-0.27
0.732	0.74	0.55	0.06	-0.38
0.734	0.78	0.58	0.03	-0.24
0.736	0.80	0.56	0.09	-0.21
0.738	0.85	0.46	0.19	-0.18
0.739	0.92	0.35	0.14	-0.11
0.741	0.94	0.29	-0.00	-0.20
0.743	0.94	0.28	-0.12	-0.17
0.745	0.96	0.24	-0.12	-0.05
0.746	0.97	0.23	-0.06	0.02
0.748	0.97	0.13	-0.11	-0.14
0.750	0.97	-0.01	-0.25	-0.01
0.752	0.94	-0.07	-0.28	0.17
0.754	0.96	-0.01	-0.21	0.15
0.755	0.99	0.02	-0.16	0.04
0.757	0.98	0.05	-0.21	-0.02
0.759	0.96	0.10	-0.25	-0.01
0.761	0.96	0.22	-0.17	0.03
0.762	0.95	0.28	0.01	-0.11
0.764	0.90	0.42	0.12	-0.09
0.766	0.77	0.59	0.16	-0.17
0.768	0.70	0.67	0.09	-0.24
0.770	0.68	0.71	0.20	-0.03
0.771	0.45	0.78	0.43	0.08
0.773	0.24	0.89	0.38	-0.02
0.775	0.16	0.99	-0.05	-0.02
0.777	0.16	0.98	-0.03	-0.14
0.778	0.24	0.91	0.32	0.09
0.780	0.10	0.93	0.32	0.16

0.782	-0.17	0.96	0.21	0.03
0.784	-0.39	0.84	0.27	-0.25
0.785	-0.24	0.93	0.28	-0.03
0.787	-0.11	0.94	0.18	0.26
0.789	-0.28	0.91	0.24	0.21
0.791	-0.56	0.76	0.16	0.27
0.793	-0.52	0.65	-0.15	0.53
0.794	-0.66	0.36	-0.18	0.64
0.796	-0.74	0.47	-0.03	0.48
0.798	-0.80	0.51	0.15	0.27
0.800	-0.85	0.29	0.40	0.15
0.801	-0.82	0.42	0.32	0.24
0.803	-0.82	0.45	0.05	0.34
0.805	-0.88	0.46	-0.07	0.08
0.807	-0.95	0.31	0.08	0.00
0.809	-0.95	0.32	0.04	-0.01
0.810	-0.89	0.44	-0.08	-0.08
0.812	-0.98	0.20	0.03	-0.07
0.814	-0.98	0.17	0.09	-0.05
0.816	-0.93	0.35	0.03	0.03
0.817	-0.63	0.75	-0.13	0.16
0.819	-0.62	0.73	0.27	-0.10
0.821	-0.66	0.51	0.55	-0.09
0.823	-0.90	0.22	0.39	-0.01
0.824	-0.98	-0.01	0.11	-0.18
0.826	-0.88	0.14	0.27	-0.37
0.828	-0.73	0.42	0.22	-0.49
0.830	-0.57	0.59	-0.08	-0.56
0.832	-0.45	0.70	-0.31	-0.45
0.833	-0.62	0.46	-0.21	0.60
0.835	-0.57	0.27	-0.23	0.74
0.837	-0.70	0.31	-0.09	0.64
0.839	-0.77	0.21	0.03	0.60
0.840	-0.72	0.24	0.03	0.65
0.842	-0.91	0.03	-0.09	0.40
0.844	-0.89	-0.21	-0.37	0.16
0.846	-0.59	0.31	-0.56	0.49
0.848	-0.61	-0.67	0.27	-0.31
0.849	-0.95	0.28	0.10	0.07
0.851	-0.67	0.21	-0.71	-0.02
0.853	-0.22	0.10	-0.94	-0.23

0.855	-0.37	0.65	-0.59	-0.29
0.856	-0.69	0.67	-0.27	0.07
0.858	-0.84	0.39	-0.35	0.10
0.860	-0.43	0.85	0.08	-0.29
0.862	-0.56	0.34	0.45	-0.61
0.863	-0.70	0.65	-0.18	0.24
0.865	-0.01	0.33	-0.90	0.28
0.867	0.24	0.09	-0.96	0.12
0.869	0.33	0.29	-0.86	-0.25
0.871	-0.10	0.38	-0.45	-0.80
0.872	-0.52	0.17	0.07	-0.83
0.874	-0.71	-0.49	-0.33	-0.36
0.876	-0.63	0.13	-0.24	-0.73
0.878	-0.41	0.41	-0.06	-0.81
0.879	-0.27	0.29	-0.32	-0.86
0.881	0.12	0.84	-0.27	-0.45
0.883	0.24	0.95	-0.12	-0.18
0.885	0.10	0.98	-0.17	0.07
0.887	0.16	0.78	-0.12	0.60
0.888	0.45	0.68	0.24	0.52
0.890	0.58	0.69	0.44	0.06
0.892	-0.03	0.69	0.52	0.50
0.894	0.55	0.70	0.02	0.46
0.895	0.69	0.68	0.16	-0.19
0.897	0.11	0.17	0.21	-0.96
0.899	-0.31	-0.31	-0.24	-0.87
0.901	0.24	0.69	0.31	-0.60
0.902	0.21	0.06	0.67	0.70
0.904	-0.82	0.54	-0.09	-0.15
0.906	-0.60	0.54	0.25	-0.54
0.908	-0.37	0.07	0.36	-0.85
0.910	-0.28	0.14	0.60	-0.73
0.911	-0.81	-0.03	0.25	-0.52
0.913	-0.89	0.30	-0.29	-0.19
0.915	-0.48	0.58	-0.32	-0.58
0.917	-0.13	0.17	-0.35	-0.91
0.918	-0.60	0.75	0.26	0.14
0.920	-0.75	0.55	0.34	0.15
0.922	-0.88	0.19	0.44	0.02
0.924	-0.92	-0.13	0.38	-0.06
0.926	-0.94	-0.29	0.14	-0.07

0.927	-0.96	-0.04	-0.08	-0.27
0.929	-0.54	0.58	0.43	-0.42
0.931	-0.41	0.34	0.80	-0.26
0.933	0.50	-0.54	0.48	-0.48
0.934	0.48	-0.84	0.23	-0.05
0.936	0.68	-0.71	-0.07	0.17
0.938	0.78	-0.41	-0.44	-0.18
0.940	0.76	-0.52	-0.31	-0.22
0.941	0.68	-0.70	0.12	-0.18
0.943	0.45	-0.15	0.52	0.71
0.945	-0.61	0.56	0.02	0.55
0.947	-0.75	0.65	0.04	0.14
0.949	-0.68	0.00	-0.41	0.61
0.950	-0.03	-0.30	-0.15	0.94
0.952	-0.03	-0.02	0.22	0.97
0.954	-0.16	0.19	0.36	0.90
0.956	-0.33	0.37	0.22	0.84
0.957	-0.31	0.42	0.50	0.69
0.959	-0.20	0.37	0.64	0.64
0.961	-0.51	0.30	0.80	-0.07
0.963	-0.24	0.29	0.84	-0.39
0.965	-0.13	-0.55	0.82	-0.07
0.966	-0.61	-0.38	0.61	0.34
0.968	-0.38	-0.01	0.77	0.51
0.970	0.23	0.21	0.82	0.47
0.972	-0.02	0.11	0.85	0.52
0.973	-0.70	0.47	0.42	-0.34
0.975	-0.55	0.10	0.71	0.42
0.977	-0.50	0.25	0.38	0.74
0.979	-0.31	0.46	0.39	0.73
0.980	-0.39	0.28	0.58	0.66
0.982	-0.83	-0.17	0.48	0.23
0.984	-0.88	0.08	0.46	0.07
0.986	-0.77	0.13	0.58	-0.22
0.988	-0.45	-0.13	0.72	-0.51
0.989	-0.52	0.35	0.75	-0.22
0.991	-0.89	0.20	0.38	0.17
0.993	-0.89	0.29	0.26	0.24
0.995	-0.88	0.24	0.30	0.27
0.996	-0.80	0.08	0.59	0.04
0.998	-0.90	-0.32	0.22	0.20

1.000	-0.53	0.57	0.32	0.54
0.000	-0.87	-0.39	-0.02	0.30
0.002	-0.47	-0.24	0.61	-0.59
0.004	-0.04	-0.05	0.44	-0.90
0.005	0.04	-0.01	0.55	-0.83
0.007	0.53	0.03	0.31	-0.79
0.009	0.55	0.31	0.30	-0.71
0.011	0.61	0.57	0.05	-0.54
0.012	0.71	0.64	-0.01	-0.29
0.014	0.81	0.57	-0.04	-0.12
0.016	0.87	0.41	-0.02	-0.29
0.018	0.93	0.21	0.05	-0.31
0.020	0.72	0.63	-0.17	-0.25
0.021	0.89	0.44	0.10	-0.00
0.023	0.27	0.88	-0.30	-0.26
0.025	0.55	0.09	-0.70	-0.45
0.027	0.85	-0.39	-0.30	-0.19
0.028	0.93	-0.36	-0.04	-0.04
0.030	0.96	-0.08	0.24	0.14
0.032	0.74	0.37	0.52	0.23
0.034	0.51	0.48	0.64	0.31
0.035	0.42	0.47	0.71	0.32
0.037	0.45	0.37	0.76	0.28
0.039	0.26	0.32	0.88	0.26
0.041	0.03	0.45	0.82	0.35
0.043	0.04	0.67	0.64	0.37
0.044	0.16	0.82	0.45	0.31
0.046	0.48	0.85	0.18	0.09
0.048	0.52	0.85	0.07	0.08
0.050	0.39	0.90	-0.00	0.17
0.051	0.24	0.95	0.00	0.21
0.053	0.13	0.98	0.03	0.16
0.055	0.07	0.98	0.04	0.15
0.057	0.07	0.98	0.07	0.19
0.059	0.09	0.98	0.09	0.16
0.060	0.03	0.98	0.13	0.14
0.062	0.02	0.98	0.15	0.13
0.064	0.00	0.98	0.15	0.12
0.066	-0.03	0.98	0.12	0.14
0.067	-0.05	0.98	0.09	0.16
0.069	-0.05	0.98	0.07	0.18

0.071	-0.03	0.98	0.05	0.19
0.073	-0.03	0.98	0.02	0.21
0.074	-0.08	0.97	-0.04	0.24
0.076	-0.10	0.95	-0.08	0.28
0.078	-0.13	0.93	-0.13	0.32
0.080	-0.16	0.92	-0.16	0.33
0.082	-0.18	0.90	-0.17	0.35
0.083	-0.20	0.89	-0.18	0.37
0.085	-0.22	0.87	-0.19	0.40
0.087	-0.24	0.86	-0.20	0.41
0.089	-0.21	0.85	-0.19	0.45
0.090	-0.17	0.86	-0.17	0.45
0.092	-0.15	0.87	-0.18	0.44
0.094	-0.13	0.87	-0.18	0.44
0.096	-0.09	0.88	-0.16	0.43
0.098	-0.05	0.90	-0.14	0.41
0.099	-0.02	0.92	-0.12	0.37
0.101	0.01	0.93	-0.10	0.35
0.103	0.04	0.93	-0.05	0.36
0.105	0.04	0.93	-0.05	0.35
0.106	0.03	0.94	-0.03	0.33
0.108	0.04	0.95	-0.01	0.30
0.110	0.04	0.96	0.01	0.28
0.112	0.05	0.96	0.01	0.29
0.113	0.06	0.96	-0.00	0.28
0.115	0.05	0.96	-0.01	0.29
0.117	0.09	0.94	-0.01	0.33
0.119	0.08	0.94	-0.03	0.33
0.121	0.08	0.94	-0.04	0.33
0.122	0.09	0.94	-0.03	0.32
0.124	0.06	0.94	-0.02	0.34
0.126	0.06	0.93	-0.03	0.36
0.128	0.07	0.92	-0.04	0.38
0.129	0.06	0.92	-0.05	0.39
0.131	0.11	0.92	-0.05	0.37
0.133	0.12	0.92	-0.05	0.37
0.135	0.15	0.91	-0.07	0.38
0.137	0.19	0.89	-0.08	0.40
0.138	0.23	0.88	-0.10	0.40
0.140	0.27	0.88	-0.10	0.38
0.142	0.30	0.87	-0.09	0.37

0.144	0.33	0.87	-0.10	0.36
0.145	0.38	0.86	-0.06	0.35
0.147	0.41	0.85	-0.07	0.33
0.149	0.46	0.83	-0.05	0.32
0.151	0.48	0.82	-0.04	0.32
0.152	0.50	0.80	-0.05	0.32
0.154	0.50	0.80	-0.05	0.33
0.156	0.53	0.78	-0.05	0.34
0.158	0.54	0.76	-0.06	0.36
0.160	0.55	0.76	-0.07	0.33
0.161	0.57	0.76	-0.05	0.32
0.163	0.58	0.75	-0.03	0.32
0.165	0.59	0.74	-0.01	0.32
0.167	0.62	0.73	0.01	0.29
0.168	0.64	0.73	0.01	0.25
0.170	0.66	0.72	0.06	0.18
0.172	0.67	0.72	0.11	0.15
0.174	0.72	0.68	0.11	0.10
0.176	0.74	0.66	0.13	0.07
0.177	0.76	0.63	0.16	0.04
0.179	0.77	0.61	0.20	0.00
0.181	0.78	0.58	0.24	-0.04
0.183	0.80	0.54	0.26	-0.07
0.184	0.82	0.49	0.26	-0.11
0.186	0.85	0.45	0.25	-0.12
0.188	0.82	0.42	0.35	-0.16
0.190	0.80	0.41	0.40	-0.18
0.191	0.76	0.41	0.45	-0.24
0.193	0.71	0.38	0.50	-0.33
0.195	0.64	0.35	0.56	-0.38
0.197	0.56	0.33	0.64	-0.40
0.199	0.49	0.32	0.68	-0.43
0.200	0.44	0.32	0.69	-0.47
0.202	0.36	0.31	0.75	-0.46
0.204	0.32	0.34	0.76	-0.44
0.206	0.29	0.37	0.78	-0.41
0.207	0.24	0.40	0.79	-0.40
0.209	0.20	0.41	0.80	-0.39
0.211	0.14	0.47	0.79	-0.37
0.213	0.12	0.55	0.77	-0.31
0.215	0.09	0.61	0.74	-0.27

0.216	-0.05	0.72	0.65	-0.23
0.218	-0.08	0.79	0.59	-0.15
0.220	-0.12	0.87	0.47	-0.06
0.222	-0.14	0.93	0.35	-0.01
0.223	-0.13	0.95	0.26	0.05
0.225	-0.10	0.97	0.19	0.12
0.227	-0.07	0.97	0.14	0.20
0.229	-0.06	0.96	0.10	0.24
0.230	-0.01	0.96	0.01	0.27
0.232	0.01	0.95	-0.01	0.30
0.234	-0.00	0.94	-0.04	0.34
0.236	-0.01	0.92	-0.06	0.38
0.238	-0.01	0.92	-0.06	0.38
0.239	-0.01	0.91	-0.07	0.40
0.241	0.01	0.90	-0.08	0.42
0.243	0.04	0.90	-0.09	0.43
0.245	0.02	0.91	-0.10	0.41
0.246	0.03	0.91	-0.10	0.41
0.248	0.02	0.91	-0.11	0.39
0.250	0.00	0.93	-0.12	0.35
0.252	-0.01	0.94	-0.13	0.33
0.254	-0.01	0.94	-0.13	0.30
0.255	-0.03	0.96	-0.14	0.25
0.257	-0.04	0.97	-0.15	0.21
0.259	-0.05	0.98	-0.13	0.15
0.261	-0.05	0.98	-0.13	0.14
0.262	-0.05	0.98	-0.14	0.10
0.264	-0.05	0.98	-0.15	0.08
0.266	-0.06	0.98	-0.16	0.06
0.268	-0.06	0.98	-0.16	0.05
0.270	-0.06	0.99	-0.15	0.05
0.271	-0.06	0.99	-0.13	0.03
0.273	-0.07	0.99	-0.13	0.04
0.275	-0.06	0.99	-0.12	0.02
0.277	-0.06	0.99	-0.11	0.02
0.278	-0.08	0.99	-0.10	0.03
0.280	-0.10	0.99	-0.09	0.04
0.282	-0.11	0.99	-0.07	0.04
0.284	-0.12	0.99	-0.05	0.03
0.285	-0.12	0.99	-0.04	0.03
0.287	-0.12	0.99	0.02	-0.02

0.289	-0.14	0.99	0.04	-0.05
0.291	-0.16	0.98	0.10	-0.09
0.293	-0.14	0.97	0.17	-0.12
0.294	-0.13	0.95	0.23	-0.15
0.296	-0.12	0.93	0.30	-0.18
0.298	-0.11	0.90	0.38	-0.19
0.300	-0.09	0.86	0.45	-0.22
0.301	-0.13	0.80	0.53	-0.26
0.303	-0.16	0.75	0.59	-0.26
0.305	-0.32	0.62	0.66	-0.27
0.307	-0.59	0.41	0.64	-0.27
0.309	-0.80	0.18	0.53	-0.21
0.310	-0.90	0.01	0.41	-0.15
0.312	-0.93	-0.08	0.34	-0.11
0.314	-0.94	-0.10	0.31	-0.10
0.316	-0.93	-0.04	0.35	-0.10
0.317	-0.89	0.03	0.45	-0.12
0.319	-0.73	0.18	0.64	-0.15
0.321	-0.38	0.40	0.81	-0.18
0.323	-0.05	0.51	0.84	-0.17
0.324	0.09	0.53	0.83	-0.16
0.326	0.07	0.48	0.86	-0.15
0.328	0.01	0.44	0.88	-0.16
0.330	0.06	0.42	0.89	-0.15
0.332	0.06	0.41	0.89	-0.19
0.333	0.06	0.36	0.91	-0.21
0.335	0.07	0.34	0.92	-0.20
0.337	0.08	0.30	0.93	-0.18
0.339	0.10	0.30	0.94	-0.15
0.340	0.15	0.23	0.95	-0.14
0.342	0.17	0.17	0.96	-0.15
0.344	0.21	0.20	0.95	-0.09
0.346	0.21	0.21	0.95	-0.05
0.348	0.22	0.23	0.95	0.00
0.349	0.21	0.23	0.95	0.04
0.351	0.17	0.24	0.95	0.06
0.353	0.11	0.32	0.93	0.12
0.355	0.02	0.41	0.89	0.19
0.356	-0.09	0.50	0.83	0.24
0.358	-0.36	0.48	0.79	0.07
0.360	-0.42	0.50	0.75	0.08

0.362	-0.48	0.53	0.69	0.10
0.363	-0.56	0.50	0.64	0.13
0.365	-0.62	0.47	0.60	0.15
0.367	-0.69	0.39	0.59	0.15
0.369	-0.75	0.31	0.56	0.14
0.371	-0.78	0.25	0.56	0.13
0.372	-0.79	0.26	0.51	0.21
0.374	-0.79	0.22	0.52	0.24
0.376	-0.80	0.20	0.49	0.29
0.378	-0.81	0.19	0.45	0.34
0.379	-0.81	0.23	0.38	0.38
0.381	-0.83	0.24	0.31	0.40
0.383	-0.84	0.24	0.26	0.41
0.385	-0.84	0.25	0.24	0.43
0.387	-0.81	0.24	0.16	0.51
0.388	-0.82	0.24	0.16	0.49
0.390	-0.83	0.22	0.16	0.48
0.392	-0.84	0.20	0.13	0.49
0.394	-0.86	0.20	0.10	0.47
0.395	-0.88	0.16	0.09	0.44
0.397	-0.90	0.11	0.08	0.42
0.399	-0.90	0.09	0.07	0.42
0.401	-0.91	0.08	0.04	0.41
0.402	-0.92	0.04	0.05	0.38
0.404	-0.94	0.02	0.07	0.33
0.406	-0.96	0.01	0.06	0.29
0.408	-0.96	-0.00	0.00	0.29
0.410	-0.96	-0.01	-0.01	0.29
0.411	-0.95	-0.03	-0.02	0.30
0.413	-0.95	-0.03	-0.02	0.31
0.415	-0.95	-0.04	-0.03	0.31
0.417	-0.95	-0.06	-0.03	0.31
0.418	-0.94	-0.09	-0.04	0.31
0.420	-0.94	-0.09	-0.05	0.31
0.422	-0.94	-0.07	-0.06	0.32
0.424	-0.94	-0.05	-0.07	0.33
0.426	-0.93	-0.06	-0.08	0.35
0.427	-0.92	-0.08	-0.09	0.37
0.429	-0.90	-0.08	-0.11	0.41
0.431	-0.89	-0.06	-0.12	0.43
0.433	-0.88	-0.02	-0.15	0.46

0.434	-0.86	-0.00	-0.17	0.49
0.436	-0.84	0.02	-0.18	0.51
0.438	-0.81	0.03	-0.19	0.55
0.440	-0.77	0.04	-0.20	0.60
0.441	-0.74	0.06	-0.22	0.63
0.443	-0.72	0.08	-0.23	0.65
0.445	-0.71	0.07	-0.22	0.67
0.447	-0.68	0.07	-0.25	0.69
0.449	-0.65	0.06	-0.28	0.70
0.450	-0.64	0.07	-0.31	0.70
0.452	-0.66	0.06	-0.32	0.68
0.454	-0.70	0.03	-0.33	0.63
0.456	-0.73	0.01	-0.34	0.59
0.457	-0.78	-0.03	-0.33	0.53
0.459	-0.82	-0.06	-0.26	0.50
0.461	-0.88	-0.07	-0.17	0.44
0.463	-0.92	-0.06	-0.07	0.37
0.465	-0.95	-0.06	0.04	0.30
0.466	-0.96	-0.07	0.15	0.24
0.468	-0.95	-0.05	0.25	0.17
0.470	-0.94	-0.03	0.31	0.14
0.472	-0.90	-0.03	0.43	0.07
0.473	-0.89	-0.03	0.46	0.06
0.475	-0.87	-0.04	0.50	0.02
0.477	-0.85	-0.04	0.53	-0.01
0.479	-0.85	-0.04	0.53	-0.03
0.480	-0.85	-0.04	0.53	-0.04
0.482	-0.84	-0.03	0.54	-0.05
0.484	-0.82	-0.01	0.57	-0.05
0.486	-0.81	0.04	0.58	-0.03
0.488	-0.79	0.08	0.61	-0.05
0.489	-0.75	0.14	0.64	-0.04
0.491	-0.71	0.21	0.68	-0.02
0.493	-0.65	0.27	0.71	-0.00
0.495	-0.58	0.32	0.75	0.01
0.496	-0.49	0.39	0.78	0.01
0.498	-0.38	0.45	0.81	0.02
0.500	-0.33	0.52	0.78	0.09
0.502	-0.26	0.58	0.77	0.11
0.504	-0.20	0.63	0.73	0.15
0.505	-0.13	0.66	0.71	0.21

0.507	-0.08	0.65	0.71	0.27
0.509	-0.09	0.62	0.70	0.34
0.511	-0.15	0.59	0.67	0.42
0.512	-0.21	0.57	0.65	0.46
0.514	-0.38	0.43	0.59	0.57
0.516	-0.53	0.29	0.56	0.57
0.518	-0.70	0.11	0.46	0.53
0.520	-0.81	0.00	0.38	0.44
0.521	-0.88	-0.09	0.28	0.37
0.523	-0.91	-0.15	0.21	0.32
0.525	-0.93	-0.19	0.19	0.26
0.527	-0.93	-0.22	0.18	0.21
0.528	-0.94	-0.25	0.16	0.15
0.530	-0.95	-0.25	0.16	0.12
0.532	-0.95	-0.26	0.16	0.08
0.534	-0.95	-0.25	0.17	0.05
0.535	-0.95	-0.23	0.20	0.03
0.537	-0.96	-0.19	0.22	0.00
0.539	-0.96	-0.15	0.25	-0.00
0.541	-0.95	-0.10	0.29	-0.01
0.543	-0.94	-0.05	0.35	-0.02
0.544	-0.92	0.01	0.39	-0.02
0.546	-0.87	0.12	0.48	-0.05
0.548	-0.75	0.30	0.58	-0.06
0.550	-0.59	0.46	0.67	-0.06
0.551	-0.38	0.60	0.70	-0.06
0.553	-0.20	0.71	0.68	-0.03
0.555	-0.10	0.73	0.67	-0.02
0.557	-0.09	0.81	0.58	-0.10
0.559	-0.10	0.82	0.56	-0.11
0.560	-0.10	0.83	0.53	-0.14
0.562	-0.06	0.84	0.51	-0.18
0.564	-0.06	0.86	0.46	-0.22
0.566	-0.06	0.88	0.43	-0.20
0.567	-0.10	0.91	0.37	-0.16
0.569	-0.11	0.93	0.33	-0.13
0.571	-0.10	0.91	0.33	-0.23
0.573	-0.09	0.94	0.26	-0.20
0.574	-0.08	0.97	0.19	-0.15
0.576	-0.09	0.97	0.18	-0.13
0.578	-0.13	0.97	0.18	-0.13

0.580	-0.14	0.97	0.15	-0.12
0.582	-0.13	0.98	0.12	-0.09
0.583	-0.08	0.99	0.11	-0.05
0.585	-0.05	0.99	0.12	0.04
0.587	0.01	0.99	0.08	0.10
0.589	0.05	0.98	0.04	0.16
0.590	0.07	0.97	0.02	0.23
0.592	0.09	0.94	0.01	0.32
0.594	0.11	0.87	-0.01	0.48
0.596	0.13	0.78	-0.07	0.61
0.598	0.15	0.70	-0.11	0.69
0.599	0.17	0.62	-0.14	0.75
0.601	0.16	0.55	-0.17	0.80
0.603	0.14	0.50	-0.19	0.83
0.605	0.15	0.46	-0.20	0.85
0.606	0.15	0.43	-0.21	0.87
0.608	0.15	0.41	-0.21	0.87
0.610	0.14	0.40	-0.22	0.88
0.612	0.13	0.40	-0.22	0.88
0.613	0.13	0.40	-0.21	0.89
0.615	0.13	0.39	-0.21	0.89
0.617	0.13	0.39	-0.21	0.89
0.619	0.13	0.40	-0.20	0.89
0.621	0.14	0.41	-0.19	0.88
0.622	0.14	0.42	-0.19	0.88
0.624	0.14	0.42	-0.18	0.88
0.626	0.13	0.42	-0.18	0.88
0.628	0.13	0.43	-0.18	0.88
0.629	0.14	0.43	-0.17	0.88
0.631	0.14	0.43	-0.17	0.87
0.633	0.14	0.44	-0.17	0.87
0.635	0.14	0.45	-0.17	0.87
0.637	0.15	0.45	-0.17	0.86
0.638	0.14	0.46	-0.17	0.86
0.640	0.14	0.48	-0.17	0.85
0.642	0.13	0.49	-0.17	0.85
0.644	0.13	0.51	-0.16	0.84
0.645	0.12	0.54	-0.15	0.82
0.647	0.11	0.59	-0.14	0.79
0.649	0.09	0.63	-0.13	0.76
0.651	0.08	0.69	-0.10	0.71

0.652	0.04	0.74	-0.09	0.67
0.654	0.03	0.79	-0.07	0.61
0.656	0.02	0.83	-0.06	0.55
0.658	-0.00	0.88	-0.02	0.48
0.660	-0.03	0.91	0.01	0.41
0.661	-0.08	0.94	0.01	0.34
0.663	-0.07	0.95	-0.02	0.31
0.665	-0.09	0.95	-0.04	0.29
0.667	-0.10	0.95	-0.05	0.28
0.668	-0.17	0.95	-0.03	0.27
0.670	-0.17	0.96	-0.04	0.24
0.672	-0.19	0.96	-0.04	0.22
0.674	-0.23	0.94	-0.04	0.25
0.676	-0.25	0.93	-0.06	0.27
0.677	-0.27	0.90	-0.07	0.32
0.679	-0.29	0.89	-0.09	0.35
0.681	-0.29	0.87	-0.12	0.38
0.683	-0.28	0.87	-0.14	0.38
0.684	-0.30	0.84	-0.11	0.44
0.686	-0.28	0.84	-0.13	0.45
0.688	-0.23	0.83	-0.17	0.48
0.690	-0.21	0.84	-0.19	0.47
0.691	-0.20	0.84	-0.18	0.47
0.693	-0.20	0.83	-0.17	0.49
0.695	-0.18	0.82	-0.17	0.51
0.697	-0.16	0.83	-0.18	0.51
0.699	-0.19	0.79	-0.26	0.52
0.700	-0.19	0.81	-0.26	0.49
0.702	-0.26	0.80	-0.28	0.46
0.704	-0.36	0.77	-0.32	0.41
0.706	-0.45	0.74	-0.35	0.35
0.707	-0.51	0.73	-0.36	0.29
0.709	-0.58	0.69	-0.35	0.27
0.711	-0.63	0.64	-0.34	0.27
0.713	-0.69	0.61	-0.35	0.18
0.715	-0.71	0.60	-0.33	0.16
0.716	-0.72	0.60	-0.31	0.17
0.718	-0.76	0.56	-0.29	0.15
0.720	-0.78	0.54	-0.26	0.14
0.722	-0.81	0.52	-0.23	0.14
0.723	-0.85	0.48	-0.17	0.14

0.725	-0.87	0.45	-0.14	0.13
0.727	-0.92	0.36	-0.06	0.12
0.729	-0.95	0.30	-0.04	0.11
0.730	-0.98	0.20	-0.02	0.09
0.732	-0.98	0.16	-0.01	0.07
0.734	-0.98	0.18	0.01	0.06
0.736	-0.97	0.24	0.04	0.06
0.738	-0.95	0.31	0.06	0.06
0.739	-0.93	0.35	0.05	0.06
0.741	-0.93	0.34	0.11	0.02
0.743	-0.92	0.38	0.09	-0.00
0.745	-0.91	0.42	0.06	-0.02
0.746	-0.90	0.42	0.07	-0.02
0.748	-0.91	0.41	0.09	-0.02
0.750	-0.91	0.40	0.10	-0.02
0.752	-0.91	0.40	0.07	-0.03
0.754	-0.92	0.39	0.04	-0.02
0.755	-0.93	0.38	0.02	-0.03
0.757	-0.95	0.32	0.03	0.01
0.759	-0.97	0.24	0.03	0.07
0.761	-0.98	0.17	0.01	0.11
0.762	-0.98	0.14	-0.03	0.15
0.764	-0.97	0.12	-0.07	0.20
0.766	-0.95	0.12	-0.10	0.27
0.768	-0.93	0.11	-0.11	0.34
0.770	-0.89	0.09	-0.15	0.41
0.771	-0.88	0.10	-0.19	0.43
0.773	-0.86	0.20	-0.22	0.43
0.775	-0.88	0.20	-0.18	0.39
0.777	-0.93	0.15	-0.13	0.32
0.778	-0.93	0.17	-0.16	0.29
0.780	-0.96	0.19	-0.11	0.20
0.782	-0.99	0.10	0.01	0.10
0.784	-1.00	0.05	0.03	0.07
0.785	-1.00	0.02	0.01	-0.01
0.787	-1.00	-0.04	0.01	-0.06
0.789	-0.99	-0.09	0.04	-0.10
0.791	-0.98	-0.14	0.07	-0.13
0.793	-0.98	-0.12	0.10	-0.16
0.794	-0.97	-0.06	0.11	-0.19
0.796	-0.97	-0.01	0.12	-0.19

0.798	-0.98	0.00	0.12	-0.18
0.800	-0.98	-0.01	0.13	-0.17
0.801	-0.98	-0.05	0.12	-0.15
0.803	-0.98	-0.08	0.12	-0.14
0.805	-0.98	-0.10	0.11	-0.14
0.807	-0.98	-0.11	0.11	-0.13
0.809	-0.98	-0.13	0.11	-0.13
0.810	-0.98	-0.13	0.11	-0.13
0.812	-0.98	-0.15	0.11	-0.12
0.814	-0.98	-0.15	0.11	-0.10
0.816	-0.98	-0.15	0.11	-0.10
0.817	-0.98	-0.14	0.11	-0.11
0.819	-0.98	-0.11	0.13	-0.13
0.821	-0.97	-0.07	0.15	-0.15
0.823	-0.97	-0.02	0.17	-0.16
0.824	-0.97	0.01	0.18	-0.16
0.826	-0.94	0.09	0.24	-0.21
0.828	-0.90	0.26	0.23	-0.25
0.830	-0.76	0.52	0.19	-0.33
0.832	-0.61	0.71	0.11	-0.35
0.833	-0.48	0.81	0.07	-0.33
0.835	-0.35	0.88	0.05	-0.31
0.837	-0.26	0.92	0.06	-0.30
0.839	-0.20	0.95	0.07	-0.25
0.840	-0.10	0.96	0.08	-0.24
0.842	-0.10	0.96	0.08	-0.23
0.844	-0.08	0.97	0.07	-0.21
0.846	-0.05	0.98	0.09	-0.19
0.848	-0.03	0.98	0.10	-0.19
0.849	-0.04	0.97	0.10	-0.19
0.851	-0.07	0.97	0.13	-0.19
0.853	-0.08	0.96	0.17	-0.19
0.855	-0.11	0.96	0.15	-0.20
0.856	-0.13	0.96	0.16	-0.21
0.858	-0.17	0.95	0.18	-0.21
0.860	-0.21	0.94	0.18	-0.21
0.862	-0.25	0.93	0.15	-0.21
0.863	-0.28	0.93	0.13	-0.22
0.865	-0.28	0.93	0.11	-0.20
0.867	-0.28	0.93	0.11	-0.20
0.869	-0.33	0.91	0.03	-0.23

0.871	-0.36	0.91	-0.03	-0.22
0.872	-0.40	0.88	-0.10	-0.22
0.874	-0.49	0.83	-0.18	-0.19
0.876	-0.59	0.73	-0.34	-0.02
0.878	-0.66	0.53	-0.53	0.06
0.879	-0.64	0.20	-0.72	0.17
0.881	-0.54	0.03	-0.80	0.26
0.883	-0.37	-0.33	-0.77	0.40
0.885	-0.31	-0.38	-0.77	0.41
0.887	-0.28	-0.42	-0.76	0.40
0.888	-0.22	-0.48	-0.74	0.41
0.890	-0.19	-0.53	-0.72	0.41
0.892	-0.17	-0.55	-0.70	0.42
0.894	-0.14	-0.55	-0.70	0.44
0.895	-0.13	-0.53	-0.69	0.48
0.897	-0.11	-0.51	-0.68	0.51
0.899	-0.11	-0.48	-0.68	0.54
0.901	-0.14	-0.50	-0.65	0.55
0.902	-0.17	-0.49	-0.65	0.55
0.904	-0.17	-0.40	-0.68	0.59
0.906	-0.22	-0.35	-0.65	0.64
0.908	-0.35	-0.30	-0.63	0.63
0.910	-0.47	-0.23	-0.64	0.56
0.911	-0.55	-0.27	-0.59	0.52
0.913	-0.64	-0.25	-0.54	0.49
0.915	-0.70	-0.20	-0.49	0.47
0.917	-0.78	-0.19	-0.45	0.39
0.918	-0.84	-0.20	-0.38	0.33
0.920	-0.88	-0.25	-0.28	0.29
0.922	-0.91	-0.24	-0.25	0.23
0.924	-0.92	-0.19	-0.28	0.21
0.926	-0.90	-0.23	-0.28	0.23
0.927	-0.90	-0.27	-0.25	0.23
0.929	-0.92	-0.26	-0.20	0.23
0.931	-0.95	-0.18	-0.15	0.21
0.933	-0.97	-0.10	-0.14	0.16
0.934	-0.98	-0.07	-0.15	0.13
0.936	-0.97	-0.12	-0.16	0.11
0.938	-0.96	-0.24	-0.11	0.11
0.940	-0.89	-0.16	-0.16	0.40
0.941	-0.92	-0.13	-0.26	0.25

0.943	-0.84	-0.09	-0.54	0.03
0.945	-0.48	-0.33	-0.80	-0.13
0.947	-0.38	-0.65	-0.66	-0.00
0.949	0.03	-0.87	-0.37	0.32
0.950	0.60	-0.65	-0.33	0.33
0.952	0.94	-0.25	-0.17	0.14
0.954	-0.10	-0.86	-0.24	-0.43
0.956	0.29	-0.64	-0.51	-0.50
0.957	0.55	-0.29	-0.69	-0.37
0.959	0.70	-0.20	-0.64	-0.23
0.961	0.72	-0.41	-0.55	-0.06
0.963	0.51	-0.79	-0.31	0.14
0.965	0.08	-0.93	-0.34	0.14
0.966	0.17	-0.78	-0.60	0.09
0.968	0.41	-0.49	-0.74	-0.20
0.970	0.56	-0.45	-0.67	-0.18
0.972	0.59	-0.55	-0.53	-0.25
0.973	0.50	-0.72	-0.43	-0.20
0.975	0.48	-0.78	-0.32	-0.22
0.977	0.39	-0.83	-0.31	-0.24
0.979	0.49	-0.72	-0.34	-0.37
0.980	0.61	-0.47	-0.37	-0.52
0.982	0.34	-0.56	-0.73	-0.19
0.984	0.48	-0.39	-0.71	-0.32
0.986	0.54	-0.29	-0.65	-0.45
0.988	0.66	-0.12	-0.60	-0.44
0.989	0.60	-0.28	-0.73	-0.15
0.991	0.55	-0.33	-0.76	-0.08
0.993	0.50	-0.61	-0.61	-0.00
0.995	0.45	-0.71	-0.54	0.00
0.996	-0.82	-0.46	-0.19	-0.29
0.998	-0.76	0.53	0.20	-0.33
1.000	-0.56	0.74	0.31	-0.22
0.000	0.78	-0.54	-0.28	0.16
0.002	0.46	-0.75	-0.43	-0.20
0.004	0.80	-0.43	-0.17	-0.38
0.005	0.52	-0.61	-0.60	0.04
0.007	0.61	-0.67	-0.42	0.07
0.009	0.65	-0.63	-0.38	0.20
0.011	0.66	-0.65	-0.31	-0.22
0.012	0.75	-0.50	-0.32	0.29

0.014	0.80	-0.41	-0.44	0.03
0.016	0.68	-0.42	-0.54	-0.27
0.018	0.74	-0.60	-0.19	-0.22
0.020	0.81	-0.55	-0.15	0.11
0.021	0.84	-0.44	-0.21	0.23
0.023	0.66	-0.57	-0.40	0.29
0.025	0.28	-0.65	-0.48	0.52
0.027	0.28	-0.51	-0.40	0.71
0.028	-0.01	-0.47	-0.71	0.53
0.030	0.20	-0.41	-0.81	0.36
0.032	0.28	0.12	-0.71	0.64
0.034	0.27	-0.07	-0.82	0.49
0.035	0.22	-0.09	-0.81	0.54
0.037	0.13	0.01	-0.81	0.57
0.039	0.03	0.10	-0.87	0.49
0.041	-0.05	0.15	-0.93	0.33
0.043	0.04	0.15	-0.95	0.28
0.044	0.27	0.17	-0.91	0.26
0.046	0.49	0.21	-0.81	0.24
0.048	0.61	0.25	-0.71	0.23
0.050	0.67	0.27	-0.66	0.22
0.051	0.67	0.26	-0.65	0.24
0.053	0.68	0.24	-0.64	0.28
0.055	0.68	0.16	-0.65	0.30
0.057	0.71	0.05	-0.64	0.29
0.059	0.76	-0.10	-0.58	0.27
0.060	0.81	-0.28	-0.46	0.21
0.062	0.83	-0.43	-0.31	0.18
0.064	0.83	-0.53	-0.17	0.10
0.066	0.84	-0.52	-0.13	0.08
0.067	0.85	-0.52	-0.08	0.08
0.069	0.85	-0.52	-0.03	0.04
0.071	0.86	-0.50	-0.01	0.03
0.073	0.88	-0.47	0.01	0.03
0.074	0.89	-0.46	0.04	0.02
0.076	0.90	-0.43	0.03	0.06
0.078	0.91	-0.39	0.05	0.11
0.080	0.92	-0.35	0.02	0.16
0.082	0.94	-0.29	0.01	0.20
0.083	0.94	-0.22	-0.00	0.27
0.085	0.94	-0.16	0.01	0.30

0.087	0.95	-0.09	0.05	0.29
0.089	0.95	-0.05	0.10	0.28
0.090	0.97	-0.08	0.16	0.18
0.092	0.95	-0.24	0.21	-0.04
0.094	0.84	-0.43	0.25	-0.24
0.096	0.74	-0.48	0.30	-0.36
0.098	0.78	-0.41	0.31	-0.36
0.099	0.81	-0.39	0.28	-0.34
0.101	0.85	-0.39	0.24	-0.28
0.103	0.88	-0.38	0.22	-0.15
0.105	0.89	-0.39	0.24	-0.03
0.106	0.89	-0.37	0.25	0.05
0.108	0.90	-0.35	0.25	0.12
0.110	0.89	-0.32	0.25	0.20
0.112	0.89	-0.30	0.26	0.24
0.113	0.88	-0.29	0.26	0.25
0.115	0.88	-0.27	0.25	0.28
0.117	0.88	-0.26	0.24	0.32
0.119	0.87	-0.27	0.24	0.34
0.121	0.87	-0.26	0.23	0.34
0.122	0.87	-0.25	0.23	0.34
0.124	0.88	-0.25	0.22	0.34
0.126	0.89	-0.23	0.21	0.33
0.128	0.90	-0.21	0.21	0.32
0.129	0.91	-0.20	0.21	0.29
0.131	0.93	-0.18	0.21	0.25
0.133	0.93	-0.16	0.21	0.24
0.135	0.95	-0.10	0.19	0.21
0.137	0.96	-0.02	0.20	0.21
0.138	0.96	-0.00	0.19	0.21
0.140	0.95	-0.02	0.20	0.23
0.142	0.94	-0.03	0.23	0.23
0.144	0.94	-0.07	0.25	0.23
0.145	0.93	-0.08	0.27	0.23
0.147	0.93	-0.02	0.28	0.23
0.149	0.94	-0.01	0.27	0.22
0.151	0.94	-0.01	0.27	0.21
0.152	0.93	0.02	0.26	0.24
0.154	0.93	0.03	0.27	0.26
0.156	0.92	0.03	0.29	0.25
0.158	0.93	0.05	0.28	0.25

0.160	0.92	0.07	0.28	0.25
0.161	0.92	0.06	0.30	0.24
0.163	0.92	0.08	0.30	0.23
0.165	0.93	0.10	0.29	0.22
0.167	0.93	0.16	0.26	0.20
0.168	0.92	0.24	0.22	0.20
0.170	0.91	0.33	0.14	0.20
0.172	0.89	0.40	0.10	0.19
0.174	0.89	0.42	0.07	0.17
0.176	0.91	0.40	0.03	0.14
0.177	0.93	0.35	-0.00	0.10
0.179	0.95	0.29	-0.05	0.12
0.181	0.97	0.17	-0.13	0.14
0.183	0.97	0.06	-0.19	0.14
0.184	0.96	-0.04	-0.23	0.16
0.186	0.95	-0.09	-0.25	0.16
0.188	0.95	-0.13	-0.25	0.13
0.190	0.95	-0.14	-0.25	0.13
0.191	0.95	-0.15	-0.21	0.19
0.193	0.95	-0.16	-0.17	0.20
0.195	0.95	-0.19	-0.11	0.23
0.197	0.96	-0.17	-0.06	0.24
0.199	0.96	-0.18	-0.01	0.22
0.200	0.96	-0.20	0.01	0.22
0.202	0.96	-0.20	0.05	0.20
0.204	0.96	-0.19	0.06	0.21
0.206	0.94	-0.24	0.12	0.21
0.207	0.95	-0.25	0.12	0.17
0.209	0.94	-0.27	0.17	0.13
0.211	0.93	-0.27	0.22	0.06
0.213	0.93	-0.26	0.26	-0.01
0.215	0.93	-0.23	0.28	-0.05
0.216	0.93	-0.20	0.29	-0.09
0.218	0.92	-0.20	0.31	-0.14
0.220	0.92	-0.13	0.29	-0.21
0.222	0.90	-0.06	0.30	-0.30
0.223	0.87	0.04	0.33	-0.36
0.225	0.83	0.10	0.39	-0.39
0.227	0.75	0.17	0.47	-0.44
0.229	0.67	0.19	0.58	-0.43
0.230	0.62	0.15	0.67	-0.37

0.232	0.58	0.10	0.72	-0.36
0.234	0.56	-0.00	0.76	-0.31
0.236	0.55	-0.05	0.79	-0.27
0.238	0.50	-0.06	0.82	-0.26
0.239	0.47	-0.04	0.83	-0.28
0.241	0.49	-0.06	0.84	-0.22
0.243	0.48	-0.09	0.84	-0.24
0.245	0.45	-0.12	0.84	-0.27
0.246	0.44	-0.14	0.85	-0.24
0.248	0.50	-0.18	0.80	-0.28
0.250	0.51	-0.19	0.79	-0.27
0.252	0.52	-0.21	0.79	-0.24
0.254	0.52	-0.24	0.79	-0.21
0.255	0.51	-0.27	0.79	-0.22
0.257	0.52	-0.28	0.78	-0.21
0.259	0.52	-0.32	0.75	-0.24
0.261	0.49	-0.36	0.76	-0.23
0.262	0.44	-0.39	0.77	-0.24
0.264	0.46	-0.40	0.76	-0.21
0.266	0.41	-0.40	0.78	-0.26
0.268	0.37	-0.40	0.79	-0.28
0.270	0.40	-0.38	0.80	-0.24
0.271	0.42	-0.37	0.79	-0.25
0.273	0.44	-0.34	0.79	-0.27
0.275	0.45	-0.33	0.79	-0.25
0.277	0.45	-0.33	0.81	-0.20
0.278	0.48	-0.31	0.80	-0.17
0.280	0.50	-0.30	0.79	-0.16
0.282	0.52	-0.27	0.80	-0.16
0.284	0.56	-0.23	0.77	-0.19
0.285	0.62	-0.12	0.76	-0.17
0.287	0.66	0.05	0.72	-0.18
0.289	0.67	0.24	0.67	-0.22
0.291	0.52	0.38	0.69	-0.34
0.293	0.38	0.58	0.60	-0.40
0.294	0.31	0.63	0.60	-0.38
0.296	0.46	0.63	0.44	-0.45
0.298	0.32	0.84	-0.17	-0.41
0.300	0.39	0.88	-0.22	-0.13
0.301	0.43	0.90	-0.10	-0.00
0.303	0.18	0.87	-0.13	0.45

0.305	0.29	0.48	-0.31	0.76
0.307	0.32	0.60	-0.08	0.73
0.309	0.58	0.56	-0.14	0.58
0.310	0.47	0.44	-0.28	0.71
0.312	0.28	0.26	-0.32	0.86
0.314	0.30	0.24	-0.30	0.87
0.316	0.34	0.28	-0.27	0.86
0.317	0.28	0.27	-0.31	0.87
0.319	0.29	0.25	-0.24	0.89
0.321	0.28	0.29	-0.18	0.90
0.323	0.34	0.31	-0.15	0.88
0.324	0.38	0.33	-0.21	0.83
0.326	0.36	0.35	-0.30	0.81
0.328	0.39	0.43	-0.27	0.77
0.330	0.37	0.61	-0.20	0.66
0.332	0.28	0.78	-0.22	0.51
0.333	0.48	0.80	-0.26	-0.25
0.335	-0.06	0.84	-0.44	-0.31
0.337	0.41	0.65	-0.57	-0.29
0.339	0.74	0.48	-0.40	-0.25
0.340	0.56	0.50	-0.58	-0.31
0.342	0.66	0.56	-0.48	-0.13
0.344	0.78	0.58	-0.21	-0.09
0.346	0.70	0.69	0.19	-0.02
0.348	0.19	0.70	-0.66	-0.18
0.349	0.17	0.71	-0.51	-0.45
0.351	0.01	0.81	-0.32	-0.49
0.353	-0.15	0.85	-0.40	-0.30
0.355	-0.29	0.73	-0.55	0.29
0.356	-0.26	0.61	-0.37	0.65
0.358	-0.06	0.85	0.17	0.50
0.360	-0.18	0.96	0.09	0.22
0.362	-0.07	0.93	-0.19	0.29
0.363	-0.12	0.73	-0.65	0.18
0.365	-0.45	0.75	-0.48	-0.11
0.367	-0.62	0.78	-0.00	-0.01
0.369	-0.84	0.49	-0.11	0.21
0.371	-0.83	0.47	-0.30	0.05
0.372	-0.58	0.59	-0.27	-0.49
0.374	-0.66	0.41	-0.12	-0.62
0.376	-0.60	0.78	-0.20	-0.04

0.378	-0.77	0.55	-0.18	-0.27
0.379	-0.90	0.26	0.03	-0.36
0.381	-0.95	0.06	0.10	-0.30
0.383	-0.98	-0.13	0.09	-0.14
0.385	-0.96	-0.03	-0.03	0.29
0.387	-0.91	0.08	0.06	0.40
0.388	-0.91	-0.12	0.23	0.34
0.390	-0.92	-0.35	-0.06	-0.14
0.392	-0.96	-0.19	-0.05	-0.21
0.394	-0.97	-0.21	0.08	-0.11
0.395	-0.97	-0.22	0.09	0.02
0.397	-0.95	-0.29	0.02	0.08
0.399	-0.96	-0.18	0.07	0.18
0.401	-0.94	-0.11	0.18	0.26
0.402	-0.94	-0.18	0.11	0.27
0.404	-0.92	-0.06	0.08	0.38
0.406	-0.71	-0.02	0.24	0.67
0.408	-0.58	0.06	0.37	0.73
0.410	-0.61	-0.08	0.19	0.77
0.411	-0.55	-0.26	0.05	0.79
0.413	-0.36	-0.10	0.25	0.90
0.415	-0.35	0.15	0.34	0.86
0.417	-0.58	-0.42	-0.36	0.60
0.418	-0.84	0.05	-0.03	0.54
0.420	-0.81	0.05	-0.10	0.58
0.422	-0.82	-0.17	0.02	0.55
0.424	-0.80	-0.21	0.05	0.56
0.426	-0.71	0.18	0.04	0.68
0.427	-0.75	0.45	0.15	0.47
0.429	-0.75	0.52	0.02	0.41
0.431	-0.66	0.27	-0.23	0.66
0.433	-0.74	0.65	-0.02	0.15
0.434	-0.75	0.54	0.25	0.30
0.436	-0.59	0.45	0.17	0.65
0.438	-0.62	0.50	0.04	0.60
0.440	-0.67	0.59	0.10	0.44
0.441	-0.62	0.52	0.06	0.59
0.443	-0.47	0.37	0.11	0.79
0.445	-0.64	0.22	0.31	0.67
0.447	-0.62	0.21	0.07	0.75
0.449	-0.80	0.14	0.06	0.57

0.450	-0.63	0.25	0.11	0.72
0.452	-0.44	0.60	0.16	0.65
0.454	-0.46	0.65	0.38	0.47
0.456	-0.32	0.53	0.29	0.73
0.457	-0.36	0.38	0.22	0.82
0.459	-0.80	0.20	0.31	0.47
0.461	-0.74	0.04	0.12	0.66
0.463	-0.73	-0.19	0.01	0.65
0.465	-0.83	0.06	-0.05	0.56
0.466	-0.76	0.23	0.04	0.60
0.468	-0.53	-0.11	-0.49	0.68
0.470	-0.55	0.32	-0.38	0.67
0.472	-0.75	0.64	-0.04	0.15
0.473	-0.96	0.16	-0.07	-0.21

Variable	Factor Loadings (Varimax normalized, data from RP-18 chromatographic plates) Extraction: Principal components (Marked loadings are >.70)			
	Factor 1	Factor 2	Factor 3	Factor 4
0.000	0.20	0.02	-0.98	0.09
0.002	-0.56	-0.21	-0.77	0.22
0.004	-0.39	-0.00	-0.92	0.08
0.005	-0.04	-0.17	-0.91	0.38
0.007	-0.00	-0.19	-0.88	0.45
0.009	-0.14	0.07	-0.97	0.18
0.011	0.00	0.17	-0.99	0.02
0.012	0.21	0.02	-0.97	0.12
0.014	0.38	-0.13	-0.88	0.25
0.016	0.16	-0.01	-0.98	0.10
0.018	0.54	-0.75	-0.28	0.26
0.020	0.20	-0.59	0.64	0.46
0.021	0.24	-0.56	0.57	0.55
0.023	0.32	-0.63	0.52	0.49
0.025	0.46	-0.63	0.46	0.42
0.027	0.64	-0.54	0.35	0.42
0.028	0.80	-0.37	0.24	0.42
0.030	0.94	-0.02	0.11	0.33
0.032	0.34	0.68	-0.40	-0.52
0.034	-0.17	0.58	-0.44	-0.66
0.035	-0.27	0.64	-0.40	-0.61
0.037	-0.34	0.61	-0.34	-0.63

0.039	-0.44	0.42	-0.34	-0.71
0.041	-0.44	0.36	-0.38	-0.73
0.043	-0.38	0.37	-0.46	-0.71
0.044	-0.32	0.36	-0.55	-0.68
0.046	-0.21	0.33	-0.51	-0.77
0.048	-0.19	0.36	-0.57	-0.71
0.050	-0.07	0.38	-0.57	-0.73
0.051	0.01	0.57	-0.51	-0.64
0.053	-0.08	0.72	-0.44	-0.53
0.055	-0.15	0.55	-0.21	-0.80
0.057	-0.07	0.09	-0.15	-0.98
0.059	-0.13	0.01	-0.67	-0.73
0.060	-0.32	0.01	-0.72	-0.62
0.062	-0.61	-0.31	-0.35	-0.64
0.064	-0.58	-0.41	-0.41	-0.57
0.066	-0.37	-0.19	-0.62	-0.67
0.067	-0.33	-0.13	-0.40	-0.85
0.069	-0.18	-0.21	-0.34	-0.90
0.071	-0.01	-0.20	-0.44	-0.87
0.073	-0.06	-0.09	-0.26	-0.96
0.074	-0.33	-0.16	-0.19	-0.91
0.076	-0.09	-0.47	-0.36	-0.80
0.078	0.37	-0.30	-0.33	-0.81
0.080	0.59	-0.29	-0.29	-0.69
0.082	0.45	-0.57	-0.41	-0.55
0.083	0.45	-0.52	-0.46	-0.56
0.085	0.47	-0.46	-0.45	-0.61
0.087	0.28	-0.62	-0.48	-0.56
0.089	0.20	-0.40	-0.60	-0.66
0.090	-0.02	-0.21	-0.52	-0.83
0.092	-0.40	-0.22	-0.35	-0.82
0.094	-0.51	-0.21	-0.43	-0.72
0.096	-0.40	0.01	-0.62	-0.67
0.098	-0.51	0.11	-0.46	-0.72
0.099	-0.67	0.05	-0.20	-0.71
0.101	-0.69	0.17	-0.21	-0.67
0.103	-0.72	0.19	-0.07	-0.66
0.105	-0.65	0.26	-0.31	-0.64
0.106	-0.56	0.06	-0.48	-0.68
0.108	-0.56	-0.27	-0.41	-0.67
0.110	-0.62	-0.20	-0.28	-0.71

0.112	-0.39	0.10	-0.09	-0.91
0.113	-0.15	0.09	-0.26	-0.95
0.115	-0.16	-0.05	-0.62	-0.77
0.117	0.01	0.13	-0.66	-0.74
0.119	0.02	0.05	-0.80	-0.60
0.121	-0.16	0.07	-0.71	-0.68
0.122	-0.27	0.09	-0.60	-0.75
0.124	-0.06	0.08	-0.53	-0.84
0.126	0.07	0.17	-0.27	-0.95
0.128	-0.07	0.25	-0.33	-0.91
0.129	-0.19	0.09	-0.62	-0.75
0.131	-0.28	0.20	-0.18	-0.92
0.133	-0.22	0.18	-0.33	-0.90
0.135	-0.27	0.20	-0.75	-0.57
0.137	-0.37	0.12	-0.77	-0.50
0.138	-0.61	0.04	-0.52	-0.59
0.140	-0.92	0.05	0.34	-0.19
0.142	-0.81	0.53	0.25	-0.00
0.144	-0.72	0.64	-0.14	-0.22
0.145	-0.53	0.54	0.29	-0.59
0.147	-0.26	0.26	0.58	-0.73
0.149	-0.25	0.60	-0.17	-0.75
0.151	-0.64	0.44	-0.49	-0.38
0.152	-0.90	0.08	0.14	-0.40
0.154	-0.84	0.31	-0.06	-0.45
0.156	-0.54	0.62	-0.39	-0.42
0.158	-0.40	0.69	0.06	-0.60
0.160	-0.89	-0.32	0.29	-0.14
0.161	-0.89	0.33	-0.30	0.09
0.163	-0.13	0.66	-0.48	-0.56
0.165	0.05	0.37	-0.27	-0.89
0.167	-0.78	0.36	-0.15	-0.49
0.168	-0.53	0.81	-0.06	-0.24
0.170	0.35	0.93	0.06	-0.14
0.172	-0.70	-0.07	0.03	0.71
0.174	-0.88	0.08	0.11	0.45
0.176	-0.95	0.30	-0.03	0.00
0.177	-0.87	0.10	0.19	-0.45
0.179	-0.70	-0.15	0.21	-0.66
0.181	-0.61	-0.18	-0.21	-0.74
0.183	-0.30	-0.29	-0.44	-0.80

0.184	0.45	-0.78	0.03	-0.45
0.186	0.40	-0.90	-0.01	-0.16
0.188	-0.25	-0.75	0.51	0.34
0.190	-0.09	-0.88	-0.00	-0.47
0.191	0.34	-0.83	-0.44	0.07
0.193	0.47	-0.38	-0.30	0.74
0.195	0.10	-0.33	0.32	0.88
0.197	-0.33	0.04	0.67	0.67
0.199	-0.45	0.17	0.56	0.68
0.200	-0.78	0.11	0.46	0.42
0.202	-0.50	0.42	-0.12	0.74
0.204	-0.66	0.11	0.47	0.58
0.206	-0.67	-0.15	0.43	0.59
0.207	-0.65	-0.49	-0.11	0.57
0.209	-0.36	-0.71	-0.51	-0.34
0.211	-0.40	-0.48	-0.35	-0.70
0.213	-0.51	-0.44	-0.41	-0.61
0.215	-0.13	-0.72	-0.53	-0.42
0.216	-0.25	-0.04	-0.55	-0.79
0.218	-0.14	-0.48	-0.39	-0.78
0.220	-0.60	-0.40	-0.04	-0.69
0.222	-0.68	-0.44	0.01	-0.59
0.223	-0.30	-0.71	-0.08	-0.63
0.225	0.43	0.23	0.75	-0.45
0.227	-0.02	0.53	0.74	0.41
0.229	-0.25	-0.95	0.14	0.10
0.230	-0.79	-0.52	0.29	-0.13
0.232	-0.44	-0.26	-0.69	-0.50
0.234	0.40	0.86	0.01	-0.31
0.236	0.48	0.71	0.49	0.17
0.238	0.36	0.39	0.76	0.39
0.239	0.24	0.41	0.86	0.18
0.241	0.22	0.46	0.86	0.10
0.243	-0.15	0.06	0.99	-0.03
0.245	0.61	-0.17	-0.12	0.76
0.246	0.04	0.26	-0.79	0.56
0.248	-0.05	0.79	-0.60	0.12
0.250	0.26	0.88	-0.39	-0.02
0.252	-0.63	-0.69	0.17	-0.31
0.254	-0.87	-0.11	0.03	-0.47
0.255	-0.86	0.30	-0.10	-0.41

0.257	-0.87	0.36	-0.23	-0.24
0.259	-0.86	0.10	-0.14	-0.47
0.261	-0.84	-0.06	-0.29	-0.45
0.262	-0.70	0.12	-0.32	-0.63
0.264	-0.63	0.19	-0.22	-0.72
0.266	-0.67	0.04	-0.12	-0.74
0.268	-0.66	0.08	-0.08	-0.74
0.270	-0.60	0.15	-0.00	-0.78
0.271	-0.68	-0.21	0.15	-0.69
0.273	-0.79	0.15	-0.14	-0.58
0.275	-0.76	0.29	0.14	-0.56
0.277	-0.56	0.24	0.09	-0.79
0.278	-0.66	-0.04	-0.49	-0.57
0.280	-0.64	-0.05	-0.70	0.31
0.282	-0.20	0.54	-0.66	-0.47
0.284	0.53	0.57	-0.00	-0.62
0.285	0.70	0.68	0.08	-0.21
0.287	0.16	0.88	0.42	0.13
0.289	-0.01	0.90	0.42	-0.11
0.291	-0.04	0.91	0.39	-0.14
0.293	0.33	0.63	0.65	0.28
0.294	0.34	0.53	0.61	0.48
0.296	0.18	0.84	0.24	0.45
0.298	0.15	0.99	0.01	0.02
0.300	0.42	0.60	0.47	-0.49
0.301	0.52	0.75	0.40	-0.07
0.303	0.31	0.85	0.43	0.04
0.305	0.25	0.89	0.30	0.24
0.307	0.37	0.77	0.25	0.46
0.309	0.37	0.60	0.43	0.57
0.310	0.42	0.51	0.68	0.33
0.312	0.38	0.50	0.76	0.15
0.314	0.17	0.51	0.84	0.09
0.316	-0.01	0.80	0.56	0.22
0.317	0.17	0.95	0.05	0.25
0.319	0.41	0.87	-0.08	0.26
0.321	0.65	0.70	0.20	0.21
0.323	0.47	0.74	0.43	0.22
0.324	0.15	0.80	0.49	0.32
0.326	0.14	0.83	0.40	0.35
0.328	0.28	0.88	0.15	0.35

0.330	0.10	0.96	-0.13	0.24
0.332	0.22	0.95	-0.02	0.21
0.333	0.43	0.90	0.07	0.07
0.335	0.54	0.84	-0.08	0.02
0.337	0.53	0.82	-0.20	0.07
0.339	0.47	0.87	-0.12	0.09
0.340	0.50	0.86	-0.03	0.11
0.342	0.58	0.80	-0.05	0.14
0.344	0.61	0.74	0.04	0.28
0.346	0.59	0.68	0.16	0.40
0.348	0.62	0.56	0.21	0.52
0.349	0.61	0.46	0.26	0.59
0.351	0.53	0.41	0.39	0.63
0.353	0.48	0.31	0.46	0.67
0.355	0.47	0.22	0.42	0.75
0.356	0.42	0.20	0.39	0.80
0.358	0.59	0.22	0.35	0.69
0.360	0.64	0.24	0.35	0.64
0.362	0.68	0.33	0.30	0.59
0.363	0.67	0.44	0.21	0.55
0.365	0.64	0.51	0.17	0.55
0.367	0.70	0.51	0.14	0.48
0.369	0.79	0.49	-0.04	0.37
0.371	0.80	0.46	-0.23	0.30
0.372	0.80	0.48	-0.25	0.27
0.374	0.77	0.54	-0.20	0.27
0.376	0.83	0.47	-0.16	0.26
0.378	0.89	0.37	-0.12	0.25
0.379	0.91	0.32	-0.05	0.26
0.381	0.89	0.29	0.03	0.36
0.383	0.80	0.22	0.21	0.52
0.385	0.66	0.14	0.38	0.63
0.387	0.51	-0.02	0.53	0.68
0.388	0.43	-0.09	0.49	0.76
0.390	0.35	-0.07	0.50	0.79
0.392	0.30	0.02	0.52	0.80
0.394	0.27	0.11	0.51	0.81
0.395	0.31	0.22	0.49	0.79
0.397	0.32	0.43	0.46	0.71
0.399	0.26	0.63	0.39	0.62
0.401	0.30	0.72	0.31	0.54

0.402	0.31	0.72	0.31	0.54
0.404	0.27	0.75	0.29	0.53
0.406	0.20	0.76	0.34	0.52
0.408	0.13	0.76	0.37	0.51
0.410	0.08	0.81	0.31	0.49
0.411	0.09	0.85	0.25	0.46
0.413	0.13	0.85	0.27	0.43
0.415	0.11	0.90	0.20	0.38
0.417	0.10	0.90	0.19	0.39
0.418	0.15	0.88	0.19	0.40
0.420	0.20	0.89	0.15	0.39
0.422	0.23	0.90	0.06	0.36
0.424	0.31	0.89	-0.04	0.31
0.426	0.44	0.84	-0.12	0.29
0.427	0.52	0.78	-0.17	0.30
0.429	0.64	0.70	-0.22	0.23
0.431	0.66	0.70	-0.20	0.20
0.433	0.73	0.63	-0.19	0.18
0.434	0.77	0.56	-0.19	0.22
0.436	0.76	0.56	-0.17	0.27
0.438	0.72	0.60	-0.09	0.34
0.440	0.63	0.64	0.01	0.44
0.441	0.52	0.66	0.08	0.53
0.443	0.41	0.61	0.12	0.67
0.445	0.41	0.51	0.07	0.75
0.447	0.34	0.34	-0.02	0.87
0.449	0.28	0.19	-0.07	0.94
0.450	0.27	0.07	-0.11	0.96
0.452	0.22	-0.03	-0.12	0.97
0.454	0.15	-0.11	-0.13	0.97
0.456	0.12	-0.16	-0.13	0.97
0.457	0.12	-0.14	-0.13	0.97
0.459	0.15	-0.15	-0.09	0.97
0.461	0.17	-0.13	-0.03	0.98
0.463	0.17	-0.10	-0.03	0.98
0.465	0.17	-0.06	-0.06	0.98
0.466	0.22	-0.02	-0.02	0.98
0.468	0.30	0.04	0.03	0.95
0.470	0.38	0.12	-0.00	0.92
0.472	0.48	0.28	0.04	0.83
0.473	0.49	0.35	0.08	0.79

0.475	0.55	0.38	0.06	0.74
0.477	0.60	0.37	0.03	0.71
0.479	0.63	0.35	0.03	0.69
0.480	0.65	0.33	0.04	0.69
0.482	0.67	0.26	0.08	0.69
0.484	0.68	0.21	0.15	0.69
0.486	0.70	0.09	0.22	0.67
0.488	0.71	0.06	0.29	0.63
0.489	0.72	0.03	0.35	0.60
0.491	0.69	-0.03	0.45	0.57
0.493	0.65	-0.09	0.55	0.52
0.495	0.62	-0.12	0.63	0.46
0.496	0.61	-0.11	0.69	0.39
0.498	0.60	-0.08	0.73	0.32
0.500	0.58	-0.01	0.76	0.30
0.502	0.58	0.04	0.76	0.29
0.504	0.58	0.10	0.75	0.30
0.505	0.60	0.15	0.71	0.33
0.507	0.60	0.21	0.67	0.38
0.509	0.60	0.27	0.61	0.45
0.511	0.58	0.33	0.52	0.53
0.512	0.58	0.36	0.43	0.59
0.514	0.53	0.36	0.35	0.68
0.516	0.50	0.34	0.35	0.72
0.518	0.49	0.31	0.33	0.75
0.520	0.48	0.28	0.34	0.76
0.521	0.46	0.25	0.37	0.76
0.523	0.45	0.20	0.40	0.78
0.525	0.43	0.13	0.43	0.78
0.527	0.40	0.05	0.46	0.79
0.528	0.40	-0.03	0.47	0.79
0.530	0.43	-0.11	0.47	0.77
0.532	0.48	-0.18	0.46	0.73
0.534	0.54	-0.21	0.42	0.70
0.535	0.59	-0.21	0.37	0.69
0.537	0.63	-0.17	0.31	0.69
0.539	0.68	-0.11	0.24	0.69
0.541	0.73	-0.07	0.15	0.66
0.543	0.74	-0.03	0.06	0.67
0.544	0.74	-0.01	0.02	0.67
0.546	0.75	0.01	0.00	0.67

0.548	0.76	0.00	0.00	0.65
0.550	0.77	-0.01	-0.00	0.64
0.551	0.77	-0.04	-0.01	0.63
0.553	0.79	-0.07	0.01	0.61
0.555	0.80	-0.09	0.05	0.59
0.557	0.78	-0.10	0.08	0.61
0.559	0.79	-0.13	0.08	0.59
0.560	0.79	-0.12	0.10	0.59
0.562	0.79	-0.14	0.10	0.59
0.564	0.78	-0.22	0.08	0.58
0.566	0.78	-0.24	0.10	0.58
0.567	0.77	-0.23	0.11	0.59
0.569	0.77	-0.25	0.07	0.59
0.571	0.76	-0.26	0.04	0.59
0.573	0.75	-0.27	0.01	0.60
0.574	0.74	-0.30	0.01	0.60
0.576	0.74	-0.33	0.01	0.59
0.578	0.73	-0.35	-0.01	0.58
0.580	0.73	-0.36	-0.01	0.58
0.582	0.72	-0.38	-0.02	0.58
0.583	0.71	-0.38	-0.05	0.59
0.585	0.69	-0.38	-0.05	0.61
0.587	0.67	-0.39	-0.08	0.62
0.589	0.66	-0.39	-0.11	0.64
0.590	0.63	-0.39	-0.12	0.66
0.592	0.60	-0.39	-0.13	0.68
0.594	0.57	-0.39	-0.14	0.71
0.596	0.51	-0.39	-0.16	0.75
0.598	0.46	-0.39	-0.15	0.79
0.599	0.38	-0.38	-0.13	0.83
0.601	0.34	-0.38	-0.09	0.85
0.603	0.30	-0.37	-0.07	0.88
0.605	0.29	-0.37	-0.08	0.88
0.606	0.28	-0.36	-0.08	0.89
0.608	0.30	-0.31	-0.03	0.90
0.610	0.34	-0.28	-0.01	0.90
0.612	0.36	-0.31	-0.02	0.88
0.613	0.39	-0.30	-0.05	0.87
0.615	0.40	-0.29	-0.01	0.87
0.617	0.39	-0.24	-0.02	0.89
0.619	0.36	-0.25	-0.04	0.90

0.621	0.35	-0.31	-0.05	0.88
0.622	0.36	-0.33	-0.06	0.87
0.624	0.35	-0.32	-0.08	0.87
0.626	0.31	-0.34	-0.09	0.88
0.628	0.33	-0.33	-0.07	0.88
0.629	0.35	-0.34	-0.06	0.87
0.631	0.35	-0.35	-0.06	0.86
0.633	0.36	-0.36	-0.05	0.86
0.635	0.38	-0.37	-0.02	0.84
0.637	0.39	-0.36	-0.00	0.85
0.638	0.39	-0.35	0.03	0.85
0.640	0.41	-0.35	0.08	0.84
0.642	0.42	-0.37	0.13	0.82
0.644	0.45	-0.36	0.18	0.80
0.645	0.46	-0.34	0.22	0.78
0.647	0.48	-0.32	0.27	0.77
0.649	0.49	-0.32	0.34	0.74
0.651	0.50	-0.32	0.40	0.70
0.652	0.50	-0.33	0.41	0.69
0.654	0.53	-0.31	0.41	0.68
0.656	0.55	-0.28	0.42	0.66
0.658	0.56	-0.29	0.44	0.64
0.660	0.56	-0.31	0.45	0.63
0.661	0.56	-0.31	0.44	0.63
0.663	0.58	-0.30	0.44	0.61
0.665	0.59	-0.29	0.43	0.62
0.667	0.60	-0.27	0.41	0.63
0.668	0.61	-0.26	0.41	0.62
0.670	0.60	-0.26	0.41	0.63
0.672	0.61	-0.25	0.38	0.64
0.674	0.63	-0.24	0.36	0.64
0.676	0.64	-0.24	0.37	0.64
0.677	0.64	-0.25	0.36	0.63
0.679	0.68	-0.22	0.36	0.60
0.681	0.71	-0.20	0.34	0.59
0.683	0.71	-0.23	0.33	0.58
0.684	0.72	-0.14	0.32	0.60
0.686	0.77	-0.16	0.32	0.53
0.688	0.78	-0.18	0.33	0.50
0.690	0.77	-0.17	0.35	0.51
0.691	0.77	-0.14	0.35	0.51

0.693	0.79	-0.14	0.33	0.50
0.695	0.80	-0.15	0.32	0.48
0.697	0.82	-0.15	0.33	0.44
0.699	0.77	-0.20	0.35	0.49
0.700	0.75	-0.22	0.32	0.53
0.702	0.72	-0.22	0.36	0.56
0.704	0.69	-0.23	0.41	0.55
0.706	0.69	-0.28	0.40	0.53
0.707	0.70	-0.32	0.40	0.50
0.709	0.69	-0.36	0.42	0.47
0.711	0.68	-0.40	0.42	0.45
0.713	0.71	-0.39	0.41	0.41
0.715	0.78	-0.35	0.36	0.38
0.716	0.80	-0.33	0.33	0.39
0.718	0.77	-0.33	0.34	0.42
0.720	0.78	-0.32	0.35	0.41
0.722	0.80	-0.32	0.33	0.38
0.723	0.82	-0.30	0.30	0.37
0.725	0.83	-0.28	0.29	0.40
0.727	0.82	-0.25	0.35	0.38
0.729	0.83	-0.22	0.34	0.38
0.730	0.85	-0.17	0.31	0.39
0.732	0.84	-0.15	0.30	0.42
0.734	0.83	-0.15	0.30	0.44
0.736	0.82	-0.15	0.30	0.46
0.738	0.82	-0.14	0.28	0.48
0.739	0.82	-0.12	0.27	0.48
0.741	0.75	-0.12	0.34	0.55
0.743	0.79	-0.11	0.31	0.52
0.745	0.82	-0.11	0.27	0.49
0.746	0.82	-0.09	0.27	0.49
0.748	0.80	-0.07	0.25	0.54
0.750	0.79	-0.08	0.20	0.57
0.752	0.79	-0.10	0.22	0.56
0.754	0.78	-0.05	0.26	0.56
0.755	0.77	-0.02	0.22	0.60
0.757	0.79	-0.04	0.21	0.57
0.759	0.81	-0.10	0.19	0.54
0.761	0.80	-0.13	0.19	0.56
0.762	0.76	-0.12	0.20	0.60
0.764	0.75	-0.11	0.18	0.63

0.766	0.76	-0.10	0.17	0.62
0.768	0.76	-0.08	0.20	0.62
0.770	0.77	-0.03	0.22	0.60
0.771	0.77	-0.08	0.16	0.61
0.773	0.78	-0.13	0.14	0.60
0.775	0.78	-0.13	0.16	0.60
0.777	0.76	-0.13	0.16	0.62
0.778	0.75	-0.17	0.17	0.62
0.780	0.75	-0.17	0.18	0.61
0.782	0.77	-0.11	0.17	0.61
0.784	0.74	-0.17	0.18	0.62
0.785	0.72	-0.24	0.16	0.63
0.787	0.70	-0.27	0.14	0.65
0.789	0.69	-0.24	0.16	0.67
0.791	0.69	-0.21	0.20	0.67
0.793	0.71	-0.20	0.20	0.65
0.794	0.71	-0.22	0.20	0.64
0.796	0.69	-0.21	0.21	0.66
0.798	0.68	-0.22	0.25	0.66
0.800	0.68	-0.24	0.23	0.65
0.801	0.68	-0.23	0.21	0.66
0.803	0.68	-0.21	0.21	0.67
0.805	0.67	-0.21	0.24	0.67
0.807	0.67	-0.18	0.26	0.67
0.809	0.67	-0.18	0.25	0.67
0.810	0.66	-0.20	0.23	0.68
0.812	0.65	-0.16	0.25	0.70
0.814	0.64	-0.14	0.27	0.70
0.816	0.64	-0.13	0.30	0.70
0.817	0.65	-0.11	0.29	0.70
0.819	0.65	-0.09	0.28	0.70
0.821	0.64	-0.05	0.30	0.70
0.823	0.64	-0.02	0.31	0.70
0.824	0.65	-0.00	0.30	0.70
0.826	0.65	0.04	0.30	0.70
0.828	0.64	0.05	0.31	0.70
0.830	0.63	0.07	0.33	0.70
0.832	0.63	0.09	0.34	0.69
0.833	0.65	0.13	0.33	0.67
0.835	0.66	0.16	0.32	0.65
0.837	0.65	0.18	0.34	0.65

0.839	0.64	0.19	0.36	0.65
0.840	0.61	0.28	0.34	0.66
0.842	0.59	0.31	0.32	0.67
0.844	0.61	0.34	0.30	0.65
0.846	0.63	0.35	0.30	0.62
0.848	0.59	0.41	0.32	0.62
0.849	0.57	0.46	0.31	0.60
0.851	0.56	0.50	0.29	0.59
0.853	0.53	0.52	0.29	0.60
0.855	0.57	0.49	0.33	0.57
0.856	0.60	0.50	0.32	0.53
0.858	0.63	0.52	0.29	0.49
0.860	0.64	0.54	0.26	0.49
0.862	0.63	0.54	0.25	0.49
0.863	0.66	0.50	0.27	0.50
0.865	0.71	0.41	0.30	0.48
0.867	0.76	0.35	0.31	0.45
0.869	0.80	0.34	0.28	0.40
0.871	0.75	0.35	0.32	0.46
0.872	0.72	0.35	0.34	0.49
0.874	0.76	0.31	0.35	0.45
0.876	0.82	0.23	0.36	0.36
0.878	0.85	0.20	0.34	0.34
0.879	0.86	0.21	0.33	0.32
0.881	0.86	0.23	0.36	0.28
0.883	0.84	0.16	0.37	0.36
0.885	0.84	0.22	0.36	0.35
0.887	0.86	0.24	0.35	0.29
0.888	0.88	0.20	0.37	0.24
0.890	0.88	0.16	0.38	0.24
0.892	0.88	0.15	0.35	0.29
0.894	0.88	0.14	0.34	0.29
0.895	0.87	0.13	0.38	0.27
0.897	0.88	0.08	0.40	0.26
0.899	0.86	0.11	0.40	0.30
0.901	0.88	0.07	0.37	0.30
0.902	0.88	0.05	0.31	0.34
0.904	0.85	0.11	0.32	0.40
0.906	0.85	0.08	0.38	0.36
0.908	0.85	0.03	0.40	0.36
0.910	0.78	0.08	0.37	0.50

0.911	0.79	0.13	0.30	0.52
0.913	0.81	0.03	0.34	0.48
0.915	0.78	-0.05	0.34	0.52
0.917	0.73	-0.05	0.31	0.61
0.918	0.70	-0.06	0.34	0.63
0.920	0.66	-0.09	0.36	0.65
0.922	0.66	-0.05	0.38	0.64
0.924	0.69	0.04	0.39	0.60
0.926	0.68	0.06	0.27	0.68
0.927	0.63	-0.06	0.24	0.73
0.929	0.62	-0.12	0.26	0.73
0.931	0.57	-0.12	0.29	0.76
0.933	0.46	-0.12	0.28	0.83
0.934	0.46	-0.17	0.33	0.81
0.936	0.54	-0.22	0.39	0.71
0.938	0.55	-0.25	0.37	0.71
0.940	0.56	-0.29	0.36	0.69
0.941	0.66	-0.28	0.38	0.59
0.943	0.72	-0.32	0.44	0.44
0.945	0.76	-0.33	0.44	0.36
0.947	0.78	-0.28	0.44	0.35
0.949	0.78	-0.26	0.46	0.33
0.950	0.76	-0.30	0.51	0.26
0.952	0.79	-0.33	0.48	0.20
0.954	0.81	-0.26	0.50	0.12
0.956	0.83	-0.25	0.47	0.14
0.957	0.85	-0.18	0.46	0.17
0.959	0.86	-0.11	0.46	0.17
0.961	0.86	-0.10	0.48	0.16
0.963	0.83	-0.05	0.54	0.11
0.965	0.79	-0.00	0.61	0.05
0.966	0.76	-0.04	0.64	-0.02
0.968	0.79	-0.06	0.61	-0.04
0.970	0.81	-0.04	0.57	-0.09
0.972	0.78	0.05	0.61	-0.10
0.973	0.75	0.16	0.64	-0.06
0.975	0.77	0.21	0.60	-0.02
0.977	0.75	0.11	0.65	-0.04
0.979	0.67	0.08	0.73	-0.11
0.980	0.66	0.19	0.71	-0.18
0.982	0.62	0.39	0.64	-0.23

0.984	0.68	0.67	0.28	-0.01
0.986	0.40	0.85	-0.33	0.07
0.988	0.23	0.83	-0.51	-0.02
0.989	0.17	0.76	-0.62	-0.06
0.991	0.01	0.64	-0.75	-0.16
0.993	-0.17	0.64	-0.72	-0.20
0.995	-0.23	0.71	-0.64	-0.14
0.996	-0.22	0.68	-0.65	-0.26
0.998	-0.28	0.76	-0.48	-0.32
1.000	-0.41	0.78	-0.27	-0.39
0.000	-0.06	-0.20	0.77	0.61
0.002	-0.16	-0.08	0.87	0.45
0.004	-0.36	0.18	0.69	0.60
0.005	-0.43	0.21	0.61	0.63
0.007	0.06	-0.91	0.30	0.28
0.009	0.45	-0.54	0.57	0.42
0.011	0.19	0.84	0.00	-0.51
0.012	-0.01	0.62	0.63	-0.46
0.014	0.70	0.12	0.67	-0.20
0.016	0.89	-0.30	0.28	0.19
0.018	0.79	-0.32	0.48	0.19
0.020	0.71	-0.27	0.65	0.06
0.021	0.76	-0.29	0.57	-0.08
0.023	0.64	-0.38	0.61	0.25
0.025	0.29	-0.21	0.92	0.16
0.027	0.47	-0.58	0.51	0.42
0.028	0.48	-0.39	0.39	0.68
0.030	0.78	-0.09	0.20	0.58
0.032	0.80	0.30	0.12	0.50
0.034	0.73	0.67	-0.08	0.11
0.035	0.37	0.89	-0.14	-0.25
0.037	0.20	0.90	-0.18	-0.34
0.039	0.27	0.95	-0.02	-0.17
0.041	0.43	0.87	0.04	0.22
0.043	0.49	0.54	0.39	0.56
0.044	0.34	0.87	-0.05	-0.35
0.046	0.47	0.50	-0.33	-0.65
0.048	-0.19	0.57	-0.28	-0.74
0.050	-0.08	0.37	-0.32	-0.87
0.051	-0.11	0.54	-0.34	-0.77
0.053	-0.18	0.49	-0.34	-0.78

0.055	-0.23	0.53	-0.32	-0.75
0.057	-0.28	0.60	-0.29	-0.69
0.059	-0.34	0.63	-0.25	-0.66
0.060	-0.37	0.64	-0.21	-0.64
0.062	-0.39	0.66	-0.18	-0.62
0.064	-0.42	0.65	-0.18	-0.60
0.066	-0.44	0.66	-0.15	-0.59
0.067	-0.46	0.66	-0.15	-0.57
0.069	-0.50	0.65	-0.16	-0.56
0.071	-0.52	0.63	-0.15	-0.56
0.073	-0.52	0.64	-0.14	-0.55
0.074	-0.52	0.63	-0.13	-0.57
0.076	-0.51	0.62	-0.14	-0.58
0.078	-0.50	0.63	-0.15	-0.58
0.080	-0.50	0.63	-0.14	-0.57
0.082	-0.47	0.64	-0.13	-0.60
0.083	-0.42	0.67	-0.13	-0.60
0.085	-0.37	0.72	-0.13	-0.58
0.087	-0.28	0.78	-0.14	-0.54
0.089	-0.23	0.81	-0.15	-0.52
0.090	-0.21	0.81	-0.17	-0.52
0.092	-0.16	0.85	-0.16	-0.48
0.094	-0.07	0.87	-0.17	-0.45
0.096	-0.13	0.87	-0.17	-0.45
0.098	-0.19	0.88	-0.15	-0.41
0.099	-0.24	0.89	-0.12	-0.37
0.101	-0.29	0.89	-0.10	-0.34
0.103	-0.35	0.87	-0.09	-0.33
0.105	-0.36	0.88	-0.07	-0.31
0.106	-0.36	0.88	-0.06	-0.31
0.108	-0.35	0.89	-0.02	-0.30
0.110	-0.34	0.89	-0.00	-0.31
0.112	-0.33	0.89	0.01	-0.32
0.113	-0.33	0.88	0.01	-0.34
0.115	-0.32	0.88	-0.01	-0.35
0.117	-0.29	0.88	-0.06	-0.38
0.119	-0.23	0.88	-0.11	-0.41
0.121	-0.19	0.87	-0.15	-0.44
0.122	-0.20	0.85	-0.17	-0.45
0.124	-0.24	0.86	-0.14	-0.42
0.126	-0.31	0.86	-0.10	-0.40

0.128	-0.35	0.85	-0.09	-0.38
0.129	-0.37	0.85	-0.08	-0.37
0.131	-0.37	0.85	-0.08	-0.36
0.133	-0.38	0.85	-0.09	-0.36
0.135	-0.39	0.84	-0.08	-0.37
0.137	-0.41	0.83	-0.09	-0.36
0.138	-0.44	0.80	-0.10	-0.40
0.140	-0.46	0.77	-0.11	-0.43
0.142	-0.47	0.75	-0.10	-0.45
0.144	-0.48	0.73	-0.11	-0.47
0.145	-0.49	0.70	-0.11	-0.51
0.147	-0.51	0.67	-0.11	-0.53
0.149	-0.51	0.68	-0.11	-0.52
0.151	-0.48	0.70	-0.12	-0.51
0.152	-0.49	0.69	-0.12	-0.52
0.154	-0.50	0.68	-0.10	-0.52
0.156	-0.50	0.68	-0.09	-0.53
0.158	-0.49	0.69	-0.08	-0.53
0.160	-0.47	0.69	-0.09	-0.54
0.161	-0.46	0.71	-0.11	-0.52
0.163	-0.45	0.73	-0.12	-0.50
0.165	-0.49	0.71	-0.06	-0.50
0.167	-0.49	0.73	-0.07	-0.48
0.168	-0.47	0.76	-0.08	-0.45
0.170	-0.45	0.79	-0.07	-0.42
0.172	-0.42	0.81	-0.05	-0.40
0.174	-0.41	0.82	-0.06	-0.39
0.176	-0.40	0.83	-0.07	-0.38
0.177	-0.39	0.83	-0.08	-0.40
0.179	-0.42	0.84	-0.08	-0.33
0.181	-0.41	0.85	-0.07	-0.33
0.183	-0.39	0.86	-0.06	-0.32
0.184	-0.41	0.86	-0.05	-0.32
0.186	-0.42	0.85	-0.04	-0.32
0.188	-0.45	0.83	-0.05	-0.31
0.190	-0.47	0.83	-0.08	-0.29
0.191	-0.48	0.81	-0.11	-0.31
0.193	-0.50	0.80	-0.07	-0.33
0.195	-0.47	0.82	-0.06	-0.32
0.197	-0.45	0.83	-0.06	-0.32
0.199	-0.43	0.84	-0.07	-0.33

0.200	-0.40	0.85	-0.07	-0.33
0.202	-0.39	0.86	-0.06	-0.33
0.204	-0.39	0.86	-0.05	-0.33
0.206	-0.38	0.86	-0.04	-0.34
0.207	-0.32	0.89	-0.05	-0.31
0.209	-0.30	0.91	-0.03	-0.28
0.211	-0.27	0.93	-0.01	-0.24
0.213	-0.23	0.95	-0.01	-0.22
0.215	-0.18	0.96	-0.05	-0.22
0.216	-0.10	0.96	-0.10	-0.22
0.218	-0.04	0.96	-0.15	-0.23
0.220	0.01	0.96	-0.18	-0.22
0.222	0.07	0.97	-0.17	-0.17
0.223	0.08	0.96	-0.17	-0.20
0.225	0.06	0.97	-0.15	-0.20
0.227	0.03	0.97	-0.13	-0.20
0.229	-0.01	0.98	-0.13	-0.15
0.230	-0.02	0.98	-0.11	-0.14
0.232	-0.02	0.99	-0.06	-0.13
0.234	-0.02	0.99	-0.01	-0.12
0.236	-0.03	0.99	0.11	-0.09
0.238	-0.03	0.99	0.11	-0.09
0.239	-0.03	0.99	0.15	-0.07
0.241	-0.00	0.96	0.27	-0.06
0.243	0.04	0.93	0.37	-0.05
0.245	0.03	0.92	0.38	-0.02
0.246	0.02	0.94	0.34	0.01
0.248	-0.03	0.96	0.28	0.05
0.250	0.02	0.97	0.22	0.10
0.252	-0.03	0.98	0.17	0.12
0.254	-0.05	0.98	0.12	0.12
0.255	-0.05	0.99	0.08	0.12
0.257	-0.05	0.99	0.05	0.11
0.259	-0.04	0.99	0.05	0.09
0.261	-0.03	1.00	0.05	0.06
0.262	-0.02	1.00	0.02	0.03
0.264	-0.01	1.00	0.05	0.05
0.266	-0.01	1.00	0.03	0.03
0.268	-0.02	1.00	0.01	0.03
0.270	0.00	1.00	-0.01	0.04
0.271	0.05	1.00	-0.03	0.04

0.273	0.07	1.00	-0.03	0.03
0.275	0.06	1.00	-0.02	0.01
0.277	0.03	1.00	-0.01	0.01
0.278	0.08	0.99	-0.05	0.06
0.280	0.08	0.99	-0.04	0.06
0.282	0.09	0.99	-0.04	0.05
0.284	0.12	0.99	-0.04	0.05
0.285	0.14	0.99	-0.06	0.04
0.287	0.14	0.99	-0.05	0.05
0.289	0.12	0.99	-0.04	0.05
0.291	0.13	0.99	-0.05	0.04
0.293	0.16	0.99	-0.07	0.01
0.294	0.15	0.99	-0.06	0.02
0.296	0.14	0.99	-0.05	0.05
0.298	0.14	0.99	-0.05	0.05
0.300	0.14	0.99	-0.06	0.03
0.301	0.15	0.99	-0.04	0.02
0.303	0.16	0.99	-0.03	0.02
0.305	0.15	0.99	-0.03	0.02
0.307	0.16	0.99	-0.01	0.03
0.309	0.15	0.99	-0.02	0.01
0.310	0.16	0.99	-0.03	0.01
0.312	0.17	0.98	-0.02	0.01
0.314	0.16	0.99	0.02	0.01
0.316	0.16	0.99	0.06	0.01
0.317	0.17	0.98	0.05	0.02
0.319	0.17	0.99	0.02	0.02
0.321	0.15	0.99	0.04	0.03
0.323	0.14	0.99	0.06	0.04
0.324	0.12	0.99	0.07	0.03
0.326	0.11	0.99	0.07	0.02
0.328	0.12	0.99	0.07	0.04
0.330	0.13	0.99	0.07	0.05
0.332	0.12	0.99	0.07	0.03
0.333	0.10	0.99	0.07	0.02
0.335	0.14	0.99	0.07	0.04
0.337	0.13	0.99	0.08	0.05
0.339	0.13	0.98	0.11	0.05
0.340	0.12	0.98	0.14	0.06
0.342	0.13	0.98	0.15	0.06
0.344	0.13	0.98	0.15	0.05

0.346	0.15	0.98	0.14	0.05
0.348	0.16	0.97	0.15	0.05
0.349	0.13	0.98	0.12	0.08
0.351	0.15	0.98	0.12	0.07
0.353	0.17	0.98	0.12	0.05
0.355	0.17	0.98	0.09	0.04
0.356	0.16	0.98	0.06	0.03
0.358	0.16	0.98	0.06	0.04
0.360	0.16	0.98	0.08	0.04
0.362	0.16	0.98	0.10	0.04
0.363	0.17	0.98	0.11	0.07
0.365	0.18	0.97	0.13	0.08
0.367	0.19	0.97	0.15	0.08
0.369	0.19	0.97	0.16	0.08
0.371	0.18	0.96	0.18	0.08
0.372	0.16	0.96	0.20	0.09
0.374	0.11	0.96	0.23	0.10
0.376	0.09	0.95	0.27	0.09
0.378	0.01	0.95	0.32	0.04
0.379	-0.05	0.94	0.35	0.02
0.381	-0.13	0.93	0.34	-0.00
0.383	-0.18	0.94	0.29	-0.03
0.385	-0.20	0.95	0.23	-0.06
0.387	-0.21	0.96	0.15	-0.10
0.388	-0.21	0.96	0.04	-0.16
0.390	-0.19	0.97	-0.03	-0.15
0.392	-0.13	0.97	-0.10	-0.19
0.394	-0.13	0.96	-0.14	-0.21
0.395	-0.10	0.95	-0.17	-0.23
0.397	-0.08	0.94	-0.17	-0.28
0.399	-0.11	0.92	-0.17	-0.34
0.401	-0.15	0.91	-0.16	-0.36
0.402	-0.21	0.89	-0.14	-0.37
0.404	-0.26	0.89	-0.12	-0.36
0.406	-0.32	0.85	-0.08	-0.41
0.408	-0.36	0.83	-0.08	-0.42
0.410	-0.40	0.80	-0.08	-0.44
0.411	-0.42	0.77	-0.07	-0.47
0.413	-0.45	0.74	-0.05	-0.50
0.415	-0.47	0.69	-0.03	-0.55
0.417	-0.48	0.64	-0.02	-0.60

0.418	-0.49	0.55	-0.01	-0.68
0.420	-0.47	0.51	-0.02	-0.72
0.422	-0.43	0.51	0.02	-0.74
0.424	-0.33	0.64	0.07	-0.69
0.426	-0.18	0.78	0.15	-0.58
0.427	-0.02	0.88	0.21	-0.42
0.429	0.08	0.94	0.25	-0.21
0.431	0.16	0.96	0.25	-0.05
0.433	0.16	0.96	0.22	0.07
0.434	0.19	0.94	0.24	0.17
0.436	0.15	0.94	0.20	0.22
0.438	0.13	0.94	0.18	0.24
0.440	0.10	0.96	0.16	0.23
0.441	0.07	0.96	0.13	0.22
0.443	0.07	0.96	0.12	0.23
0.445	0.07	0.96	0.11	0.23
0.447	0.08	0.97	0.11	0.22
0.449	0.08	0.97	0.10	0.21
0.450	0.09	0.97	0.11	0.19
0.452	0.09	0.97	0.13	0.16
0.454	0.10	0.98	0.14	0.13
0.456	0.11	0.98	0.14	0.08
0.457	0.13	0.98	0.13	0.03
0.459	0.15	0.98	0.11	-0.01
0.461	0.18	0.98	0.09	-0.03
0.463	0.22	0.97	0.06	-0.07
0.465	0.28	0.96	0.03	-0.10
0.466	0.37	0.92	-0.02	-0.12
0.468	0.47	0.87	-0.07	-0.14
0.470	0.55	0.82	-0.12	-0.14
0.472	0.63	0.74	-0.19	-0.13
0.473	0.69	0.67	-0.26	-0.11
0.475	0.73	0.60	-0.30	-0.10
0.477	0.76	0.55	-0.33	-0.07
0.479	0.79	0.50	-0.36	-0.08
0.480	0.80	0.44	-0.39	-0.10
0.482	0.80	0.42	-0.40	-0.12
0.484	0.80	0.43	-0.41	-0.14
0.486	0.78	0.45	-0.41	-0.16
0.488	0.76	0.47	-0.43	-0.16
0.489	0.71	0.55	-0.42	-0.14

0.491	0.61	0.65	-0.44	-0.10
0.493	0.50	0.75	-0.44	-0.04
0.495	0.37	0.82	-0.43	0.01
0.496	0.27	0.86	-0.44	0.06
0.498	0.23	0.85	-0.46	0.10
0.500	0.20	0.83	-0.51	0.13
0.502	0.16	0.79	-0.56	0.19
0.504	0.12	0.74	-0.60	0.26
0.505	0.09	0.67	-0.65	0.35
0.507	0.06	0.57	-0.70	0.43
0.509	0.02	0.42	-0.75	0.52
0.511	0.01	0.26	-0.79	0.56
0.512	0.02	0.10	-0.81	0.57
0.514	0.05	-0.05	-0.83	0.56
0.516	0.15	-0.18	-0.82	0.53
0.518	0.29	-0.34	-0.77	0.45
0.520	0.46	-0.44	-0.70	0.33
0.521	0.58	-0.46	-0.64	0.20
0.523	0.69	-0.39	-0.61	0.07
0.525	0.72	-0.31	-0.62	0.01
0.527	0.73	-0.19	-0.65	-0.06
0.528	0.73	0.00	-0.68	-0.09
0.530	0.70	0.20	-0.68	-0.10
0.532	0.63	0.42	-0.65	-0.07
0.534	0.58	0.56	-0.59	-0.03
0.535	0.50	0.68	-0.53	-0.01
0.537	0.43	0.78	-0.46	0.02
0.539	0.38	0.84	-0.39	0.04
0.541	0.36	0.87	-0.34	0.08
0.543	0.33	0.88	-0.30	0.14
0.544	0.29	0.89	-0.25	0.25
0.546	0.25	0.87	-0.20	0.38
0.548	0.18	0.82	-0.09	0.54
0.550	0.13	0.76	-0.02	0.64
0.551	0.06	0.69	0.08	0.72
0.553	0.03	0.63	0.17	0.76
0.555	-0.01	0.61	0.24	0.76
0.557	-0.05	0.62	0.27	0.73
0.559	-0.08	0.62	0.29	0.72
0.560	-0.10	0.64	0.30	0.70
0.562	-0.11	0.68	0.27	0.67

0.564	-0.12	0.72	0.25	0.64
0.566	-0.11	0.74	0.24	0.61
0.567	-0.10	0.77	0.25	0.58
0.569	-0.09	0.79	0.27	0.55
0.571	-0.09	0.80	0.29	0.52
0.573	-0.08	0.81	0.32	0.48
0.574	-0.07	0.83	0.36	0.42
0.576	-0.07	0.85	0.40	0.35
0.578	-0.08	0.87	0.40	0.28
0.580	-0.08	0.88	0.42	0.21
0.582	-0.07	0.88	0.45	0.13
0.583	-0.06	0.88	0.48	0.05
0.585	-0.04	0.85	0.53	-0.05
0.587	-0.02	0.82	0.55	-0.15
0.589	0.00	0.78	0.58	-0.23
0.590	-0.00	0.79	0.53	-0.30
0.592	0.03	0.77	0.51	-0.39
0.594	0.06	0.76	0.47	-0.45
0.596	0.09	0.77	0.43	-0.46
0.598	0.12	0.79	0.39	-0.46
0.599	0.16	0.79	0.34	-0.49
0.601	0.21	0.78	0.30	-0.52
0.603	0.24	0.77	0.27	-0.52
0.605	0.33	0.74	0.23	-0.53
0.606	0.39	0.73	0.22	-0.52
0.608	0.46	0.71	0.21	-0.48
0.610	0.53	0.70	0.20	-0.43
0.612	0.60	0.70	0.18	-0.34
0.613	0.67	0.70	0.16	-0.20
0.615	0.72	0.68	0.14	-0.03
0.617	0.75	0.64	0.11	0.11
0.619	0.78	0.56	0.09	0.25
0.621	0.80	0.53	0.09	0.26
0.622	0.81	0.52	0.08	0.26
0.624	0.82	0.51	0.07	0.26
0.626	0.82	0.52	0.07	0.24
0.628	0.81	0.55	0.07	0.18
0.629	0.80	0.58	0.07	0.12
0.631	0.81	0.58	0.05	0.06
0.633	0.78	0.62	0.05	0.01
0.635	0.76	0.64	0.03	-0.01

0.637	0.73	0.68	0.02	-0.04
0.638	0.69	0.72	0.02	-0.05
0.640	0.67	0.74	0.01	-0.06
0.642	0.66	0.75	-0.02	-0.07
0.644	0.65	0.76	-0.07	-0.04
0.645	0.68	0.73	-0.10	-0.03
0.647	0.69	0.72	-0.08	-0.00
0.649	0.68	0.72	-0.13	0.03
0.651	0.67	0.72	-0.19	0.08
0.652	0.66	0.70	-0.22	0.14
0.654	0.65	0.68	-0.25	0.23
0.656	0.63	0.65	-0.28	0.32
0.658	0.62	0.60	-0.30	0.40
0.660	0.62	0.56	-0.31	0.44
0.661	0.59	0.50	-0.31	0.55
0.663	0.58	0.49	-0.30	0.57
0.665	0.58	0.45	-0.30	0.61
0.667	0.61	0.40	-0.30	0.62
0.668	0.61	0.37	-0.29	0.63
0.670	0.62	0.35	-0.25	0.65
0.672	0.63	0.35	-0.19	0.66
0.674	0.65	0.36	-0.13	0.66
0.676	0.68	0.35	-0.11	0.63
0.677	0.69	0.34	-0.10	0.64
0.679	0.71	0.32	-0.08	0.63
0.681	0.74	0.31	-0.04	0.59
0.683	0.76	0.30	-0.03	0.58
0.684	0.75	0.31	-0.03	0.59
0.686	0.75	0.32	-0.02	0.57
0.688	0.78	0.32	-0.00	0.54
0.690	0.80	0.32	-0.01	0.51
0.691	0.78	0.35	-0.03	0.52
0.693	0.78	0.40	-0.04	0.48
0.695	0.79	0.43	-0.06	0.43
0.697	0.80	0.45	-0.09	0.38
0.699	0.80	0.48	-0.13	0.34
0.700	0.79	0.51	-0.19	0.28
0.702	0.78	0.54	-0.25	0.20
0.704	0.76	0.56	-0.32	0.06
0.706	0.73	0.57	-0.37	-0.01
0.707	0.68	0.61	-0.40	-0.02

0.709	0.64	0.64	-0.43	-0.02
0.711	0.64	0.62	-0.46	-0.05
0.713	0.66	0.57	-0.48	-0.10
0.715	0.69	0.50	-0.50	-0.15
0.716	0.69	0.46	-0.53	-0.18
0.718	0.71	0.47	-0.48	-0.20
0.720	0.75	0.45	-0.45	-0.17
0.722	0.79	0.43	-0.41	-0.13
0.723	0.82	0.40	-0.40	-0.10
0.725	0.84	0.38	-0.37	-0.09
0.727	0.87	0.37	-0.30	-0.06
0.729	0.91	0.35	-0.22	-0.01
0.730	0.93	0.31	-0.16	0.05
0.732	0.91	0.34	-0.18	0.13
0.734	0.90	0.37	-0.15	0.17
0.736	0.89	0.39	-0.12	0.23
0.738	0.88	0.40	-0.11	0.26
0.739	0.87	0.42	-0.10	0.26
0.741	0.85	0.42	-0.08	0.30
0.743	0.83	0.44	-0.07	0.34
0.745	0.80	0.48	-0.04	0.36
0.746	0.81	0.48	0.01	0.33
0.748	0.80	0.49	0.01	0.36
0.750	0.78	0.48	0.01	0.40
0.752	0.78	0.48	0.02	0.40
0.754	0.78	0.48	0.02	0.39
0.755	0.78	0.50	0.04	0.37
0.757	0.77	0.53	0.05	0.37
0.759	0.77	0.52	0.03	0.36
0.761	0.77	0.55	0.05	0.33
0.762	0.78	0.54	0.06	0.32
0.764	0.79	0.53	0.05	0.30
0.766	0.80	0.52	0.05	0.29
0.768	0.80	0.52	0.05	0.28
0.770	0.82	0.50	0.04	0.28
0.771	0.83	0.48	0.03	0.27
0.773	0.83	0.48	0.04	0.28
0.775	0.84	0.47	0.03	0.26
0.777	0.85	0.45	0.02	0.26
0.778	0.86	0.44	0.01	0.25
0.780	0.87	0.44	-0.00	0.24

0.782	0.87	0.43	-0.01	0.25
0.784	0.87	0.42	-0.01	0.27
0.785	0.87	0.42	-0.02	0.27
0.787	0.88	0.40	-0.04	0.26
0.789	0.89	0.39	-0.03	0.25
0.791	0.88	0.39	-0.01	0.26
0.793	0.88	0.40	-0.01	0.27
0.794	0.89	0.38	-0.02	0.25
0.796	0.90	0.36	-0.03	0.25
0.798	0.89	0.37	-0.02	0.26
0.800	0.88	0.38	-0.00	0.27
0.801	0.90	0.34	-0.01	0.27
0.803	0.92	0.32	-0.02	0.23
0.805	0.92	0.30	-0.00	0.25
0.807	0.92	0.28	0.02	0.27
0.809	0.92	0.26	0.03	0.28
0.810	0.92	0.26	0.04	0.29
0.812	0.92	0.25	0.05	0.31
0.814	0.92	0.23	0.05	0.32
0.816	0.92	0.22	0.07	0.33
0.817	0.92	0.16	0.10	0.35
0.819	0.92	0.12	0.09	0.37
0.821	0.91	0.11	0.10	0.38
0.823	0.91	0.09	0.12	0.40
0.824	0.90	0.07	0.14	0.41
0.826	0.90	0.06	0.15	0.41
0.828	0.90	0.03	0.15	0.42
0.830	0.88	0.02	0.14	0.45
0.832	0.89	0.02	0.13	0.44
0.833	0.89	0.04	0.13	0.42
0.835	0.90	0.05	0.13	0.41
0.837	0.90	0.05	0.13	0.41
0.839	0.91	0.06	0.12	0.40
0.840	0.92	0.07	0.11	0.38
0.842	0.92	0.08	0.11	0.36
0.844	0.93	0.07	0.11	0.35
0.846	0.92	0.07	0.11	0.36
0.848	0.92	0.11	0.12	0.35
0.849	0.92	0.13	0.12	0.34
0.851	0.93	0.12	0.13	0.33
0.853	0.93	0.13	0.13	0.32

0.855	0.93	0.16	0.13	0.32
0.856	0.93	0.17	0.13	0.31
0.858	0.92	0.18	0.14	0.30
0.860	0.93	0.20	0.14	0.29
0.862	0.93	0.20	0.14	0.29
0.863	0.93	0.21	0.14	0.28
0.865	0.92	0.22	0.15	0.27
0.867	0.92	0.23	0.16	0.26
0.869	0.93	0.21	0.17	0.25
0.871	0.94	0.18	0.18	0.23
0.872	0.95	0.17	0.18	0.21
0.874	0.94	0.17	0.19	0.20
0.876	0.95	0.17	0.19	0.18
0.878	0.95	0.18	0.19	0.16
0.879	0.95	0.18	0.20	0.14
0.881	0.95	0.18	0.20	0.13
0.883	0.95	0.18	0.21	0.12
0.885	0.95	0.19	0.21	0.10
0.887	0.96	0.17	0.21	0.08
0.888	0.96	0.17	0.21	0.05
0.890	0.96	0.17	0.23	0.04
0.892	0.95	0.16	0.25	0.03
0.894	0.95	0.16	0.26	0.02
0.895	0.95	0.16	0.27	-0.00
0.897	0.95	0.15	0.27	-0.02
0.899	0.95	0.15	0.28	-0.03
0.901	0.95	0.16	0.28	-0.04
0.902	0.95	0.15	0.27	-0.02
0.904	0.95	0.15	0.27	0.00
0.906	0.95	0.14	0.26	0.03
0.908	0.95	0.15	0.26	0.04
0.910	0.95	0.16	0.26	0.06
0.911	0.95	0.17	0.26	0.08
0.913	0.94	0.17	0.25	0.12
0.915	0.95	0.15	0.24	0.15
0.917	0.94	0.16	0.23	0.20
0.918	0.93	0.16	0.23	0.23
0.920	0.92	0.16	0.24	0.26
0.922	0.91	0.16	0.24	0.30
0.924	0.91	0.13	0.22	0.33
0.926	0.90	0.12	0.20	0.38

0.927	0.88	0.11	0.19	0.41
0.929	0.88	0.10	0.17	0.43
0.931	0.86	0.08	0.15	0.48
0.933	0.83	0.04	0.15	0.54
0.934	0.82	-0.01	0.14	0.56
0.936	0.82	-0.08	0.18	0.54
0.938	0.80	-0.16	0.24	0.53
0.940	0.78	-0.24	0.27	0.50
0.941	0.79	-0.29	0.31	0.44
0.943	0.78	-0.37	0.34	0.36
0.945	0.79	-0.39	0.39	0.28
0.947	0.80	-0.42	0.37	0.23
0.949	0.82	-0.42	0.35	0.18
0.950	0.84	-0.41	0.33	0.14
0.952	0.86	-0.38	0.33	0.12
0.954	0.87	-0.34	0.33	0.11
0.956	0.89	-0.29	0.34	0.11
0.957	0.90	-0.24	0.34	0.11
0.959	0.92	-0.20	0.33	0.09
0.961	0.92	-0.16	0.35	0.08
0.963	0.93	-0.10	0.36	0.07
0.965	0.92	-0.04	0.37	0.06
0.966	0.92	0.03	0.39	0.05
0.968	0.91	0.08	0.40	0.02
0.970	0.91	0.12	0.40	-0.03
0.972	0.90	0.17	0.41	-0.05
0.973	0.88	0.20	0.42	-0.10
0.975	0.87	0.22	0.43	-0.12
0.977	0.86	0.24	0.43	-0.14
0.979	0.85	0.30	0.41	-0.17
0.980	0.83	0.36	0.38	-0.19
0.982	0.80	0.42	0.36	-0.22
0.984	0.78	0.47	0.35	-0.23
0.986	0.72	0.56	0.32	-0.24
0.988	0.63	0.71	0.21	-0.24
0.989	0.53	0.82	0.04	-0.22
0.991	0.37	0.88	-0.22	-0.18
0.993	0.23	0.85	-0.44	-0.19
0.995	0.15	0.81	-0.52	-0.24
0.996	0.06	0.80	-0.53	-0.28
0.998	-0.09	0.77	-0.58	-0.25

1.000	-0.19	0.70	-0.65	-0.21
0.000	-0.08	-0.37	0.80	-0.46
0.002	-0.20	-0.66	-0.07	0.72
0.004	0.46	-0.48	0.74	-0.04
0.005	-0.37	-0.52	0.75	-0.16
0.007	-0.39	-0.66	0.22	-0.61
0.009	0.17	-0.65	-0.21	-0.71
0.011	0.02	-0.88	-0.16	-0.46
0.012	0.04	-0.94	0.34	0.03
0.014	-0.13	-0.37	0.79	0.47
0.016	-0.21	-0.22	0.78	0.55
0.018	-0.23	-0.14	0.87	0.41
0.020	-0.19	-0.11	0.98	0.03
0.021	0.16	-0.87	0.39	0.24
0.023	0.31	-0.89	0.27	-0.21
0.025	0.52	-0.67	0.25	-0.47
0.027	0.42	-0.51	0.75	-0.05
0.028	0.42	-0.63	0.59	0.28
0.030	0.49	-0.66	0.43	0.36
0.032	0.54	-0.71	0.32	0.32
0.034	0.61	-0.72	0.22	0.25
0.035	0.71	-0.56	0.19	0.38
0.037	0.88	-0.14	0.04	0.44
0.039	0.80	0.35	-0.29	0.39
0.041	0.60	0.58	-0.51	0.21
0.043	0.38	0.74	-0.56	-0.04
0.044	0.01	0.83	-0.55	-0.07
0.046	-0.09	0.76	-0.64	-0.08
0.048	-0.02	0.67	-0.67	-0.32
0.050	-0.13	0.87	-0.41	-0.24
0.051	0.07	0.75	-0.60	-0.25
0.053	0.55	-0.05	-0.83	0.04
0.055	0.57	0.26	-0.73	0.26
0.057	0.27	0.64	-0.69	-0.21
0.059	0.10	0.30	-0.84	-0.43
0.060	0.00	-0.26	-0.74	-0.62
0.062	-0.02	-0.46	-0.77	-0.45
0.064	-0.48	-0.59	-0.56	-0.33
0.066	-0.30	-0.28	-0.89	-0.18
0.067	0.18	0.04	-0.98	0.06
0.069	0.19	0.38	-0.90	0.09

0.071	-0.09	0.38	-0.88	-0.28
0.073	-0.83	0.03	-0.43	-0.35
0.074	-0.87	-0.30	-0.24	-0.31
0.076	-0.84	-0.37	-0.34	-0.23
0.078	-0.93	-0.09	-0.29	-0.21
0.080	-0.96	-0.01	-0.20	-0.18
0.082	-0.99	0.10	-0.05	-0.06
0.083	-0.99	0.09	0.12	-0.02
0.085	-0.98	-0.09	0.17	-0.09
0.087	-0.97	-0.14	0.17	-0.12
0.089	-0.96	-0.13	0.14	-0.19
0.090	-0.94	-0.14	0.04	-0.30
0.092	-0.64	-0.58	-0.50	-0.02
0.094	-0.34	-0.55	-0.76	-0.07
0.096	-0.07	-0.45	-0.89	-0.01
0.098	0.03	-0.52	-0.85	0.12
0.099	0.08	-0.68	-0.71	0.15
0.101	0.22	-0.76	-0.57	0.22
0.103	0.42	-0.67	-0.58	0.21
0.105	0.73	-0.54	-0.39	0.15
0.106	0.55	-0.78	-0.13	0.26
0.108	0.55	-0.82	-0.10	0.07
0.110	0.36	-0.88	-0.21	-0.21
0.112	0.27	-0.88	-0.33	-0.18
0.113	0.27	-0.84	-0.45	-0.11
0.115	0.24	-0.88	-0.42	-0.04
0.117	0.28	-0.88	-0.35	-0.15
0.119	0.42	-0.85	-0.20	-0.25
0.121	0.28	-0.92	-0.23	0.13
0.122	0.43	-0.87	-0.20	0.14
0.124	0.58	-0.80	-0.07	0.10
0.126	0.79	-0.53	0.19	-0.25
0.128	0.49	-0.47	0.22	-0.70
0.129	0.22	-0.90	0.04	-0.37
0.131	-0.01	-1.00	0.00	0.06
0.133	-0.06	-0.97	0.03	0.23
0.135	-0.15	-0.83	0.28	0.46
0.137	0.05	-0.91	0.17	0.39
0.138	0.21	-0.94	0.04	0.28
0.140	0.33	-0.93	0.03	0.15
0.142	0.37	-0.92	0.08	0.14

0.144	0.33	-0.89	0.16	0.27
0.145	0.25	-0.76	0.38	0.46
0.147	0.20	-0.63	0.56	0.50
0.149	0.28	-0.70	0.50	0.42
0.151	0.28	-0.76	0.45	0.37
0.152	0.29	-0.82	0.38	0.30
0.154	0.25	-0.84	0.38	0.29
0.156	0.23	-0.77	0.47	0.37
0.158	0.30	-0.72	0.46	0.41
0.160	0.42	-0.66	0.45	0.44
0.161	0.51	-0.61	0.41	0.45
0.163	0.31	-0.86	0.34	0.22
0.165	0.37	-0.80	0.37	0.27
0.167	0.43	-0.70	0.48	0.31
0.168	0.47	-0.61	0.57	0.30
0.170	0.42	-0.62	0.56	0.35
0.172	0.46	-0.63	0.52	0.34
0.174	0.54	-0.61	0.50	0.29
0.176	0.59	-0.58	0.52	0.20
0.177	0.66	-0.61	0.34	0.25
0.179	0.54	-0.73	0.37	0.21
0.181	0.43	-0.82	0.34	0.19
0.183	0.44	-0.81	0.29	0.27
0.184	0.60	-0.67	0.26	0.36
0.186	0.75	-0.48	0.27	0.37
0.188	0.74	-0.48	0.36	0.31
0.190	0.68	-0.55	0.42	0.26
0.191	0.49	-0.60	0.49	0.41
0.193	0.57	-0.48	0.46	0.48
0.195	0.73	-0.30	0.45	0.42
0.197	0.88	-0.30	0.35	0.15
0.199	0.84	-0.38	0.37	0.09
0.200	0.78	-0.50	0.32	0.17
0.202	0.72	-0.48	0.36	0.35
0.204	0.72	-0.33	0.40	0.46
0.206	0.62	-0.29	0.66	0.29
0.207	0.75	-0.23	0.61	0.12
0.209	0.84	-0.18	0.51	0.04
0.211	0.87	-0.16	0.45	0.11
0.213	0.86	-0.14	0.31	0.38
0.215	0.80	-0.08	0.34	0.48

0.216	0.83	0.03	0.41	0.38
0.218	0.86	0.10	0.47	0.18
0.220	0.82	0.21	0.50	0.21
0.222	0.82	0.21	0.46	0.27
0.223	0.81	0.25	0.44	0.29
0.225	0.72	0.16	0.56	0.39
0.227	0.57	0.08	0.71	0.41
0.229	0.43	0.05	0.77	0.47
0.230	0.47	0.24	0.66	0.53
0.232	0.54	0.40	0.44	0.59
0.234	0.48	0.41	0.43	0.65
0.236	0.63	0.15	0.24	0.72
0.238	0.64	-0.09	0.18	0.74
0.239	0.51	-0.25	0.24	0.79
0.241	0.33	-0.38	0.27	0.82
0.243	0.29	-0.29	0.44	0.80
0.245	0.44	-0.10	0.55	0.70
0.246	0.60	-0.02	0.51	0.61
0.248	0.59	0.14	0.65	0.46
0.250	0.62	0.19	0.65	0.39
0.252	0.57	0.19	0.69	0.41
0.254	0.49	0.13	0.70	0.50
0.255	0.47	0.02	0.71	0.53
0.257	0.55	0.12	0.70	0.44
0.259	0.55	0.30	0.64	0.45
0.261	0.47	0.32	0.60	0.56
0.262	0.52	0.19	0.81	0.18
0.264	0.54	0.15	0.78	0.27
0.266	0.55	0.15	0.73	0.39
0.268	0.53	0.13	0.73	0.41
0.270	0.53	0.07	0.76	0.35
0.271	0.53	-0.01	0.81	0.26
0.273	0.55	0.00	0.82	0.17
0.275	0.54	0.07	0.83	0.12
0.277	0.52	0.03	0.79	0.32
0.278	0.45	0.14	0.78	0.41
0.280	0.39	0.34	0.74	0.44
0.282	0.42	0.47	0.66	0.40
0.284	0.47	0.34	0.72	0.38
0.285	0.44	0.14	0.80	0.38
0.287	0.44	0.08	0.81	0.37

0.289	0.49	0.09	0.81	0.32
0.291	0.53	-0.04	0.77	0.35
0.293	0.52	0.12	0.74	0.41
0.294	0.39	0.19	0.77	0.46
0.296	0.24	0.12	0.85	0.45
0.298	0.28	0.18	0.86	0.38
0.300	0.41	0.30	0.81	0.30
0.301	0.48	0.26	0.81	0.21
0.303	0.38	-0.02	0.91	0.16
0.305	0.29	-0.17	0.93	0.15
0.307	0.27	0.03	0.92	0.27
0.309	0.26	0.18	0.87	0.38
0.310	0.24	0.16	0.88	0.39
0.312	0.22	0.03	0.91	0.35
0.314	0.31	-0.05	0.90	0.31
0.316	0.35	0.05	0.87	0.35
0.317	0.42	0.19	0.80	0.39
0.319	0.53	0.21	0.76	0.29
0.321	0.54	0.10	0.79	0.28
0.323	0.52	0.06	0.82	0.23
0.324	0.46	0.11	0.85	0.25
0.326	0.42	0.17	0.87	0.22
0.328	0.36	0.16	0.90	0.19
0.330	0.34	0.04	0.93	0.12
0.332	0.33	-0.11	0.93	0.10
0.333	0.42	0.29	0.79	0.34
0.335	0.48	0.21	0.80	0.30
0.337	0.56	0.14	0.78	0.25
0.339	0.56	0.16	0.77	0.26
0.340	0.59	0.18	0.75	0.22
0.342	0.60	0.24	0.72	0.24
0.344	0.64	0.19	0.70	0.26
0.346	0.65	0.09	0.72	0.24
0.348	0.54	0.18	0.80	0.22
0.349	0.44	0.22	0.84	0.23
0.351	0.44	0.22	0.84	0.23
0.353	0.44	0.19	0.84	0.25
0.355	0.57	0.13	0.76	0.28
0.356	0.56	0.14	0.76	0.30
0.358	0.53	0.16	0.76	0.32
0.360	0.43	0.25	0.81	0.32

0.362	0.44	0.43	0.75	0.26
0.363	0.42	0.39	0.75	0.33
0.365	0.44	0.33	0.74	0.40
0.367	0.46	0.31	0.72	0.42
0.369	0.43	0.40	0.68	0.44
0.371	0.50	0.46	0.66	0.33
0.372	0.52	0.48	0.66	0.26
0.374	0.55	0.44	0.70	0.14
0.376	0.43	0.45	0.68	0.40
0.378	0.46	0.47	0.62	0.43
0.379	0.48	0.50	0.57	0.45
0.381	0.51	0.48	0.61	0.37
0.383	0.54	0.36	0.67	0.36
0.385	0.66	0.21	0.60	0.41
0.387	0.69	0.19	0.45	0.54
0.388	0.67	0.23	0.35	0.61
0.390	0.69	0.30	0.34	0.56
0.392	0.70	0.21	0.38	0.56
0.394	0.72	0.13	0.39	0.56
0.395	0.75	0.11	0.42	0.51
0.397	0.74	0.18	0.40	0.50
0.399	0.72	0.18	0.45	0.49
0.401	0.69	0.11	0.48	0.53
0.402	0.67	0.03	0.53	0.52
0.404	0.74	-0.11	0.53	0.40
0.406	0.78	-0.04	0.50	0.37
0.408	0.80	0.04	0.51	0.31
0.410	0.77	0.04	0.57	0.29
0.411	0.76	-0.01	0.58	0.29
0.413	0.78	-0.03	0.56	0.30
0.415	0.85	-0.01	0.45	0.29
0.417	0.89	0.03	0.36	0.28
0.418	0.89	0.02	0.34	0.31
0.420	0.88	0.11	0.35	0.30
0.422	0.88	0.13	0.33	0.32
0.424	0.85	0.09	0.32	0.40
0.426	0.89	0.06	0.21	0.39
0.427	0.91	0.09	0.24	0.33
0.429	0.91	0.15	0.25	0.31
0.431	0.89	0.15	0.30	0.32
0.433	0.69	0.15	0.62	0.34

0.434	0.71	0.15	0.61	0.32
0.436	0.66	0.13	0.64	0.38
0.438	0.68	0.23	0.56	0.41
0.440	0.73	0.21	0.51	0.40
0.441	0.80	0.21	0.45	0.33
0.443	0.88	0.04	0.36	0.32
0.445	0.93	0.00	0.28	0.23
0.447	0.93	0.09	0.24	0.28
0.449	0.89	0.00	0.27	0.37
0.450	0.83	-0.07	0.31	0.46
0.452	0.74	-0.08	0.38	0.55
0.454	0.78	-0.07	0.41	0.47
0.456	0.84	0.18	0.36	0.35
0.457	0.90	0.31	0.24	0.18
0.459	0.90	0.27	0.26	0.21
0.461	0.89	0.15	0.29	0.33
0.463	0.85	0.07	0.41	0.31
0.465	0.82	0.18	0.45	0.31
0.466	0.82	0.23	0.45	0.26
0.468	0.83	0.22	0.41	0.31
0.470	0.80	0.23	0.39	0.40
0.472	0.75	0.22	0.40	0.48
0.473	0.73	0.16	0.41	0.52
0.475	0.63	0.37	0.37	0.57
0.477	0.58	0.53	0.31	0.54
0.479	0.46	0.68	0.23	0.52
0.480	0.37	0.75	0.19	0.51
0.482	0.31	0.78	0.15	0.52
0.484	0.29	0.79	0.18	0.52
0.486	0.28	0.78	0.20	0.52
0.488	0.28	0.79	0.22	0.51
0.489	0.22	0.78	0.27	0.52
0.491	0.27	0.78	0.25	0.51
0.493	0.37	0.73	0.24	0.52
0.495	0.45	0.65	0.28	0.55
0.496	0.51	0.56	0.36	0.55
0.498	0.61	0.43	0.40	0.54
0.500	0.68	0.31	0.34	0.57
0.502	0.73	0.23	0.26	0.59
0.504	0.71	0.12	0.20	0.66
0.505	0.67	0.00	0.16	0.73

0.507	0.53	-0.14	0.09	0.83
0.509	0.41	-0.21	0.01	0.89
0.511	0.38	-0.22	-0.08	0.89
0.512	0.36	-0.21	-0.05	0.91
0.514	0.36	-0.20	-0.01	0.91
0.516	0.38	-0.19	-0.00	0.90
0.518	0.44	-0.19	-0.01	0.88
0.520	0.54	-0.18	0.06	0.82
0.521	0.63	-0.18	0.13	0.75
0.523	0.68	-0.19	0.16	0.69
0.525	0.76	-0.20	0.15	0.60
0.527	0.86	-0.18	0.13	0.46
0.528	0.92	-0.13	0.10	0.36
0.530	0.93	-0.09	0.07	0.36
0.532	0.92	-0.05	-0.02	0.38
0.534	0.91	-0.00	-0.09	0.40
0.535	0.86	0.02	-0.19	0.48
0.537	0.76	0.03	-0.28	0.59
0.539	0.66	0.04	-0.33	0.67
0.541	0.57	0.04	-0.37	0.73
0.543	0.49	0.03	-0.37	0.79
0.544	0.43	0.04	-0.37	0.82
0.546	0.37	0.05	-0.39	0.84
0.548	0.39	0.06	-0.38	0.84
0.550	0.45	0.09	-0.40	0.80
0.551	0.53	0.10	-0.43	0.72
0.553	0.60	0.09	-0.46	0.64
0.555	0.68	0.08	-0.51	0.52
0.557	0.76	0.05	-0.52	0.40
0.559	0.81	0.02	-0.49	0.33
0.560	0.85	-0.05	-0.43	0.29
0.562	0.90	-0.09	-0.34	0.26
0.564	0.93	-0.13	-0.24	0.25
0.566	0.94	-0.16	-0.12	0.27
0.567	0.94	-0.18	-0.04	0.28
0.569	0.94	-0.16	-0.04	0.30
0.571	0.93	-0.13	-0.07	0.34
0.573	0.92	-0.11	-0.07	0.37
0.574	0.92	-0.10	-0.08	0.38
0.576	0.91	-0.05	-0.10	0.39
0.578	0.90	0.01	-0.10	0.41

0.580	0.89	0.03	-0.10	0.43
0.582	0.88	0.04	-0.12	0.46
0.583	0.87	0.04	-0.13	0.48
0.585	0.85	0.04	-0.15	0.50
0.587	0.83	0.08	-0.18	0.52
0.589	0.86	0.09	-0.18	0.46
0.590	0.87	0.06	-0.15	0.46
0.592	0.90	0.01	-0.08	0.43
0.594	0.91	-0.09	-0.01	0.40
0.596	0.91	-0.18	0.05	0.38
0.598	0.88	-0.25	0.09	0.40
0.599	0.85	-0.26	0.13	0.44
0.601	0.83	-0.25	0.16	0.48
0.603	0.81	-0.18	0.20	0.53
0.605	0.79	-0.11	0.24	0.56
0.606	0.77	-0.03	0.26	0.58
0.608	0.77	0.03	0.28	0.58
0.610	0.77	0.06	0.31	0.55
0.612	0.78	0.09	0.33	0.52
0.613	0.80	0.11	0.36	0.46
0.615	0.82	0.12	0.38	0.41
0.617	0.86	0.11	0.38	0.32
0.619	0.88	0.11	0.39	0.26
0.621	0.89	0.11	0.40	0.18
0.622	0.90	0.07	0.41	0.14
0.624	0.90	0.02	0.42	0.11
0.626	0.89	-0.01	0.44	0.11
0.628	0.90	-0.04	0.42	0.14
0.629	0.90	-0.08	0.38	0.20
0.631	0.89	-0.15	0.33	0.28
0.633	0.87	-0.20	0.28	0.36
0.635	0.84	-0.26	0.17	0.44
0.637	0.80	-0.30	0.08	0.51
0.638	0.76	-0.32	0.03	0.57
0.640	0.71	-0.35	0.01	0.61
0.642	0.68	-0.37	0.01	0.63
0.644	0.65	-0.40	0.01	0.65
0.645	0.64	-0.40	0.01	0.65
0.647	0.66	-0.42	0.03	0.62
0.649	0.66	-0.45	0.04	0.60
0.651	0.65	-0.45	0.05	0.61

0.652	0.64	-0.46	0.06	0.61
0.654	0.65	-0.46	0.09	0.60
0.656	0.64	-0.48	0.15	0.58
0.658	0.64	-0.49	0.20	0.56
0.660	0.69	-0.50	0.20	0.49
0.661	0.71	-0.49	0.24	0.44
0.663	0.73	-0.49	0.27	0.38
0.665	0.77	-0.49	0.27	0.30
0.667	0.82	-0.48	0.25	0.20
0.668	0.85	-0.46	0.25	0.10
0.670	0.86	-0.45	0.25	0.03
0.672	0.86	-0.44	0.23	0.02
0.674	0.87	-0.45	0.20	0.01
0.676	0.87	-0.45	0.21	0.04
0.677	0.86	-0.46	0.20	0.03
0.679	0.86	-0.49	0.16	0.02
0.681	0.85	-0.51	0.13	0.05
0.683	0.85	-0.51	0.13	0.07
0.684	0.84	-0.52	0.14	0.09
0.686	0.83	-0.53	0.15	0.11
0.688	0.84	-0.52	0.12	0.12
0.690	0.83	-0.53	0.11	0.13
0.691	0.83	-0.52	0.10	0.15
0.693	0.83	-0.52	0.11	0.19
0.695	0.81	-0.52	0.10	0.25
0.697	0.79	-0.53	0.08	0.30
0.699	0.78	-0.52	0.06	0.35
0.700	0.76	-0.51	0.04	0.39
0.702	0.73	-0.49	0.03	0.48
0.704	0.70	-0.47	0.06	0.54
0.706	0.66	-0.45	0.06	0.60
0.707	0.66	-0.42	0.04	0.62
0.709	0.66	-0.40	0.05	0.63
0.711	0.66	-0.39	0.06	0.64
0.713	0.64	-0.39	0.09	0.66
0.715	0.63	-0.37	0.13	0.67
0.716	0.63	-0.34	0.17	0.68
0.718	0.68	-0.37	0.19	0.61
0.720	0.70	-0.42	0.21	0.53
0.722	0.71	-0.50	0.23	0.44
0.723	0.72	-0.57	0.30	0.27

0.725	0.68	-0.64	0.36	0.02
0.727	0.50	-0.72	0.35	-0.34
0.729	0.27	-0.64	0.27	-0.66
0.730	0.07	-0.41	0.10	-0.91
0.732	-0.01	-0.38	0.08	-0.92
0.734	-0.14	-0.28	0.01	-0.95
0.736	-0.21	-0.23	-0.09	-0.94
0.738	-0.25	-0.21	-0.18	-0.93
0.739	-0.29	-0.19	-0.23	-0.91
0.741	-0.33	-0.19	-0.27	-0.88
0.743	-0.33	-0.21	-0.30	-0.87
0.745	-0.32	-0.29	-0.30	-0.85
0.746	-0.25	-0.36	-0.34	-0.83
0.748	-0.19	-0.44	-0.37	-0.80
0.750	-0.13	-0.52	-0.38	-0.76
0.752	-0.04	-0.63	-0.34	-0.70
0.754	0.03	-0.74	-0.27	-0.62
0.755	0.11	-0.81	-0.19	-0.55
0.757	0.22	-0.85	-0.14	-0.47
0.759	0.38	-0.86	-0.06	-0.33
0.761	0.48	-0.84	0.02	-0.23
0.762	0.57	-0.81	0.10	-0.12
0.764	0.62	-0.77	0.16	-0.01
0.766	0.68	-0.69	0.24	0.09
0.768	0.73	-0.61	0.27	0.14
0.770	0.77	-0.53	0.31	0.19
0.771	0.78	-0.46	0.37	0.23
0.773	0.78	-0.40	0.41	0.24
0.775	0.80	-0.33	0.42	0.27
0.777	0.81	-0.26	0.41	0.32
0.778	0.82	-0.19	0.41	0.35
0.780	0.84	-0.12	0.39	0.35
0.782	0.87	-0.06	0.37	0.31
0.784	0.89	-0.02	0.34	0.31
0.785	0.88	-0.01	0.32	0.34
0.787	0.85	0.09	0.36	0.37
0.789	0.85	0.11	0.36	0.37
0.791	0.85	0.14	0.34	0.38
0.793	0.85	0.17	0.31	0.39
0.794	0.86	0.20	0.28	0.39
0.796	0.85	0.22	0.29	0.37

0.798	0.88	0.22	0.28	0.33
0.800	0.89	0.23	0.26	0.28
0.801	0.91	0.21	0.28	0.22
0.803	0.91	0.20	0.32	0.18
0.805	0.91	0.18	0.37	0.10
0.807	0.92	0.16	0.36	0.08
0.809	0.95	0.13	0.28	0.09
0.810	0.97	0.07	0.23	0.05
0.812	0.96	-0.04	0.27	-0.01
0.814	0.96	-0.11	0.27	-0.02
0.816	0.96	-0.20	0.15	-0.13
0.817	0.96	-0.15	0.08	-0.21
0.819	0.96	-0.12	0.04	-0.26
0.821	0.96	-0.17	0.02	-0.23
0.823	0.94	-0.28	-0.00	-0.18
0.824	0.92	-0.36	-0.02	-0.15
0.826	0.93	-0.35	-0.06	-0.13
0.828	0.94	-0.30	-0.07	-0.12
0.830	0.96	-0.27	-0.06	-0.06
0.832	0.96	-0.27	-0.03	-0.05
0.833	0.96	-0.27	-0.05	-0.05
0.835	0.96	-0.26	-0.07	-0.03
0.837	0.96	-0.26	-0.07	-0.00
0.839	0.96	-0.26	-0.07	0.03
0.840	0.97	-0.25	-0.06	0.05
0.842	0.97	-0.24	-0.07	0.07
0.844	0.97	-0.22	-0.09	0.11
0.846	0.97	-0.20	-0.07	0.12
0.848	0.97	-0.19	-0.06	0.12
0.849	0.97	-0.21	-0.07	0.11
0.851	0.96	-0.22	-0.09	0.11
0.853	0.96	-0.23	-0.11	0.12
0.855	0.96	-0.21	-0.11	0.13
0.856	0.96	-0.20	-0.11	0.13
0.858	0.96	-0.23	-0.11	0.13
0.860	0.95	-0.24	-0.13	0.14
0.862	0.95	-0.24	-0.15	0.14
0.863	0.95	-0.25	-0.15	0.15
0.865	0.95	-0.24	-0.13	0.16
0.867	0.94	-0.26	-0.13	0.17
0.869	0.93	-0.29	-0.13	0.17

0.871	0.93	-0.31	-0.15	0.16
0.872	0.92	-0.32	-0.10	0.19
0.874	0.92	-0.33	-0.10	0.18
0.876	0.91	-0.35	-0.10	0.19
0.878	0.90	-0.36	-0.08	0.22
0.879	0.90	-0.35	-0.07	0.25
0.881	0.90	-0.35	-0.05	0.27
0.883	0.89	-0.36	-0.05	0.29
0.885	0.87	-0.38	-0.04	0.31
0.887	0.86	-0.37	-0.01	0.35
0.888	0.84	-0.39	0.01	0.37
0.890	0.83	-0.39	0.04	0.40
0.892	0.82	-0.40	0.07	0.41
0.894	0.81	-0.41	0.10	0.42
0.895	0.80	-0.41	0.11	0.42
0.897	0.79	-0.40	0.13	0.45
0.899	0.76	-0.39	0.18	0.48
0.901	0.75	-0.38	0.23	0.50
0.902	0.74	-0.38	0.25	0.50
0.904	0.72	-0.37	0.27	0.52
0.906	0.71	-0.37	0.28	0.53
0.908	0.70	-0.35	0.31	0.54
0.910	0.67	-0.35	0.34	0.55
0.911	0.65	-0.34	0.38	0.56
0.913	0.64	-0.33	0.42	0.55
0.915	0.64	-0.35	0.44	0.52
0.917	0.63	-0.34	0.49	0.50
0.918	0.63	-0.35	0.52	0.46
0.920	0.64	-0.34	0.53	0.44
0.922	0.63	-0.34	0.56	0.42
0.924	0.64	-0.34	0.59	0.37
0.926	0.65	-0.34	0.61	0.31
0.927	0.64	-0.33	0.64	0.27
0.929	0.63	-0.32	0.65	0.26
0.931	0.64	-0.31	0.66	0.22
0.933	0.67	-0.33	0.64	0.15
0.934	0.69	-0.35	0.63	0.11
0.936	0.66	-0.36	0.65	0.11
0.938	0.68	-0.34	0.63	0.12
0.940	0.74	-0.30	0.59	0.14
0.941	0.74	-0.22	0.63	0.09
0.943	0.71	-0.34	0.62	0.00
0.945	0.72	-0.32	0.62	0.06

0.947	0.72	-0.32	0.59	0.18
0.949	0.71	-0.35	0.56	0.26
0.950	0.68	-0.41	0.56	0.23
0.952	0.68	-0.41	0.59	0.16
0.954	0.77	-0.32	0.55	0.06
0.956	0.81	-0.23	0.53	0.04
0.957	0.83	-0.28	0.47	-0.03
0.959	0.88	-0.37	0.30	0.00
0.961	0.92	-0.39	0.04	0.04
0.963	0.94	-0.31	-0.10	0.06
0.965	0.97	-0.21	0.10	0.01
0.966	0.91	-0.23	0.33	0.11
0.968	0.80	-0.33	0.42	0.27
0.970	0.70	-0.40	0.37	0.47
0.972	0.76	-0.46	0.34	0.32
0.973	0.72	-0.38	0.47	0.34
0.975	0.63	-0.28	0.66	0.31
0.977	0.54	-0.31	0.75	0.23
0.979	0.48	-0.43	0.75	0.17
0.980	0.45	-0.51	0.72	0.13
0.982	0.50	-0.51	0.69	0.12
0.984	0.50	-0.51	0.69	0.13

Table S2. Factor Loadings obtained from PCA with varimax rotation analysis based on data from TLC fingerprints of separated iridoid compounds on Silicagel and RP-18 chromatographic plates using detection with two reagents – anisaldehydeand Erlich reagent respectively. (The highest contribution for values >0.9)

Variable/ Rf	Factor Loadings (Varimax normalized, data from chromatograms on RP-C18) Extraction: Principal components (Marked loadings are >.700000)			
	Factor 1	Factor 2	Factor 3	Factor 4
0.000	-0.54	-0.07	-0.71	-0.44
0.002	-0.50	-0.03	-0.79	0.36
0.004	-0.11	0.09	-0.90	0.41
0.005	0.23	0.38	-0.89	-0.10
0.007	0.16	0.25	-0.85	-0.43
0.009	0.04	0.02	-0.89	-0.45
0.011	-0.39	-0.69	-0.61	0.09
0.012	-0.62	-0.61	-0.49	-0.04
0.014	-0.68	-0.57	-0.43	-0.13
0.016	-0.24	-0.69	-0.52	-0.44
0.018	-0.23	-0.55	-0.72	-0.37
0.019	-0.49	-0.67	-0.54	-0.15
0.021	-0.20	-0.96	-0.21	-0.02
0.023	0.14	-0.99	0.06	-0.02
0.025	0.10	-0.98	0.10	0.16
0.026	0.37	-0.88	0.14	0.25

0.028	0.42	-0.78	0.23	0.39
0.030	0.28	-0.76	0.31	0.50
0.032	0.03	-0.79	0.29	0.54
0.033	-0.31	-0.84	0.05	0.45
0.035	-0.58	-0.64	-0.50	0.10
0.037	-0.51	-0.29	-0.79	-0.18
0.039	-0.47	-0.15	-0.86	-0.14
0.040	-0.44	-0.22	-0.86	-0.09
0.042	-0.46	-0.12	-0.88	-0.07
0.044	-0.46	-0.07	-0.88	-0.03
0.046	-0.34	-0.24	-0.91	0.01
0.047	-0.22	-0.36	-0.91	-0.02
0.049	-0.21	-0.42	-0.88	-0.07
0.051	-0.20	-0.50	-0.84	0.02
0.053	-0.13	-0.57	-0.75	-0.31
0.054	-0.16	-0.49	-0.80	-0.31

0.056	-0.20	-0.46	-0.78	-0.37
0.058	-0.33	-0.55	-0.68	-0.35
0.060	-0.28	-0.73	-0.54	-0.32
0.061	-0.26	-0.72	-0.55	-0.34
0.063	-0.22	-0.72	-0.53	-0.39
0.065	-0.11	-0.65	-0.53	-0.53
0.067	0.12	-0.61	-0.59	-0.51
0.068	0.14	-0.59	-0.72	-0.32
0.070	0.11	-0.59	-0.73	-0.33
0.072	0.02	-0.56	-0.62	-0.55
0.074	-0.12	-0.58	-0.63	-0.49
0.075	-0.11	-0.56	-0.81	-0.12
0.077	0.12	-0.64	-0.76	-0.03
0.079	0.45	-0.67	-0.57	-0.13
0.081	0.10	-0.57	-0.74	0.33
0.082	-0.42	-0.49	-0.75	0.15
0.084	-0.35	-0.56	-0.75	-0.00
0.086	0.08	-0.62	-0.77	-0.11
0.088	0.04	-0.68	-0.68	-0.27
0.089	-0.30	-0.81	-0.33	-0.39
0.091	-0.29	-0.85	-0.32	-0.30
0.093	0.15	-0.75	-0.50	-0.40
0.095	0.16	-0.91	-0.35	-0.17
0.096	0.12	-0.99	-0.09	-0.02
0.098	-0.13	-0.99	-0.01	-0.03
0.100	-0.15	-0.98	-0.03	-0.16
0.102	-0.04	-0.97	-0.03	-0.25
0.104	0.01	-0.90	-0.26	-0.35
0.105	0.06	-0.88	-0.38	-0.27
0.107	0.18	-0.97	-0.06	-0.13
0.109	-0.06	-0.92	0.11	-0.38
0.111	-0.04	-0.95	-0.11	-0.28
0.112	-0.08	-0.92	-0.36	-0.15
0.114	-0.09	-0.94	-0.33	-0.03
0.116	-0.07	-1.00	-0.05	0.04
0.118	-0.11	-0.98	0.14	0.10
0.119	-0.11	-0.97	0.18	0.15
0.121	-0.15	-0.96	0.14	0.19
0.123	-0.25	-0.94	0.23	0.04
0.125	-0.29	-0.90	0.29	-0.13
0.126	-0.28	-0.85	0.45	0.03

0.128	-0.36	-0.81	0.37	0.29
0.130	-0.48	-0.83	0.11	0.24
0.132	-0.52	-0.83	0.12	0.16
0.133	-0.50	-0.83	0.22	0.11
0.135	-0.53	-0.84	0.10	-0.08
0.137	-0.54	-0.83	-0.09	0.15
0.139	-0.41	-0.89	-0.15	0.12
0.140	-0.33	-0.89	-0.26	0.14
0.142	-0.43	-0.78	-0.37	0.26
0.144	-0.58	-0.57	-0.45	0.37
0.146	-0.61	-0.67	-0.42	0.11
0.147	-0.40	-0.84	-0.37	-0.05
0.149	-0.14	-0.88	-0.40	-0.19
0.151	-0.44	-0.22	-0.86	-0.16
0.153	-0.13	-0.53	-0.82	-0.16
0.154	0.05	-0.39	-0.92	0.02
0.156	-0.21	-0.18	-0.95	-0.11
0.158	-0.27	-0.20	-0.91	-0.26
0.160	0.01	-0.19	-0.98	0.08
0.161	0.22	0.00	-0.91	0.34
0.163	-0.14	-0.15	-0.97	-0.08
0.165	0.07	-0.50	-0.86	0.02
0.167	0.21	-0.38	-0.90	0.04
0.168	0.30	-0.17	-0.93	-0.14
0.170	0.28	-0.06	-0.89	-0.35
0.172	0.29	-0.30	-0.86	-0.31
0.174	0.31	-0.49	-0.81	0.01
0.175	0.33	-0.34	-0.86	0.20
0.177	0.20	-0.03	-0.95	0.24
0.179	0.01	-0.17	-0.97	0.17
0.181	0.05	-0.16	-0.91	0.37
0.182	0.12	-0.30	-0.89	0.31
0.184	0.13	-0.51	-0.85	0.03
0.186	0.12	-0.55	-0.82	-0.03
0.188	0.05	-0.41	-0.90	0.14
0.189	-0.05	-0.36	-0.92	0.12
0.191	-0.11	-0.38	-0.91	-0.12
0.193	0.26	-0.36	-0.88	0.17
0.195	-0.07	-0.43	-0.89	0.14
0.196	-0.28	-0.50	-0.82	0.08
0.198	-0.14	-0.43	-0.87	-0.17

0.200	0.05	-0.23	-0.90	-0.37
0.202	-0.15	-0.26	-0.95	-0.07
0.204	-0.28	-0.37	-0.88	0.14
0.205	-0.14	-0.42	-0.89	0.02
0.207	0.01	-0.42	-0.90	0.06
0.209	0.06	-0.32	-0.95	-0.03
0.211	0.03	-0.24	-0.97	-0.06
0.212	-0.05	-0.31	-0.95	0.02
0.214	-0.19	-0.30	-0.93	0.03
0.216	-0.38	-0.10	-0.92	0.00
0.218	-0.35	-0.03	-0.94	-0.00
0.219	-0.22	-0.16	-0.96	-0.00
0.221	-0.17	-0.27	-0.90	-0.29
0.223	-0.12	-0.41	-0.89	-0.13
0.225	-0.12	-0.35	-0.93	-0.01
0.226	-0.21	-0.19	-0.96	-0.06
0.228	-0.20	-0.34	-0.91	-0.14
0.230	-0.18	-0.45	-0.86	-0.18
0.232	-0.20	-0.38	-0.90	-0.06
0.233	-0.16	-0.34	-0.92	0.07
0.235	-0.13	-0.42	-0.89	-0.04
0.237	-0.30	-0.19	-0.94	-0.01
0.239	-0.29	-0.21	-0.93	0.06
0.240	-0.11	-0.34	-0.93	0.04
0.242	-0.16	-0.31	-0.93	-0.08
0.244	-0.28	-0.31	-0.90	-0.12
0.246	-0.22	-0.43	-0.87	-0.08
0.247	-0.06	-0.48	-0.88	-0.04
0.249	-0.10	-0.21	-0.95	-0.22
0.251	-0.10	0.05	-0.96	-0.25
0.253	-0.10	0.00	-0.99	-0.04
0.254	-0.12	-0.08	-0.98	0.17
0.256	-0.21	0.07	-0.97	0.11
0.258	-0.17	0.15	-0.97	-0.07
0.260	-0.10	0.03	-0.99	-0.02
0.261	-0.15	-0.01	-0.98	0.12
0.263	-0.13	-0.21	-0.96	0.10
0.265	-0.14	-0.04	-0.98	0.11
0.267	-0.20	0.11	-0.97	0.09
0.268	-0.19	0.11	-0.97	0.05
0.270	-0.19	0.06	-0.98	0.07

0.272	-0.17	0.05	-0.98	0.10
0.274	-0.21	0.03	-0.97	0.09
0.275	-0.24	-0.02	-0.97	0.01
0.277	-0.40	-0.12	-0.91	0.04
0.279	-0.27	0.09	-0.93	0.23
0.281	-0.13	0.02	-0.96	0.24
0.282	-0.13	-0.17	-0.97	0.11
0.284	-0.22	-0.09	-0.97	0.06
0.286	-0.05	-0.03	-0.99	0.15
0.288	0.03	-0.11	-0.98	0.17
0.289	-0.11	-0.13	-0.96	0.24
0.291	-0.02	-0.08	-0.98	0.19
0.293	-0.06	-0.09	-0.92	0.38
0.295	-0.08	0.13	-0.87	0.47
0.296	-0.10	0.22	-0.80	0.55
0.298	0.04	0.30	-0.84	0.44
0.300	0.28	0.26	-0.86	0.33
0.302	0.29	0.42	-0.77	0.38
0.304	0.15	0.50	-0.65	0.55
0.305	-0.03	0.39	-0.85	0.35
0.307	0.15	0.52	-0.81	0.23
0.309	0.28	0.57	-0.75	0.19
0.311	0.23	0.53	-0.80	0.18
0.312	0.17	0.33	-0.93	0.04
0.314	0.22	0.33	-0.92	0.03
0.316	0.26	0.34	-0.90	0.06
0.318	0.23	0.33	-0.92	0.02
0.319	0.07	0.10	-0.99	-0.02
0.321	-0.11	0.17	-0.98	0.05
0.323	-0.15	0.16	-0.97	0.15
0.325	-0.02	0.19	-0.98	0.07
0.326	0.04	0.10	-0.99	-0.06
0.328	0.03	0.06	-0.99	-0.11
0.330	0.06	0.01	-1.00	-0.02
0.332	0.05	0.04	-1.00	0.05
0.333	0.04	0.11	-0.99	0.10
0.335	0.16	0.16	-0.97	0.01
0.337	0.24	0.26	-0.93	-0.08
0.339	0.22	0.32	-0.92	-0.05
0.340	0.28	0.22	-0.93	0.02
0.342	0.34	0.18	-0.92	-0.06

0.344	0.12	0.27	-0.95	-0.10
0.346	-0.25	0.43	-0.86	-0.09
0.347	-0.24	0.24	-0.92	-0.22
0.349	-0.25	0.26	-0.93	-0.11
0.351	-0.06	0.28	-0.96	0.05
0.353	-0.03	0.32	-0.94	0.08
0.354	-0.24	0.44	-0.87	-0.04
0.356	-0.25	0.45	-0.86	-0.01
0.358	-0.13	0.44	-0.88	0.10
0.360	-0.24	0.37	-0.89	0.15
0.361	0.13	0.71	-0.69	0.02
0.363	-0.09	0.64	-0.75	-0.11
0.365	-0.29	0.63	-0.71	-0.14
0.367	-0.30	0.72	-0.63	-0.06
0.368	-0.28	0.76	-0.58	-0.06
0.370	-0.36	0.71	-0.59	-0.15
0.372	-0.23	0.78	-0.55	-0.16
0.374	-0.02	0.89	-0.44	-0.11
0.375	0.01	0.83	-0.35	-0.42
0.377	0.04	0.84	-0.45	-0.30
0.379	-0.04	0.81	-0.58	-0.05
0.381	-0.17	0.82	-0.54	0.01
0.382	-0.26	0.90	-0.34	-0.03
0.384	-0.17	0.88	-0.43	-0.09
0.386	-0.01	0.73	-0.67	-0.10
0.388	0.16	0.71	-0.69	0.02
0.389	0.12	0.43	-0.78	0.44
0.391	0.09	0.38	-0.60	0.70
0.393	-0.00	0.42	-0.39	0.82
0.395	-0.06	0.35	-0.38	0.85
0.396	0.04	0.24	-0.49	0.83
0.398	0.19	0.38	-0.45	0.78
0.400	0.19	0.36	-0.35	0.84
0.402	0.22	0.20	-0.39	0.87
0.404	0.48	0.32	-0.18	0.80
0.405	0.66	0.35	-0.19	0.64
0.407	0.80	0.30	-0.23	0.46
0.409	0.84	0.24	-0.22	0.44
0.411	0.87	0.20	-0.16	0.43
0.412	0.90	0.20	-0.04	0.39
0.414	0.91	0.23	-0.05	0.34

0.416	0.92	0.21	-0.10	0.30
0.418	0.94	0.24	-0.04	0.23
0.419	0.95	0.18	-0.04	0.23
0.421	0.97	0.14	-0.06	0.21
0.423	0.96	0.15	-0.09	0.23
0.425	0.95	0.19	-0.09	0.24
0.426	0.93	0.25	-0.09	0.26
0.428	0.90	0.33	-0.14	0.25
0.430	0.85	0.40	-0.25	0.23
0.432	0.69	0.57	-0.37	0.25
0.433	0.54	0.67	-0.39	0.32
0.435	0.45	0.76	-0.37	0.29
0.437	0.43	0.79	-0.40	0.19
0.439	0.38	0.81	-0.42	0.14
0.440	0.39	0.80	-0.43	0.16
0.442	0.40	0.82	-0.40	0.13
0.444	0.38	0.84	-0.37	0.06
0.446	0.35	0.84	-0.39	0.14
0.447	0.40	0.83	-0.37	0.14
0.449	0.46	0.80	-0.36	0.15
0.451	0.48	0.80	-0.34	0.13
0.453	0.46	0.81	-0.32	0.17
0.454	0.46	0.79	-0.30	0.25
0.456	0.50	0.75	-0.27	0.35
0.458	0.57	0.68	-0.24	0.39
0.460	0.45	0.75	-0.23	0.43
0.461	0.44	0.74	-0.22	0.46
0.463	0.46	0.67	-0.19	0.55
0.465	0.50	0.66	-0.17	0.53
0.467	0.50	0.70	-0.16	0.48
0.468	0.53	0.65	-0.02	0.53
0.470	0.51	0.60	-0.02	0.62
0.472	0.50	0.66	-0.09	0.56
0.474	0.40	0.67	-0.12	0.61
0.475	0.35	0.72	-0.01	0.59
0.477	0.30	0.74	0.09	0.60
0.479	0.23	0.66	0.13	0.70
0.481	0.12	0.71	0.10	0.69
0.482	0.11	0.78	0.09	0.61
0.484	0.08	0.85	0.04	0.53
0.486	0.08	0.85	0.03	0.52

0.488	0.10	0.79	0.20	0.57
0.489	-0.01	0.84	0.27	0.47
0.491	-0.07	0.88	0.23	0.41
0.493	-0.10	0.89	0.22	0.39
0.495	-0.05	0.85	0.29	0.44
0.496	-0.01	0.85	0.31	0.42
0.498	-0.04	0.87	0.35	0.34
0.500	-0.06	0.87	0.41	0.27
0.502	-0.05	0.87	0.42	0.23
0.504	-0.06	0.89	0.37	0.24
0.505	-0.06	0.89	0.37	0.25
0.507	-0.05	0.88	0.42	0.21
0.509	-0.04	0.89	0.44	0.14
0.511	-0.02	0.92	0.40	0.05
0.512	-0.01	0.93	0.37	0.00
0.514	-0.00	0.93	0.37	-0.01
0.516	-0.06	0.92	0.38	-0.02
0.518	-0.05	0.93	0.37	-0.03
0.519	-0.06	0.93	0.37	-0.04
0.521	-0.05	0.94	0.35	-0.04
0.523	-0.04	0.94	0.33	-0.03
0.525	-0.02	0.96	0.29	-0.02
0.526	0.01	0.97	0.24	-0.02
0.528	0.04	0.98	0.17	-0.01
0.530	-0.08	0.98	0.18	-0.02
0.532	-0.00	0.98	0.20	0.01
0.533	0.05	0.99	0.14	0.03
0.535	0.08	0.99	0.10	0.07
0.537	0.14	0.98	0.11	0.12
0.539	0.15	0.99	0.02	0.06
0.540	0.16	0.99	-0.00	-0.01
0.542	0.16	0.98	0.12	0.09
0.544	0.28	0.91	-0.08	0.29
0.546	0.24	0.94	-0.08	0.24
0.547	0.23	0.95	-0.01	0.19
0.549	0.27	0.95	0.08	0.14
0.551	0.33	0.93	0.01	0.16
0.553	0.33	0.93	-0.08	0.16
0.554	0.32	0.92	-0.09	0.23
0.556	0.33	0.90	-0.05	0.28
0.558	0.36	0.91	0.05	0.19

0.560	0.37	0.88	-0.11	0.27
0.561	0.42	0.85	-0.13	0.29
0.563	0.47	0.86	-0.00	0.19
0.565	0.46	0.87	0.08	0.15
0.567	0.38	0.90	0.02	0.20
0.568	0.37	0.89	0.02	0.26
0.570	0.40	0.87	0.09	0.28
0.572	0.44	0.88	0.05	0.16
0.574	0.42	0.89	0.02	0.16
0.575	0.40	0.91	0.02	0.14
0.577	0.37	0.92	0.04	0.08
0.579	0.39	0.92	0.03	0.00
0.581	0.40	0.92	-0.02	0.04
0.582	0.30	0.95	-0.01	0.12
0.584	0.21	0.96	0.06	0.19
0.586	0.15	0.97	-0.10	0.14
0.588	0.19	0.95	-0.16	0.20
0.589	0.24	0.93	-0.20	0.21
0.591	0.33	0.91	-0.18	0.16
0.593	0.42	0.88	-0.20	0.09
0.595	0.46	0.85	-0.23	0.12
0.596	0.35	0.90	-0.25	0.12
0.598	0.25	0.94	-0.20	0.07
0.600	0.24	0.93	-0.21	0.19
0.602	0.23	0.93	-0.24	0.16
0.604	0.21	0.93	-0.27	0.14
0.605	0.19	0.93	-0.30	0.11
0.607	0.19	0.92	-0.34	0.05
0.609	0.23	0.91	-0.34	0.00
0.611	0.27	0.90	-0.34	0.07
0.612	0.29	0.89	-0.31	0.17
0.614	0.32	0.91	-0.23	0.10
0.616	0.33	0.90	-0.22	0.17
0.618	0.34	0.91	-0.22	0.09
0.619	0.30	0.94	-0.18	0.06
0.621	0.23	0.96	-0.11	0.09
0.623	0.27	0.96	-0.10	-0.00
0.625	0.35	0.93	-0.08	-0.03
0.626	0.35	0.92	-0.07	0.16
0.628	0.09	0.99	-0.06	0.09
0.630	0.12	0.98	-0.10	0.14

0.632	0.17	0.98	-0.10	0.11
0.633	0.14	0.99	-0.00	0.01
0.635	0.11	0.99	0.06	0.08
0.637	0.12	0.97	0.04	0.20
0.639	0.17	0.97	0.01	0.17
0.640	0.26	0.97	0.02	0.01
0.642	0.37	0.93	0.04	-0.02
0.644	0.18	0.98	-0.00	0.12
0.646	0.11	0.97	0.04	0.21
0.647	0.21	0.94	0.16	0.20
0.649	0.25	0.94	0.17	0.15
0.651	0.12	0.98	0.06	0.12
0.653	0.09	0.97	0.04	0.20
0.654	0.13	0.95	0.11	0.27
0.656	0.27	0.96	0.05	0.08
0.658	0.16	0.98	-0.07	0.12
0.660	0.14	0.96	-0.05	0.24
0.661	0.11	0.94	-0.04	0.33
0.663	0.01	0.96	-0.13	0.26
0.665	-0.05	0.97	-0.04	0.22
0.667	0.10	0.96	0.03	0.24
0.668	0.31	0.89	-0.07	0.31
0.670	0.03	0.91	-0.11	0.41
0.672	-0.14	0.91	-0.12	0.37
0.674	-0.03	0.91	-0.08	0.40
0.675	0.06	0.88	-0.07	0.46
0.677	-0.04	0.87	-0.13	0.47
0.679	-0.07	0.88	-0.13	0.45
0.681	0.06	0.89	-0.09	0.44
0.682	0.06	0.93	-0.02	0.36
0.684	0.03	0.92	-0.16	0.34
0.686	-0.07	0.95	-0.12	0.27
0.688	0.01	0.96	0.01	0.29
0.689	0.07	0.91	0.20	0.35
0.691	-0.10	0.91	0.29	0.26
0.693	-0.16	0.94	0.25	0.18
0.695	-0.14	0.94	0.22	0.22
0.696	-0.24	0.90	0.23	0.28
0.698	-0.20	0.83	0.28	0.44
0.700	-0.22	0.87	0.31	0.32
0.702	-0.24	0.88	0.33	0.25

0.704	-0.24	0.87	0.35	0.26
0.705	-0.17	0.84	0.40	0.31
0.707	-0.14	0.84	0.44	0.29
0.709	-0.18	0.84	0.46	0.22
0.711	-0.26	0.84	0.44	0.17
0.712	-0.20	0.86	0.44	0.17
0.714	-0.18	0.84	0.49	0.15
0.716	-0.15	0.84	0.51	0.13
0.718	-0.12	0.85	0.50	0.11
0.719	-0.09	0.86	0.49	0.13
0.721	-0.11	0.86	0.48	0.15
0.723	-0.10	0.86	0.49	0.14
0.725	-0.07	0.85	0.51	0.12
0.726	-0.12	0.81	0.55	0.15
0.728	-0.09	0.84	0.52	0.12
0.730	-0.06	0.86	0.50	0.08
0.732	-0.06	0.86	0.49	0.10
0.733	-0.02	0.87	0.47	0.15
0.735	-0.01	0.89	0.42	0.17
0.737	-0.05	0.89	0.42	0.16
0.739	-0.14	0.86	0.47	0.17
0.740	0.00	0.88	0.43	0.20
0.742	-0.01	0.87	0.45	0.22
0.744	-0.05	0.86	0.43	0.25
0.746	-0.10	0.86	0.43	0.27
0.747	-0.04	0.85	0.43	0.29
0.749	0.03	0.87	0.39	0.31
0.751	0.03	0.88	0.37	0.29
0.753	-0.03	0.89	0.36	0.28
0.754	0.08	0.81	0.24	0.53
0.756	-0.05	0.84	0.21	0.51
0.758	-0.17	0.81	0.12	0.54
0.760	-0.14	0.78	0.09	0.60
0.761	-0.04	0.77	0.18	0.61
0.763	-0.05	0.72	0.32	0.61
0.765	-0.09	0.71	0.33	0.62
0.767	-0.12	0.72	0.20	0.66
0.768	-0.13	0.71	0.27	0.64
0.770	-0.13	0.74	0.36	0.55
0.772	-0.13	0.77	0.36	0.51
0.774	-0.10	0.79	0.31	0.51

0.775	-0.13	0.79	0.35	0.48
0.777	-0.16	0.77	0.41	0.46
0.779	-0.16	0.75	0.44	0.47
0.781	-0.10	0.74	0.42	0.52
0.782	-0.02	0.66	0.40	0.64
0.784	0.05	0.62	0.36	0.70
0.786	0.13	0.58	0.30	0.75
0.788	0.19	0.55	0.20	0.79
0.789	0.23	0.51	0.08	0.82
0.791	0.34	0.31	-0.05	0.88
0.793	0.48	0.19	-0.09	0.85
0.795	0.55	0.14	-0.07	0.82
0.796	0.56	0.21	-0.13	0.79
0.798	0.63	0.15	-0.10	0.75
0.800	0.69	0.09	-0.13	0.70
0.802	0.70	0.12	-0.18	0.68
0.804	0.69	0.16	-0.24	0.67
0.805	0.75	0.14	-0.24	0.59
0.807	0.79	0.07	-0.23	0.57
0.809	0.80	0.02	-0.21	0.57
0.811	0.85	0.08	-0.03	0.52
0.812	0.85	0.17	-0.10	0.49
0.814	0.81	0.18	-0.16	0.53
0.816	0.84	0.16	-0.12	0.51
0.818	0.86	0.22	-0.07	0.45
0.819	0.83	0.28	-0.04	0.48
0.821	0.77	0.30	0.00	0.56
0.823	0.81	0.24	0.01	0.54
0.825	0.74	0.24	0.08	0.63
0.826	0.71	0.21	0.07	0.67
0.828	0.67	0.17	0.01	0.72
0.830	0.66	0.18	0.00	0.73
0.832	0.63	0.17	0.11	0.75
0.833	0.56	0.16	0.21	0.78
0.835	0.47	0.20	0.16	0.85
0.837	0.41	0.26	0.06	0.87
0.839	0.42	0.23	0.02	0.88
0.840	0.41	0.28	0.08	0.86
0.842	0.40	0.33	0.10	0.85
0.844	0.39	0.34	0.11	0.85
0.846	0.38	0.33	0.08	0.86

0.847	0.36	0.36	0.05	0.86
0.849	0.33	0.39	0.04	0.86
0.851	0.33	0.44	0.03	0.83
0.853	0.40	0.38	0.03	0.83
0.854	0.44	0.35	0.01	0.82
0.856	0.44	0.30	0.03	0.85
0.858	0.40	0.27	0.10	0.87
0.860	0.39	0.31	0.13	0.86
0.861	0.41	0.36	0.09	0.83
0.863	0.43	0.32	0.09	0.84
0.865	0.43	0.23	0.10	0.87
0.867	0.33	0.21	0.10	0.92
0.868	0.34	0.28	0.14	0.89
0.870	0.40	0.27	0.12	0.87
0.872	0.42	0.15	0.06	0.89
0.874	0.36	0.12	0.03	0.93
0.875	0.30	0.23	0.05	0.92
0.877	0.42	0.26	0.06	0.87
0.879	0.61	0.19	0.05	0.77
0.881	0.64	0.28	-0.05	0.72
0.882	0.65	0.38	-0.09	0.65
0.884	0.54	0.37	-0.13	0.75
0.886	0.46	0.29	-0.24	0.80
0.888	0.57	0.32	-0.32	0.68
0.889	0.60	0.41	-0.32	0.61
0.891	0.53	0.37	-0.33	0.69
0.893	0.45	0.30	-0.39	0.75
0.895	0.60	0.38	-0.04	0.70
0.896	0.55	0.35	-0.16	0.74
0.898	0.48	0.32	-0.22	0.79
0.900	0.45	0.32	-0.17	0.82
0.902	0.51	0.30	-0.12	0.80
0.904	0.58	0.32	-0.15	0.73
0.905	0.57	0.35	-0.16	0.72
0.907	0.53	0.37	-0.14	0.75
0.909	0.56	0.31	-0.20	0.74
0.911	0.45	0.33	-0.03	0.83
0.912	0.43	0.28	0.03	0.86
0.914	0.46	0.26	-0.10	0.84
0.916	0.37	0.28	-0.21	0.86
0.918	0.38	0.29	-0.13	0.87

0.919	0.43	0.33	-0.08	0.84
0.921	0.35	0.39	-0.11	0.84
0.923	0.47	0.41	-0.04	0.78
0.925	0.51	0.30	-0.07	0.80
0.926	0.49	0.37	-0.18	0.77
0.928	0.45	0.44	-0.15	0.77
0.930	0.44	0.29	0.02	0.85
0.932	0.55	0.22	0.07	0.81
0.933	0.63	0.32	-0.08	0.70
0.935	0.60	0.38	-0.21	0.67
0.937	0.52	0.42	-0.37	0.65
0.939	0.51	0.31	-0.31	0.74
0.940	0.55	0.20	-0.21	0.78
0.942	0.58	0.18	-0.10	0.79
0.944	0.58	0.21	-0.12	0.78
0.946	0.58	0.19	-0.15	0.78
0.947	0.54	0.19	-0.23	0.78
0.949	0.61	0.20	-0.28	0.72
0.951	0.41	0.21	-0.25	0.85
0.953	0.33	0.20	-0.02	0.92
0.954	0.36	0.19	-0.05	0.91
0.956	0.57	0.13	-0.22	0.78
0.958	0.64	0.03	-0.29	0.71
0.960	0.48	0.04	-0.39	0.78
0.961	0.29	0.06	-0.54	0.79
0.963	0.24	-0.07	-0.62	0.74
0.965	0.18	-0.06	-0.90	0.39
0.967	-0.00	-0.28	-0.96	0.04
0.968	0.18	-0.23	-0.96	-0.01
0.970	0.22	-0.30	-0.93	-0.04
0.972	-0.09	-0.47	-0.87	-0.12
0.974	-0.20	-0.51	-0.83	-0.08
0.975	-0.19	-0.43	-0.88	-0.04
0.977	-0.25	-0.37	-0.88	-0.15
0.979	-0.19	-0.34	-0.90	-0.21
0.981	-0.29	-0.35	-0.85	-0.25
0.982	-0.35	-0.29	-0.86	-0.24
0.984	-0.29	-0.27	-0.87	-0.30
0.986	-0.18	-0.31	-0.86	-0.37
0.988	-0.05	-0.21	-0.92	-0.32
0.989	-0.05	-0.15	-0.96	-0.25

0.991	-0.23	-0.26	-0.87	-0.34
0	0.86	0.49	-0.08	0.07
0.002	0.87	0.37	-0.26	0.18
0.004	0.85	0.11	-0.51	0.12
0.005	0.90	0.11	-0.43	-0.03
0.007	0.94	0.33	0.06	0.01
0.009	0.94	0.35	-0.02	0.00
0.011	0.85	0.17	-0.46	-0.17
0.012	0.67	0.24	0.12	-0.70
0.014	0.55	0.31	0.21	-0.75
0.016	0.74	0.06	0.22	-0.63
0.018	0.88	0.14	0.21	-0.40
0.019	0.92	0.20	0.25	-0.23
0.021	0.96	0.15	0.25	-0.03
0.023	0.92	0.23	0.32	0.06
0.025	0.84	0.36	0.39	0.06
0.026	0.67	0.43	0.57	0.21
0.028	0.45	0.51	0.66	0.32
0.03	0.40	0.53	0.64	0.38
0.032	0.52	0.55	0.48	0.44
0.033	0.63	0.41	-0.21	0.63
0.035	-0.04	-0.29	-0.96	0.02
0.037	-0.33	-0.39	-0.71	-0.48
0.039	-0.37	-0.51	-0.59	-0.51
0.04	-0.26	-0.58	-0.31	-0.71
0.042	-0.20	-0.67	-0.21	-0.69
0.044	-0.06	-0.71	-0.33	-0.62
0.046	0.07	-0.65	-0.42	-0.63
0.047	0.21	-0.60	-0.16	-0.76
0.049	0.63	-0.50	-0.20	-0.56
0.051	0.86	-0.16	-0.36	-0.33
0.053	0.85	0.01	-0.11	-0.51
0.054	0.82	-0.26	0.12	-0.48
0.056	0.92	-0.01	0.16	-0.37
0.058	0.97	0.14	0.13	-0.17
0.06	0.98	0.09	0.14	-0.13
0.061	0.94	0.04	0.28	-0.22
0.063	0.87	0.08	0.40	-0.27
0.065	0.89	0.03	0.37	-0.26
0.067	0.93	-0.02	0.26	-0.24
0.068	0.94	-0.22	0.24	-0.06

0.07	0.92	-0.13	0.35	-0.11
0.072	0.82	-0.06	0.50	-0.25
0.074	0.80	-0.15	0.57	-0.14
0.075	0.79	-0.22	0.56	0.11
0.077	0.87	-0.14	0.43	0.19
0.079	0.91	0.00	0.38	0.16
0.081	0.82	-0.07	0.49	0.28
0.082	0.70	0.05	0.64	0.32
0.084	0.73	0.05	0.61	0.30
0.086	0.65	-0.05	0.63	0.42
0.088	0.61	-0.19	0.64	0.43
0.089	0.61	-0.37	0.70	0.02
0.091	0.52	-0.47	0.69	-0.16
0.093	0.43	-0.62	0.64	-0.12
0.095	0.50	-0.69	0.41	-0.31
0.096	0.43	-0.59	0.29	-0.62
0.098	0.37	-0.67	0.16	-0.62
0.1	0.46	-0.66	0.27	-0.53
0.102	0.59	-0.60	0.24	-0.49
0.104	0.50	-0.60	0.06	-0.62
0.105	0.65	-0.50	0.26	-0.52
0.107	0.78	-0.39	0.36	-0.34
0.109	0.72	-0.55	0.06	-0.42
0.111	0.85	-0.26	-0.37	-0.27
0.112	0.96	-0.21	-0.12	-0.13
0.114	0.97	-0.19	0.01	-0.16
0.116	0.97	-0.14	-0.01	-0.20
0.118	0.99	-0.05	-0.02	-0.12
0.119	0.99	0.06	0.08	0.08
0.121	1.00	-0.05	-0.01	0.02
0.123	0.93	-0.26	-0.19	-0.17
0.125	0.91	-0.40	0.09	-0.00
0.126	0.93	-0.33	0.06	-0.13
0.128	0.96	-0.20	-0.01	-0.19
0.13	0.99	-0.09	-0.03	-0.12
0.132	0.99	-0.12	0.01	-0.01
0.133	0.99	-0.10	0.01	0.01
0.135	1.00	0.03	0.01	-0.02
0.137	0.99	0.10	0.03	-0.05
0.139	0.99	-0.01	0.02	0.12
0.14	0.97	-0.21	0.09	0.08

0.142	1.00	0.03	-0.02	-0.02
0.144	0.96	0.20	-0.20	-0.02
0.146	0.97	-0.16	-0.10	0.14
0.147	0.97	-0.13	-0.00	0.20
0.149	0.97	0.20	-0.11	0.05
0.151	0.88	0.41	-0.19	-0.15
0.153	0.97	0.14	0.18	0.00
0.154	0.98	0.04	0.17	0.07
0.156	1.00	-0.02	0.06	0.06
0.158	0.99	-0.00	-0.12	0.02
0.16	0.99	0.00	-0.05	-0.10
0.161	0.99	0.03	0.16	-0.06
0.163	0.99	0.05	0.15	0.03
0.165	0.98	0.08	-0.09	0.16
0.167	0.99	-0.13	-0.01	0.04
0.168	0.98	-0.19	-0.02	0.07
0.17	0.98	-0.18	-0.00	-0.08
0.172	0.93	-0.19	-0.08	-0.29
0.174	0.91	-0.25	-0.06	-0.33
0.175	0.93	-0.33	0.02	-0.17
0.177	0.98	-0.15	0.14	0.02
0.179	0.98	0.10	0.19	-0.01
0.181	0.97	0.24	0.07	0.01
0.182	0.98	0.14	-0.07	0.12
0.184	0.98	-0.02	-0.08	0.20
0.186	0.98	-0.07	0.04	0.19
0.188	0.99	-0.04	0.01	0.13
0.189	0.99	0.01	-0.13	0.10
0.191	0.98	-0.01	-0.18	0.08
0.193	0.99	-0.01	-0.13	0.09
0.195	0.96	0.06	-0.08	0.27
0.196	0.98	0.07	-0.11	0.12
0.198	0.98	0.03	-0.19	-0.09
0.2	0.98	0.00	-0.18	-0.11
0.202	0.97	0.16	-0.17	0.08
0.204	0.93	0.29	-0.10	0.19
0.205	0.94	0.23	-0.09	0.21
0.207	0.98	0.09	-0.02	0.15
0.209	0.98	-0.11	-0.02	0.16
0.211	0.98	-0.08	-0.05	0.17
0.212	0.98	-0.04	-0.08	0.18

0.214	0.99	0.02	-0.07	0.13
0.216	1.00	-0.01	-0.04	0.05
0.218	0.99	-0.09	-0.06	-0.02
0.219	0.99	-0.03	-0.09	0.12
0.221	0.96	0.01	-0.12	0.26
0.223	0.99	0.03	-0.11	0.06
0.225	1.00	0.05	0.00	0.06
0.226	0.99	0.05	0.12	0.02
0.228	1.00	-0.05	0.08	0.01
0.23	0.98	-0.17	-0.08	-0.00
0.232	0.95	-0.23	-0.20	-0.01
0.233	0.97	-0.16	-0.18	-0.01
0.235	0.99	-0.11	-0.12	-0.00
0.237	0.95	-0.22	0.22	0.02
0.239	0.97	-0.22	0.01	0.13
0.24	0.96	-0.09	-0.08	0.24
0.242	0.98	0.02	-0.05	0.20
0.244	0.98	0.00	0.02	0.17
0.246	0.95	-0.14	0.19	0.20
0.247	0.92	-0.28	0.25	0.14
0.249	0.95	-0.31	0.05	0.04
0.251	0.98	-0.07	-0.17	0.08
0.253	0.96	-0.09	-0.07	0.26
0.254	0.98	-0.11	0.02	0.19
0.256	0.98	-0.12	0.08	0.11
0.258	0.98	-0.18	0.05	0.08
0.26	0.99	-0.12	-0.02	-0.12
0.261	0.97	-0.05	-0.04	-0.23
0.263	1.00	-0.06	0.02	-0.05
0.265	0.99	-0.02	-0.13	0.02
0.267	0.99	-0.15	-0.08	0.00
0.268	0.99	-0.10	0.03	-0.11
0.27	0.99	-0.02	-0.00	-0.13
0.272	0.99	-0.06	-0.16	-0.00
0.274	0.99	0.03	-0.14	0.09
0.275	0.98	0.10	-0.10	0.10
0.277	0.97	-0.05	-0.23	0.12
0.279	0.95	-0.05	-0.16	0.26
0.281	0.94	-0.00	-0.24	0.25
0.282	0.94	0.07	-0.23	0.25
0.284	0.98	0.03	-0.08	0.20

0.286	0.99	-0.09	0.05	0.06
0.288	0.98	-0.19	0.05	-0.09
0.289	0.99	-0.10	-0.02	-0.05
0.291	0.98	0.12	-0.05	0.12
0.293	0.99	0.06	-0.12	0.10
0.295	1.00	0.03	-0.03	0.09
0.296	1.00	0.01	-0.00	0.09
0.298	0.99	0.04	-0.10	0.10
0.3	0.97	0.10	-0.18	0.16
0.302	0.97	0.13	-0.12	0.17
0.304	0.98	0.08	-0.01	0.20
0.305	0.98	-0.03	0.05	0.20
0.307	1.00	0.01	-0.01	0.09
0.309	0.96	-0.10	-0.23	0.12
0.311	0.99	0.05	-0.15	0.06
0.312	0.96	0.23	0.11	-0.09
0.314	0.99	0.04	0.07	0.06
0.316	0.95	-0.03	-0.11	0.29
0.318	0.94	0.11	-0.11	0.30
0.319	0.98	0.13	0.01	0.14
0.321	0.97	0.17	-0.14	-0.10
0.323	0.98	0.15	-0.10	-0.03
0.325	0.99	0.08	-0.09	0.08
0.326	0.98	0.05	-0.09	0.19
0.328	0.97	0.01	-0.01	0.23
0.33	0.97	0.07	0.01	0.22
0.332	0.99	0.06	-0.01	0.14
0.333	1.00	0.04	-0.07	0.05
0.335	0.98	-0.05	-0.09	0.16
0.337	0.99	0.09	-0.03	0.11
0.339	0.98	0.15	-0.01	0.12
0.34	0.98	0.09	-0.02	0.20
0.342	0.96	0.05	-0.04	0.27
0.344	0.95	0.06	0.02	0.30
0.346	0.96	0.08	0.02	0.28
0.347	0.96	0.07	-0.02	0.26
0.349	0.91	0.14	-0.08	0.39
0.351	0.93	0.14	-0.06	0.32
0.353	0.96	0.19	-0.05	0.20
0.354	0.96	0.22	-0.09	0.14
0.356	0.95	0.23	-0.15	0.12

0.358	0.96	0.20	-0.20	0.10
0.36	0.97	0.16	-0.18	0.02
0.361	0.98	0.15	-0.14	-0.05
0.363	0.96	0.23	-0.05	-0.18
0.365	0.98	0.15	-0.08	-0.13
0.367	0.98	0.02	-0.09	-0.18
0.368	0.98	-0.13	-0.04	-0.16
0.37	1.00	-0.06	-0.01	-0.03
0.372	0.99	0.11	-0.05	0.07
0.374	0.98	0.15	-0.08	0.06
0.375	1.00	0.06	-0.06	0.01
0.377	0.98	-0.05	0.09	0.15
0.379	0.99	-0.04	0.03	0.16
0.381	0.98	0.09	-0.04	0.15
0.382	0.97	0.20	-0.07	0.13
0.384	0.96	0.24	-0.07	0.14
0.386	0.97	0.17	-0.06	0.17
0.388	0.97	0.17	-0.04	0.15
0.389	0.97	0.24	-0.05	0.10
0.391	0.98	0.18	0.00	0.10
0.393	0.98	0.11	0.02	0.14
0.395	0.96	0.22	0.09	0.12
0.396	0.96	0.25	0.06	0.09
0.398	0.99	0.09	-0.05	0.12
0.4	0.99	0.12	-0.02	0.09
0.402	0.96	0.27	-0.01	0.06
0.404	0.96	0.26	-0.12	0.06
0.405	0.99	0.03	0.01	0.09
0.407	0.98	0.16	0.08	0.09
0.409	0.98	0.11	0.09	0.13
0.411	0.98	0.09	0.14	0.13
0.412	0.97	0.23	0.12	0.04
0.414	0.97	0.22	0.07	0.05
0.416	0.99	0.10	0.04	0.13
0.418	0.97	0.09	0.15	0.18
0.419	1.00	0.04	0.02	0.04
0.421	1.00	0.02	0.03	0.02
0.423	0.99	0.07	0.04	0.07
0.425	0.98	0.14	0.02	0.13
0.426	0.97	0.13	0.15	0.12
0.428	0.98	0.03	0.19	0.07

0.43	0.99	0.06	0.06	0.08
0.432	0.97	0.13	-0.14	0.14
0.433	0.95	0.24	0.14	0.16
0.435	0.97	0.10	0.17	0.15
0.437	0.98	0.07	0.13	0.14
0.439	0.98	0.19	0.05	0.10
0.44	0.96	0.25	0.02	0.08
0.442	0.98	0.17	0.07	0.10
0.444	0.99	0.13	0.05	0.11
0.446	0.98	0.16	-0.02	0.10
0.447	0.99	0.13	0.07	0.09
0.449	0.98	0.11	0.09	0.12
0.451	0.98	0.18	0.06	0.11
0.453	0.97	0.23	-0.00	0.07
0.454	0.98	0.18	0.05	0.02
0.456	0.98	0.12	0.13	0.05
0.458	0.97	0.19	0.14	0.05
0.46	0.95	0.32	0.06	0.03
0.461	0.90	0.37	0.25	0.04
0.463	0.89	0.43	0.15	-0.02
0.465	0.88	0.40	0.24	0.02
0.467	0.89	0.36	0.25	0.11
0.468	0.88	0.44	0.14	0.07
0.47	0.87	0.44	0.21	-0.01
0.472	0.89	0.34	0.31	-0.02
0.474	0.94	0.26	0.21	0.08
0.475	0.94	0.32	0.07	0.11
0.477	0.90	0.35	0.20	0.18
0.479	0.90	0.33	0.24	0.17
0.481	0.95	0.26	0.14	0.11
0.482	0.98	0.17	0.07	0.08
0.484	0.98	0.14	0.12	0.13
0.486	0.96	0.14	0.14	0.17
0.488	0.96	0.17	0.12	0.18
0.489	0.97	0.13	0.15	0.16
0.491	0.95	0.16	0.13	0.22
0.493	0.95	0.17	0.13	0.22
0.495	0.95	0.18	0.11	0.23
0.496	0.93	0.25	0.05	0.25
0.498	0.94	0.24	0.05	0.25
0.5	0.94	0.21	0.11	0.25

0.502	0.93	0.20	0.12	0.28
0.504	0.93	0.22	0.06	0.28
0.505	0.95	0.22	0.08	0.22
0.507	0.95	0.23	0.08	0.21
0.509	0.93	0.25	0.05	0.28
0.511	0.90	0.24	0.08	0.35
0.512	0.91	0.22	0.13	0.33
0.514	0.93	0.21	0.14	0.28
0.516	0.93	0.22	0.11	0.27
0.518	0.92	0.25	0.14	0.28
0.519	0.89	0.29	0.16	0.32
0.521	0.87	0.29	0.15	0.38
0.523	0.86	0.25	0.11	0.42
0.525	0.87	0.22	0.11	0.44
0.526	0.86	0.28	0.13	0.40
0.528	0.83	0.37	0.16	0.37
0.53	0.81	0.45	0.16	0.36
0.532	0.70	0.52	0.15	0.46
0.533	0.67	0.62	0.18	0.37
0.535	0.61	0.72	0.18	0.28
0.537	0.55	0.79	0.14	0.22
0.539	0.50	0.85	0.08	0.13
0.54	0.46	0.89	0.05	0.05
0.542	0.43	0.90	0.02	0.01
0.544	0.41	0.91	0.02	0.02
0.546	0.39	0.92	0.06	0.02
0.547	0.42	0.91	0.05	0.01
0.549	0.46	0.89	0.07	0.02
0.551	0.53	0.84	0.07	0.07
0.553	0.58	0.79	0.10	0.17
0.554	0.63	0.72	0.15	0.24
0.556	0.67	0.65	0.19	0.31
0.558	0.70	0.58	0.22	0.34
0.56	0.71	0.53	0.28	0.38
0.561	0.66	0.59	0.28	0.35
0.563	0.68	0.58	0.25	0.37
0.565	0.70	0.50	0.23	0.45
0.567	0.64	0.51	0.27	0.50
0.568	0.62	0.57	0.28	0.46
0.57	0.61	0.61	0.22	0.45
0.572	0.58	0.63	0.15	0.50

0.574	0.52	0.71	0.20	0.43
0.575	0.52	0.71	0.23	0.43
0.577	0.52	0.69	0.25	0.43
0.579	0.51	0.71	0.25	0.41
0.581	0.49	0.75	0.23	0.38
0.582	0.47	0.78	0.22	0.36
0.584	0.49	0.78	0.23	0.31
0.586	0.48	0.77	0.26	0.31
0.588	0.49	0.76	0.33	0.27
0.589	0.58	0.71	0.30	0.28
0.591	0.65	0.67	0.27	0.26
0.593	0.65	0.65	0.31	0.25
0.595	0.64	0.61	0.36	0.29
0.596	0.63	0.58	0.40	0.34
0.598	0.51	0.63	0.46	0.36
0.6	0.43	0.69	0.51	0.28
0.602	0.40	0.65	0.49	0.42
0.604	0.35	0.70	0.51	0.37
0.605	0.30	0.72	0.54	0.32
0.607	0.25	0.73	0.55	0.31
0.609	0.18	0.76	0.55	0.29
0.611	0.14	0.77	0.56	0.25
0.612	0.13	0.75	0.59	0.24
0.614	0.17	0.76	0.59	0.23
0.616	0.11	0.77	0.62	0.12
0.618	0.08	0.80	0.59	0.12
0.619	0.08	0.82	0.56	0.09
0.621	0.11	0.83	0.55	0.03
0.623	0.14	0.82	0.55	0.02
0.625	0.15	0.82	0.55	0.04
0.626	0.16	0.83	0.53	0.02
0.628	0.18	0.84	0.51	-0.04
0.63	0.23	0.85	0.48	-0.04
0.632	0.28	0.83	0.48	-0.01
0.633	0.33	0.82	0.47	0.03
0.635	0.36	0.82	0.44	0.04
0.637	0.42	0.80	0.41	0.08
0.639	0.52	0.75	0.40	0.11
0.64	0.62	0.68	0.37	0.15
0.642	0.68	0.64	0.33	0.16
0.644	0.70	0.56	0.40	0.21

0.646	0.75	0.50	0.34	0.27
0.647	0.79	0.43	0.28	0.32
0.649	0.82	0.38	0.29	0.32
0.651	0.82	0.34	0.34	0.32
0.653	0.82	0.35	0.35	0.28
0.654	0.84	0.37	0.29	0.28
0.656	0.84	0.39	0.22	0.31
0.658	0.86	0.35	0.24	0.30
0.66	0.85	0.34	0.25	0.31
0.661	0.84	0.35	0.25	0.33
0.663	0.82	0.39	0.25	0.33
0.665	0.83	0.39	0.29	0.28
0.667	0.83	0.40	0.33	0.21
0.668	0.82	0.43	0.31	0.22
0.67	0.80	0.46	0.26	0.28
0.672	0.84	0.32	0.36	0.27
0.674	0.82	0.30	0.40	0.29
0.675	0.80	0.35	0.39	0.30
0.677	0.79	0.40	0.32	0.34
0.679	0.82	0.37	0.30	0.31
0.681	0.88	0.29	0.30	0.26
0.682	0.86	0.29	0.32	0.26
0.684	0.78	0.39	0.36	0.32
0.686	0.82	0.29	0.45	0.20
0.688	0.81	0.29	0.43	0.26
0.689	0.78	0.35	0.42	0.29
0.691	0.75	0.47	0.39	0.25
0.693	0.70	0.52	0.42	0.27
0.695	0.66	0.53	0.38	0.38
0.696	0.63	0.51	0.38	0.44
0.698	0.66	0.49	0.37	0.43
0.7	0.65	0.51	0.38	0.42
0.702	0.71	0.47	0.31	0.42
0.704	0.69	0.51	0.28	0.42
0.705	0.67	0.52	0.27	0.45
0.707	0.70	0.46	0.23	0.50
0.709	0.70	0.49	0.23	0.46
0.711	0.69	0.55	0.23	0.41
0.712	0.71	0.53	0.18	0.43
0.714	0.66	0.49	0.31	0.48
0.716	0.66	0.51	0.33	0.44

0.718	0.69	0.51	0.30	0.42
0.719	0.72	0.53	0.24	0.37
0.721	0.72	0.55	0.24	0.35
0.723	0.72	0.54	0.27	0.33
0.725	0.75	0.50	0.29	0.32
0.726	0.78	0.47	0.27	0.30
0.728	0.80	0.45	0.24	0.32
0.73	0.80	0.47	0.27	0.26
0.732	0.83	0.43	0.28	0.23
0.733	0.85	0.37	0.26	0.27
0.735	0.83	0.37	0.25	0.32
0.737	0.82	0.41	0.24	0.31
0.739	0.85	0.40	0.24	0.26
0.74	0.89	0.33	0.23	0.22
0.742	0.87	0.38	0.14	0.30
0.744	0.83	0.37	0.22	0.34
0.746	0.84	0.41	0.13	0.32
0.747	0.86	0.40	0.11	0.28
0.749	0.85	0.33	0.26	0.31
0.751	0.82	0.31	0.25	0.41
0.753	0.83	0.36	0.16	0.39
0.754	0.85	0.37	0.24	0.27
0.756	0.83	0.35	0.26	0.34
0.758	0.83	0.28	0.27	0.39
0.76	0.87	0.24	0.17	0.40
0.761	0.89	0.24	0.15	0.35
0.763	0.85	0.23	0.24	0.41
0.765	0.82	0.23	0.18	0.49
0.767	0.87	0.24	0.11	0.41
0.768	0.91	0.19	0.23	0.28
0.77	0.88	0.33	0.11	0.32
0.772	0.88	0.28	0.17	0.33
0.774	0.89	0.23	0.21	0.34
0.775	0.90	0.23	0.17	0.32
0.777	0.92	0.25	0.12	0.29
0.779	0.91	0.31	0.12	0.26
0.781	0.89	0.31	0.17	0.28
0.782	0.87	0.30	0.22	0.33
0.784	0.93	0.25	0.13	0.25
0.786	0.91	0.22	0.18	0.29
0.788	0.89	0.22	0.25	0.30

0.789	0.89	0.24	0.25	0.31
0.791	0.88	0.31	0.24	0.28
0.793	0.88	0.34	0.23	0.26
0.795	0.88	0.33	0.25	0.26
0.796	0.88	0.29	0.27	0.25
0.798	0.85	0.40	0.25	0.24
0.8	0.80	0.45	0.31	0.26
0.802	0.76	0.47	0.34	0.28
0.804	0.78	0.45	0.33	0.30
0.805	0.78	0.45	0.31	0.30
0.807	0.75	0.50	0.32	0.29
0.809	0.73	0.53	0.34	0.27
0.811	0.72	0.54	0.36	0.25
0.812	0.75	0.51	0.36	0.21
0.814	0.75	0.50	0.39	0.20
0.816	0.72	0.51	0.39	0.25
0.818	0.69	0.55	0.38	0.28
0.819	0.69	0.57	0.38	0.24
0.821	0.71	0.58	0.35	0.18
0.823	0.71	0.58	0.35	0.19
0.825	0.70	0.57	0.34	0.27
0.826	0.69	0.57	0.33	0.28
0.828	0.68	0.58	0.34	0.28
0.83	0.67	0.59	0.32	0.31
0.832	0.68	0.59	0.31	0.31
0.833	0.68	0.58	0.30	0.33
0.835	0.68	0.57	0.30	0.35
0.837	0.66	0.58	0.30	0.38
0.839	0.65	0.58	0.31	0.40
0.84	0.64	0.57	0.29	0.42
0.842	0.65	0.59	0.29	0.37
0.844	0.65	0.61	0.28	0.35
0.846	0.63	0.63	0.25	0.37
0.847	0.62	0.63	0.23	0.40
0.849	0.63	0.62	0.23	0.41
0.851	0.64	0.61	0.22	0.42
0.853	0.64	0.60	0.20	0.44
0.854	0.63	0.62	0.13	0.44
0.856	0.66	0.59	0.12	0.45
0.858	0.60	0.65	0.15	0.45
0.86	0.53	0.67	0.20	0.48

0.861	0.54	0.64	0.18	0.53
0.863	0.54	0.67	0.21	0.46
0.865	0.55	0.68	0.26	0.41
0.867	0.57	0.62	0.27	0.46
0.868	0.61	0.60	0.17	0.49
0.87	0.62	0.61	0.15	0.46
0.872	0.59	0.64	0.13	0.47
0.874	0.56	0.64	0.13	0.51
0.875	0.60	0.60	0.10	0.52
0.877	0.65	0.58	0.14	0.48
0.879	0.62	0.60	0.18	0.47
0.881	0.53	0.63	0.23	0.52
0.882	0.64	0.68	0.21	0.29
0.884	0.64	0.66	0.17	0.36
0.886	0.63	0.61	0.18	0.44
0.888	0.64	0.55	0.23	0.48
0.889	0.69	0.47	0.28	0.47
0.891	0.71	0.45	0.31	0.44
0.893	0.69	0.49	0.31	0.43
0.895	0.65	0.56	0.30	0.42
0.896	0.65	0.52	0.33	0.45
0.898	0.69	0.41	0.39	0.45
0.9	0.73	0.36	0.40	0.42
0.902	0.73	0.44	0.38	0.37
0.904	0.71	0.51	0.35	0.34
0.905	0.72	0.46	0.37	0.36
0.907	0.72	0.41	0.39	0.40
0.909	0.71	0.40	0.39	0.43
0.911	0.72	0.36	0.39	0.45
0.912	0.73	0.41	0.37	0.40
0.914	0.75	0.33	0.31	0.47
0.916	0.76	0.27	0.31	0.51
0.918	0.76	0.32	0.38	0.42
0.919	0.74	0.30	0.42	0.43
0.921	0.74	0.23	0.39	0.50
0.923	0.76	0.28	0.37	0.46
0.925	0.75	0.31	0.41	0.42
0.926	0.76	0.28	0.39	0.42
0.928	0.77	0.23	0.36	0.46
0.93	0.76	0.17	0.33	0.53
0.932	0.76	0.15	0.33	0.54

0.933	0.77	0.18	0.36	0.50
0.935	0.75	0.18	0.39	0.51
0.937	0.72	0.16	0.39	0.55
0.939	0.73	0.13	0.33	0.58
0.94	0.73	0.12	0.33	0.59
0.942	0.73	0.10	0.33	0.59
0.944	0.73	0.08	0.34	0.58
0.946	0.75	0.06	0.35	0.57
0.947	0.76	0.04	0.33	0.56
0.949	0.77	0.03	0.31	0.55
0.951	0.80	0.04	0.29	0.53
0.953	0.84	0.03	0.28	0.47
0.954	0.88	-0.00	0.23	0.43
0.956	0.89	-0.03	0.21	0.41
0.958	0.87	-0.03	0.23	0.43
0.96	0.86	-0.01	0.25	0.44
0.961	0.87	0.01	0.25	0.42
0.963	0.87	-0.02	0.21	0.44
0.965	0.86	-0.06	0.18	0.47
0.967	0.84	-0.04	0.22	0.50
0.968	0.85	-0.00	0.25	0.46
0.97	0.81	0.07	0.31	0.50
0.972	0.70	0.11	0.33	0.62
0.974	0.69	0.15	0.33	0.62
0.975	0.65	0.32	0.42	0.55
0.977	0.43	0.53	0.51	0.53
0.979	0.35	0.56	0.49	0.57
0.981	0.20	0.69	0.36	0.60
0.982	0.02	0.75	0.43	0.50
0.984	-0.13	0.78	0.44	0.42
0.986	-0.21	0.74	0.42	0.47
0.988	-0.25	0.60	0.34	0.68
0.989	-0.28	0.35	0.23	0.86
0.991	-0.32	0.16	0.18	0.91
0.993	-0.37	0.11	0.18	0.90
0.995	-0.26	-0.04	0.02	0.96
0.996	-0.15	-0.10	0.14	0.97
0.998	-0.10	-0.17	0.19	0.96
1	-0.15	-0.25	0.14	0.95

Variable/ Rf	Factor Loadings (Varimax normalized, data from chromatograms on RP-C18) Extraction: Principal components (Marked loadings are >.700000)			
	Factor1	Factor2	Factor 3	Factor4
0.000	-0.35	-0.76	-0.5373	-0.06068
0.002	-0.27	-0.77	0.0868	-0.56541
0.004	-0.15	-0.85	0.3941	-0.30471
0.005	0.12	-0.92	0.2197	0.29175
0.007	-0.02	-0.88	-0.0676	0.46160
0.009	-0.26	-0.87	-0.1382	0.38290
0.011	-0.85	-0.42	-0.0341	-0.31336
0.012	-0.78	-0.38	-0.3036	-0.40386
0.014	-0.73	-0.35	-0.4238	-0.39730
0.016	-0.86	-0.35	-0.3354	0.14631
0.018	-0.77	-0.57	-0.2556	0.11885
0.019	-0.84	-0.40	-0.2874	-0.22912
0.021	-0.99	0.06	-0.0505	-0.12796
0.023	-0.91	0.38	0.1269	0.10605
0.025	-0.88	0.42	0.2175	-0.04541
0.026	-0.75	0.47	0.4523	0.09093
0.028	-0.62	0.55	0.5630	0.03036
0.030	-0.57	0.60	0.5347	-0.15300
0.032	-0.63	0.56	0.4085	-0.36475
0.033	-0.77	0.29	0.1639	-0.54065
0.035	-0.79	-0.35	-0.1731	-0.46648
0.037	-0.55	-0.75	-0.2994	-0.21280
0.039	-0.44	-0.84	-0.2345	-0.20346
0.040	-0.50	-0.82	-0.1819	-0.21505
0.042	-0.41	-0.86	-0.1799	-0.23926
0.044	-0.36	-0.88	-0.1560	-0.26395
0.046	-0.51	-0.83	-0.0321	-0.20466
0.047	-0.62	-0.78	0.0324	-0.09970
0.049	-0.67	-0.74	0.0051	-0.05404
0.051	-0.73	-0.67	0.0706	-0.11320
0.053	-0.79	-0.58	-0.1346	0.15163
0.054	-0.73	-0.65	-0.1548	0.13369
0.056	-0.71	-0.65	-0.2274	0.14458
0.058	-0.77	-0.55	-0.3088	0.02814
0.060	-0.89	-0.35	-0.2740	0.03565
0.061	-0.89	-0.36	-0.2758	0.06739

0.063	-0.88	-0.35	-0.2880	0.12293
0.065	-0.82	-0.36	-0.3189	0.30668
0.067	-0.78	-0.39	-0.1477	0.46104
0.068	-0.78	-0.50	0.0280	0.36454
0.070	-0.78	-0.52	0.0053	0.34625
0.072	-0.75	-0.45	-0.2419	0.42112
0.074	-0.78	-0.47	-0.2905	0.27515
0.075	-0.79	-0.62	0.0215	0.04505
0.077	-0.81	-0.51	0.2362	0.14542
0.079	-0.77	-0.27	0.3496	0.45208
0.081	-0.73	-0.48	0.4776	-0.10521
0.082	-0.71	-0.60	0.0017	-0.37031
0.084	-0.78	-0.59	-0.0579	-0.21350
0.086	-0.82	-0.53	0.1470	0.17554
0.088	-0.86	-0.45	-0.0008	0.25068
0.089	-0.92	-0.14	-0.3708	0.05350
0.091	-0.95	-0.12	-0.2971	-0.00216
0.093	-0.88	-0.26	-0.0513	0.40561
0.095	-0.96	-0.05	0.0925	0.24791
0.096	-0.96	0.23	0.1395	0.09645
0.098	-0.96	0.27	-0.0411	-0.08045
0.100	-0.96	0.23	-0.1460	-0.00722
0.102	-0.95	0.24	-0.1470	0.13870
0.104	-0.96	-0.00	-0.1508	0.24984
0.105	-0.96	-0.10	-0.0357	0.24290
0.107	-0.94	0.26	0.0895	0.21878
0.109	-0.87	0.34	-0.2849	0.19856
0.111	-0.96	0.15	-0.1568	0.16010
0.112	-0.99	-0.09	-0.0396	0.05364
0.114	-1.00	-0.04	0.0337	-0.02989
0.116	-0.97	0.24	0.0523	-0.07704
0.118	-0.89	0.41	0.0351	-0.16646
0.119	-0.87	0.46	0.0576	-0.19594
0.121	-0.87	0.42	0.0693	-0.25816
0.123	-0.84	0.47	-0.1280	-0.23172
0.125	-0.81	0.49	-0.2863	-0.14609
0.126	-0.70	0.64	-0.1974	-0.25792
0.128	-0.67	0.56	-0.0427	-0.48447
0.130	-0.79	0.30	-0.1088	-0.53226

0.132	-0.79	0.29	-0.1981	-0.50089
0.133	-0.76	0.39	-0.2370	-0.45910
0.135	-0.82	0.26	-0.3727	-0.35288
0.137	-0.84	0.10	-0.1793	-0.49557
0.139	-0.92	0.07	-0.1032	-0.37893
0.140	-0.94	-0.02	-0.0100	-0.33210
0.142	-0.87	-0.16	0.0229	-0.47006
0.144	-0.69	-0.31	0.0082	-0.65404
0.146	-0.80	-0.27	-0.1995	-0.49810
0.147	-0.94	-0.16	-0.1854	-0.24430
0.149	-0.98	-0.15	-0.1061	0.04695
0.151	-0.51	-0.82	-0.2244	-0.16627
0.153	-0.76	-0.65	-0.0168	0.05497
0.154	-0.63	-0.73	0.2452	0.08139
0.156	-0.47	-0.88	-0.0243	-0.02595
0.158	-0.49	-0.85	-0.1882	0.03370
0.160	-0.46	-0.85	0.2589	0.01889
0.161	-0.22	-0.80	0.5630	-0.01521
0.163	-0.44	-0.90	0.0373	0.00677
0.165	-0.71	-0.65	0.2507	0.08438
0.167	-0.60	-0.69	0.3600	0.17815
0.168	-0.41	-0.78	0.2781	0.36644
0.170	-0.31	-0.81	0.0960	0.49116
0.172	-0.53	-0.70	0.1412	0.46756
0.174	-0.67	-0.57	0.3890	0.27093
0.175	-0.52	-0.64	0.5425	0.15588
0.177	-0.27	-0.83	0.4826	0.04253
0.179	-0.42	-0.85	0.3203	-0.04500
0.181	-0.38	-0.77	0.4871	-0.15763
0.182	-0.51	-0.70	0.4937	-0.06295
0.184	-0.71	-0.62	0.2967	0.12589
0.186	-0.75	-0.59	0.2426	0.15071
0.188	-0.63	-0.70	0.3269	-0.00605
0.189	-0.60	-0.75	0.2532	-0.05991
0.191	-0.64	-0.76	0.0269	0.05315
0.193	-0.56	-0.67	0.4803	0.12919
0.195	-0.66	-0.71	0.2498	-0.09086
0.196	-0.73	-0.65	0.0571	-0.20803
0.198	-0.69	-0.72	-0.0301	0.05922

0.200	-0.50	-0.80	-0.0600	0.33972
0.202	-0.54	-0.84	0.0433	-0.00311
0.204	-0.61	-0.74	0.1110	-0.25074
0.205	-0.67	-0.73	0.1168	-0.06724
0.207	-0.66	-0.72	0.2416	0.02145
0.209	-0.57	-0.78	0.2109	0.11973
0.211	-0.50	-0.84	0.1744	0.11761
0.212	-0.57	-0.80	0.1763	0.00142
0.214	-0.57	-0.81	0.0897	-0.10298
0.216	-0.38	-0.89	-0.0689	-0.22462
0.218	-0.33	-0.92	-0.0528	-0.20273
0.219	-0.44	-0.89	0.0470	-0.10145
0.221	-0.55	-0.81	-0.1423	0.12527
0.223	-0.67	-0.74	0.0183	0.05330
0.225	-0.60	-0.79	0.1094	-0.03064
0.226	-0.48	-0.88	0.0053	-0.05696
0.228	-0.61	-0.79	-0.0400	0.00087
0.230	-0.70	-0.71	-0.0623	0.03329
0.232	-0.64	-0.77	0.0165	-0.05131
0.233	-0.59	-0.79	0.1399	-0.11078
0.235	-0.67	-0.74	0.0728	-0.01693
0.237	-0.48	-0.87	-0.0113	-0.15754
0.239	-0.48	-0.85	0.0452	-0.19930
0.240	-0.60	-0.79	0.1540	-0.05318
0.242	-0.58	-0.81	0.0324	-0.00551
0.244	-0.58	-0.80	-0.0864	-0.07490
0.246	-0.68	-0.73	-0.0145	-0.06244
0.247	-0.71	-0.69	0.1289	0.03337
0.249	-0.50	-0.86	-0.0312	0.12998
0.251	-0.26	-0.95	-0.0688	0.15240
0.253	-0.29	-0.95	0.0872	0.01475
0.254	-0.35	-0.90	0.2379	-0.14185
0.256	-0.22	-0.95	0.1204	-0.17434
0.258	-0.15	-0.99	0.0085	-0.02057
0.260	-0.26	-0.96	0.1038	0.00603
0.261	-0.29	-0.93	0.1726	-0.13028
0.263	-0.48	-0.85	0.1837	-0.10894
0.265	-0.32	-0.92	0.1786	-0.12004
0.267	-0.18	-0.97	0.1141	-0.14519

0.268	-0.18	-0.97	0.0922	-0.11401
0.270	-0.23	-0.96	0.1110	-0.12574
0.272	-0.24	-0.95	0.1412	-0.13534
0.274	-0.26	-0.95	0.1060	-0.15491
0.275	-0.31	-0.94	0.0333	-0.13013
0.277	-0.40	-0.87	-0.0581	-0.27060
0.279	-0.18	-0.92	0.1624	-0.29403
0.281	-0.25	-0.91	0.2714	-0.19924
0.282	-0.44	-0.87	0.1839	-0.11057
0.284	-0.38	-0.91	0.0911	-0.14475
0.286	-0.30	-0.91	0.2652	-0.07364
0.288	-0.37	-0.87	0.3340	-0.02581
0.289	-0.39	-0.86	0.2889	-0.18321
0.291	-0.35	-0.88	0.3161	-0.08462
0.293	-0.32	-0.82	0.4110	-0.24440
0.295	-0.09	-0.83	0.4518	-0.31960
0.296	0.01	-0.79	0.4751	-0.39582
0.298	0.08	-0.84	0.4882	-0.21638
0.300	0.05	-0.82	0.5705	0.03512
0.302	0.23	-0.77	0.5887	0.00774
0.304	0.34	-0.69	0.5945	-0.21801
0.305	0.15	-0.89	0.3726	-0.20389
0.307	0.30	-0.87	0.3892	0.00775
0.309	0.37	-0.82	0.4254	0.12682
0.311	0.31	-0.86	0.3988	0.09644
0.312	0.07	-0.94	0.2911	0.16070
0.314	0.07	-0.92	0.3146	0.20189
0.316	0.10	-0.91	0.3587	0.20608
0.318	0.07	-0.92	0.3177	0.21132
0.319	-0.18	-0.95	0.2053	0.12604
0.321	-0.12	-0.98	0.1366	-0.05265
0.323	-0.12	-0.96	0.1839	-0.14591
0.325	-0.10	-0.97	0.2107	0.00384
0.326	-0.19	-0.96	0.1655	0.13339
0.328	-0.23	-0.95	0.1217	0.15945
0.330	-0.27	-0.93	0.2043	0.11890
0.332	-0.24	-0.93	0.2536	0.06283
0.333	-0.17	-0.94	0.2807	0.01986
0.335	-0.11	-0.94	0.2843	0.17647

0.337	-0.00	-0.92	0.2593	0.28748
0.339	0.05	-0.93	0.2618	0.24821
0.340	-0.03	-0.90	0.3608	0.25504
0.342	-0.07	-0.87	0.3376	0.35060
0.344	-0.01	-0.96	0.1627	0.21589
0.346	0.13	-0.98	-0.0924	-0.07288
0.347	-0.07	-0.98	-0.1596	0.03213
0.349	-0.05	-0.99	-0.0832	-0.04685
0.351	-0.00	-0.99	0.1649	-0.01648
0.353	0.04	-0.98	0.1953	-0.01944
0.354	0.15	-0.98	-0.0431	-0.09527
0.356	0.16	-0.98	-0.0384	-0.12141
0.358	0.17	-0.97	0.1276	-0.11130
0.360	0.09	-0.97	0.0972	-0.21845
0.361	0.49	-0.84	0.1876	0.12656
0.363	0.38	-0.92	-0.0364	0.05663
0.365	0.36	-0.91	-0.1976	-0.07270
0.367	0.47	-0.86	-0.1605	-0.14160
0.368	0.53	-0.82	-0.1638	-0.13019
0.370	0.47	-0.83	-0.2770	-0.12960
0.372	0.56	-0.80	-0.2081	-0.01837
0.374	0.71	-0.69	-0.0680	0.09112
0.375	0.66	-0.61	-0.2900	0.31597
0.377	0.65	-0.69	-0.1590	0.26415
0.379	0.60	-0.80	-0.0023	0.04639
0.381	0.62	-0.78	-0.0458	-0.09331
0.382	0.74	-0.63	-0.1863	-0.14739
0.384	0.70	-0.70	-0.1496	-0.03023
0.386	0.50	-0.86	0.0038	0.10717
0.388	0.50	-0.83	0.2068	0.14957
0.389	0.22	-0.81	0.5201	-0.15308
0.391	0.25	-0.61	0.6594	-0.36234
0.393	0.35	-0.43	0.6492	-0.52524
0.395	0.28	-0.41	0.6359	-0.59099
0.396	0.16	-0.46	0.7174	-0.49583
0.398	0.31	-0.45	0.7607	-0.35580
0.400	0.32	-0.34	0.7884	-0.40213
0.402	0.16	-0.32	0.8426	-0.40155
0.404	0.35	-0.13	0.9122	-0.16853

0.405	0.38	-0.13	0.9120	0.06830
0.407	0.32	-0.14	0.8853	0.30208
0.409	0.27	-0.12	0.8930	0.33962
0.411	0.25	-0.04	0.8975	0.36125
0.412	0.29	0.07	0.8638	0.40839
0.414	0.31	0.05	0.8356	0.45210
0.416	0.27	0.01	0.8289	0.48865
0.418	0.32	0.05	0.7751	0.54281
0.419	0.26	0.08	0.7908	0.55114
0.421	0.21	0.06	0.7860	0.57723
0.423	0.21	0.04	0.7981	0.56196
0.425	0.25	0.02	0.8021	0.54139
0.426	0.31	0.00	0.7949	0.52059
0.428	0.37	-0.08	0.7764	0.50515
0.430	0.40	-0.21	0.7480	0.48942
0.432	0.51	-0.39	0.6690	0.36541
0.433	0.60	-0.46	0.6201	0.21293
0.435	0.67	-0.49	0.5320	0.16613
0.437	0.69	-0.53	0.4457	0.21975
0.439	0.69	-0.57	0.3842	0.21828
0.440	0.68	-0.57	0.4084	0.21361
0.442	0.71	-0.55	0.3790	0.24054
0.444	0.73	-0.54	0.3175	0.26918
0.446	0.73	-0.55	0.3578	0.19454
0.447	0.73	-0.52	0.3846	0.22320
0.449	0.71	-0.49	0.4297	0.26113
0.451	0.71	-0.48	0.4294	0.29099
0.453	0.73	-0.46	0.4412	0.25149
0.454	0.73	-0.43	0.4973	0.19212
0.456	0.70	-0.37	0.5878	0.15004
0.458	0.66	-0.31	0.6606	0.17441
0.460	0.72	-0.34	0.6055	0.06557
0.461	0.71	-0.32	0.6202	0.03245
0.463	0.67	-0.27	0.6960	-0.01446
0.465	0.67	-0.24	0.7076	0.02570
0.467	0.70	-0.25	0.6691	0.05716
0.468	0.70	-0.09	0.7048	0.03999
0.470	0.65	-0.07	0.7516	-0.03333
0.472	0.69	-0.16	0.7100	0.00466

0.474	0.68	-0.21	0.6935	-0.10230
0.475	0.76	-0.12	0.6240	-0.13318
0.477	0.80	-0.04	0.5720	-0.18283
0.479	0.74	0.01	0.6004	-0.30661
0.481	0.76	-0.04	0.5228	-0.37562
0.482	0.82	-0.09	0.4525	-0.33031
0.484	0.86	-0.16	0.3842	-0.28876
0.486	0.86	-0.18	0.3772	-0.28359
0.488	0.86	0.01	0.4029	-0.31686
0.489	0.91	0.04	0.2423	-0.33304
0.491	0.93	-0.02	0.1593	-0.34200
0.493	0.93	-0.05	0.1314	-0.34507
0.495	0.92	0.05	0.1888	-0.34731
0.496	0.93	0.07	0.1917	-0.30338
0.498	0.95	0.08	0.1017	-0.27550
0.500	0.96	0.13	0.0276	-0.24434
0.502	0.97	0.15	0.0032	-0.21696
0.504	0.97	0.09	0.0187	-0.22419
0.505	0.97	0.09	0.0207	-0.23248
0.507	0.97	0.14	-0.0080	-0.20387
0.509	0.98	0.15	-0.0587	-0.13962
0.511	0.99	0.10	-0.1060	-0.06886
0.512	0.99	0.07	-0.1301	-0.02888
0.514	0.99	0.06	-0.1334	-0.00805
0.516	0.98	0.07	-0.1748	-0.05084
0.518	0.98	0.06	-0.1846	-0.03430
0.519	0.98	0.05	-0.1897	-0.03042
0.521	0.98	0.03	-0.1808	-0.02875
0.523	0.99	0.01	-0.1608	-0.02590
0.525	0.99	-0.02	-0.1426	-0.01263
0.526	0.99	-0.07	-0.1145	0.01044
0.528	0.99	-0.13	-0.0719	0.02979
0.530	0.98	-0.14	-0.1574	-0.05094
0.532	0.99	-0.10	-0.0865	-0.01685
0.533	0.99	-0.15	-0.0315	0.01342
0.535	0.98	-0.18	0.0238	0.01555
0.537	0.98	-0.16	0.1002	0.01703
0.539	0.96	-0.25	0.0822	0.07650
0.540	0.95	-0.28	0.0391	0.13036

0.542	0.98	-0.15	0.0937	0.05586
0.544	0.89	-0.29	0.3611	0.02011
0.546	0.91	-0.30	0.2918	0.03185
0.547	0.94	-0.24	0.2364	0.04707
0.549	0.96	-0.16	0.2052	0.10233
0.551	0.93	-0.21	0.2704	0.13870
0.553	0.90	-0.30	0.2934	0.14169
0.554	0.89	-0.29	0.3406	0.08724
0.556	0.89	-0.24	0.3764	0.06382
0.558	0.93	-0.16	0.3122	0.14453
0.560	0.86	-0.29	0.4098	0.10114
0.561	0.83	-0.29	0.4601	0.12843
0.563	0.87	-0.18	0.3982	0.21978
0.565	0.90	-0.10	0.3446	0.24238
0.567	0.91	-0.17	0.3348	0.15035
0.568	0.91	-0.17	0.3729	0.10647
0.570	0.91	-0.09	0.3980	0.10139
0.572	0.90	-0.14	0.3442	0.22004
0.574	0.90	-0.17	0.3360	0.20642
0.575	0.91	-0.18	0.3020	0.20603
0.577	0.93	-0.18	0.2448	0.22241
0.579	0.92	-0.19	0.1987	0.29102
0.581	0.90	-0.23	0.2408	0.27276
0.582	0.93	-0.24	0.2259	0.14770
0.584	0.96	-0.19	0.2107	0.02619
0.586	0.92	-0.36	0.1670	0.03068
0.588	0.89	-0.39	0.2417	0.02010
0.589	0.86	-0.41	0.2945	0.05622
0.591	0.86	-0.38	0.3116	0.15743
0.593	0.82	-0.38	0.3237	0.26903
0.595	0.79	-0.40	0.3781	0.27650
0.596	0.82	-0.44	0.3125	0.19369
0.598	0.87	-0.42	0.2003	0.15535
0.600	0.86	-0.43	0.2790	0.06841
0.602	0.85	-0.45	0.2641	0.08228
0.604	0.84	-0.49	0.2355	0.08008
0.605	0.82	-0.52	0.2068	0.08373
0.607	0.80	-0.56	0.1733	0.13465
0.609	0.79	-0.56	0.1607	0.19009

0.611	0.79	-0.54	0.2403	0.17828
0.612	0.80	-0.50	0.3176	0.11904
0.614	0.84	-0.43	0.2734	0.18577
0.616	0.84	-0.42	0.3308	0.14456
0.618	0.84	-0.43	0.2729	0.20682
0.619	0.87	-0.40	0.2169	0.19824
0.621	0.91	-0.35	0.1824	0.11775
0.623	0.91	-0.34	0.1383	0.21410
0.625	0.89	-0.31	0.1660	0.28909
0.626	0.90	-0.27	0.3027	0.16399
0.628	0.94	-0.33	0.0799	0.01915
0.630	0.92	-0.36	0.1454	0.01070
0.632	0.92	-0.35	0.1482	0.06205
0.633	0.95	-0.28	0.0380	0.09997
0.635	0.97	-0.22	0.0623	0.03122
0.637	0.96	-0.22	0.1612	-0.03912
0.639	0.95	-0.24	0.1780	0.02068
0.640	0.95	-0.23	0.1133	0.18992
0.642	0.93	-0.18	0.1647	0.28892
0.644	0.95	-0.26	0.1522	0.06064
0.646	0.96	-0.22	0.1660	-0.05654
0.647	0.98	-0.08	0.1981	0.01902
0.649	0.98	-0.07	0.1932	0.07907
0.651	0.97	-0.21	0.0993	0.01057
0.653	0.96	-0.22	0.1438	-0.06180
0.654	0.96	-0.14	0.2085	-0.08065
0.656	0.95	-0.20	0.1668	0.14511
0.658	0.93	-0.33	0.1502	0.04676
0.660	0.93	-0.29	0.2133	-0.04977
0.661	0.91	-0.28	0.2646	-0.13049
0.663	0.89	-0.39	0.1689	-0.15699
0.665	0.93	-0.32	0.0849	-0.17768
0.667	0.95	-0.23	0.1823	-0.08522
0.668	0.88	-0.26	0.3933	0.03276
0.670	0.86	-0.34	0.2876	-0.23653
0.672	0.85	-0.38	0.1504	-0.34381
0.674	0.87	-0.32	0.2375	-0.28025
0.675	0.86	-0.29	0.3371	-0.25704
0.677	0.83	-0.35	0.2920	-0.33312

0.679	0.83	-0.36	0.2532	-0.33958
0.681	0.86	-0.31	0.3304	-0.23761
0.682	0.91	-0.26	0.2552	-0.18918
0.684	0.86	-0.40	0.2463	-0.19613
0.686	0.89	-0.38	0.1194	-0.21912
0.688	0.94	-0.26	0.1636	-0.18350
0.689	0.96	-0.05	0.2079	-0.18918
0.691	0.97	0.00	0.0226	-0.26083
0.693	0.96	-0.06	-0.0749	-0.24921
0.695	0.96	-0.08	-0.0303	-0.26044
0.696	0.92	-0.07	-0.0443	-0.37236
0.698	0.89	0.02	0.0910	-0.45274
0.700	0.92	0.02	-0.0144	-0.39011
0.702	0.93	0.03	-0.0849	-0.36088
0.704	0.92	0.05	-0.0758	-0.36911
0.705	0.93	0.12	-0.0095	-0.35865
0.707	0.93	0.17	-0.0105	-0.31584
0.709	0.93	0.16	-0.0910	-0.31003
0.711	0.92	0.13	-0.1775	-0.32841
0.712	0.94	0.14	-0.1386	-0.27852
0.714	0.94	0.19	-0.1470	-0.25783
0.716	0.94	0.21	-0.1481	-0.22196
0.718	0.95	0.20	-0.1356	-0.18739
0.719	0.96	0.20	-0.1056	-0.18009
0.721	0.96	0.19	-0.1024	-0.20503
0.723	0.96	0.19	-0.1004	-0.19023
0.725	0.96	0.22	-0.1061	-0.16108
0.726	0.93	0.27	-0.1221	-0.21992
0.728	0.95	0.23	-0.1172	-0.17094
0.730	0.96	0.21	-0.1254	-0.12496
0.732	0.96	0.20	-0.1081	-0.14205
0.733	0.97	0.18	-0.0436	-0.13979
0.735	0.98	0.14	-0.0085	-0.14149
0.737	0.98	0.13	-0.0498	-0.16767
0.739	0.95	0.17	-0.1057	-0.24341
0.740	0.98	0.15	0.0135	-0.15221
0.742	0.97	0.18	0.0230	-0.17935
0.744	0.96	0.16	0.0207	-0.23087
0.746	0.95	0.15	-0.0010	-0.27458

0.747	0.95	0.16	0.0565	-0.24926
0.749	0.96	0.13	0.1248	-0.20772
0.751	0.97	0.11	0.1123	-0.19294
0.753	0.97	0.09	0.0651	-0.22520
0.754	0.89	0.04	0.3476	-0.30262
0.756	0.89	-0.03	0.2494	-0.38650
0.758	0.83	-0.12	0.2185	-0.49144
0.760	0.80	-0.13	0.2896	-0.51059
0.761	0.82	-0.02	0.3410	-0.45115
0.763	0.82	0.13	0.3169	-0.46035
0.765	0.80	0.14	0.2918	-0.49901
0.767	0.78	0.01	0.3199	-0.53973
0.768	0.79	0.07	0.2889	-0.53832
0.770	0.84	0.13	0.2110	-0.48187
0.772	0.86	0.13	0.1812	-0.45580
0.774	0.88	0.08	0.2047	-0.42970
0.775	0.88	0.11	0.1574	-0.43579
0.777	0.87	0.17	0.1051	-0.44738
0.779	0.86	0.20	0.1146	-0.45098
0.781	0.85	0.20	0.1933	-0.44134
0.782	0.79	0.22	0.3431	-0.45829
0.784	0.75	0.22	0.4426	-0.44621
0.786	0.70	0.19	0.5441	-0.42207
0.788	0.66	0.11	0.6293	-0.39832
0.789	0.59	0.02	0.7100	-0.38343
0.791	0.38	-0.02	0.8618	-0.33746
0.793	0.26	0.00	0.9421	-0.20965
0.795	0.22	0.05	0.9643	-0.14034
0.796	0.27	-0.04	0.9560	-0.10905
0.798	0.23	0.02	0.9732	-0.03461
0.800	0.16	0.02	0.9854	0.04712
0.802	0.18	-0.05	0.9811	0.06762
0.804	0.19	-0.11	0.9713	0.07706
0.805	0.17	-0.11	0.9635	0.17271
0.807	0.11	-0.07	0.9685	0.21295
0.809	0.07	-0.04	0.9721	0.22163
0.811	0.17	0.12	0.9367	0.28195
0.812	0.24	0.02	0.9231	0.30336
0.814	0.23	-0.03	0.9389	0.25183

0.816	0.23	0.01	0.9320	0.28401
0.818	0.29	0.03	0.8931	0.33951
0.819	0.36	0.04	0.8851	0.28909
0.821	0.39	0.08	0.8969	0.19814
0.823	0.34	0.10	0.9054	0.23387
0.825	0.36	0.16	0.9106	0.11838
0.826	0.33	0.16	0.9288	0.06666
0.828	0.27	0.12	0.9547	0.01204
0.830	0.28	0.11	0.9537	-0.00477
0.832	0.30	0.21	0.9313	-0.04212
0.833	0.32	0.30	0.8914	-0.12345
0.835	0.34	0.23	0.8814	-0.23168
0.837	0.36	0.11	0.8826	-0.28078
0.839	0.32	0.08	0.9012	-0.27921
0.840	0.39	0.12	0.8691	-0.27851
0.842	0.44	0.13	0.8455	-0.27436
0.844	0.45	0.13	0.8353	-0.28700
0.846	0.43	0.10	0.8443	-0.30205
0.847	0.45	0.06	0.8345	-0.30648
0.849	0.48	0.04	0.8134	-0.33045
0.851	0.51	0.01	0.7981	-0.31264
0.853	0.46	0.04	0.8445	-0.26435
0.854	0.44	0.03	0.8701	-0.22322
0.856	0.39	0.07	0.8830	-0.24467
0.858	0.38	0.14	0.8640	-0.29340
0.860	0.43	0.15	0.8390	-0.29558
0.861	0.47	0.11	0.8391	-0.25467
0.863	0.43	0.12	0.8615	-0.24919
0.865	0.35	0.16	0.8824	-0.27369
0.867	0.33	0.16	0.8511	-0.37863
0.868	0.40	0.17	0.8289	-0.34838
0.870	0.39	0.16	0.8568	-0.29772
0.872	0.26	0.15	0.9075	-0.29404
0.874	0.22	0.12	0.8990	-0.36057
0.875	0.33	0.10	0.8471	-0.40406
0.877	0.37	0.11	0.8803	-0.27707
0.879	0.31	0.14	0.9388	-0.06479
0.881	0.35	0.02	0.9349	-0.00656
0.882	0.44	-0.05	0.8948	0.04781

0.884	0.41	-0.09	0.9021	-0.09298
0.886	0.31	-0.18	0.9171	-0.17760
0.888	0.32	-0.25	0.9142	-0.01281
0.889	0.39	-0.28	0.8747	0.05874
0.891	0.36	-0.28	0.8882	-0.05467
0.893	0.27	-0.32	0.8952	-0.14796
0.895	0.45	-0.01	0.8918	-0.02277
0.896	0.39	-0.12	0.9117	-0.08047
0.898	0.34	-0.17	0.9122	-0.16334
0.900	0.35	-0.12	0.9062	-0.20200
0.902	0.35	-0.06	0.9208	-0.14941
0.904	0.37	-0.09	0.9240	-0.04729
0.905	0.39	-0.11	0.9142	-0.04990
0.907	0.41	-0.09	0.9018	-0.09889
0.909	0.34	-0.14	0.9267	-0.07193
0.911	0.41	0.01	0.8863	-0.22441
0.912	0.37	0.08	0.8884	-0.26116
0.914	0.32	-0.04	0.9189	-0.22234
0.916	0.30	-0.16	0.8943	-0.29098
0.918	0.33	-0.08	0.8923	-0.29421
0.919	0.39	-0.04	0.8894	-0.24128
0.921	0.43	-0.10	0.8448	-0.29437
0.923	0.47	-0.03	0.8632	-0.17107
0.925	0.37	-0.01	0.9167	-0.15840
0.926	0.40	-0.14	0.8931	-0.14185
0.928	0.47	-0.14	0.8560	-0.17344
0.930	0.38	0.07	0.8900	-0.24154
0.932	0.33	0.15	0.9199	-0.14191
0.933	0.39	-0.02	0.9221	-0.00226
0.935	0.41	-0.16	0.8987	0.00970
0.937	0.39	-0.34	0.8573	-0.02690
0.939	0.31	-0.25	0.9130	-0.10255
0.940	0.23	-0.11	0.9601	-0.10506
0.942	0.25	0.01	0.9625	-0.09492
0.944	0.27	-0.03	0.9593	-0.08143
0.946	0.25	-0.04	0.9643	-0.08652
0.947	0.21	-0.13	0.9625	-0.10915
0.949	0.22	-0.18	0.9601	-0.01440
0.951	0.23	-0.17	0.9253	-0.25534

0.953	0.28	0.04	0.8842	-0.36967
0.954	0.26	0.02	0.9015	-0.34543
0.956	0.16	-0.10	0.9778	-0.08374
0.958	0.05	-0.13	0.9904	0.01548
0.960	0.02	-0.25	0.9573	-0.14908
0.961	-0.01	-0.42	0.8626	-0.28642
0.963	-0.17	-0.46	0.8200	-0.28704
0.965	-0.27	-0.77	0.5737	-0.07664
0.967	-0.54	-0.81	0.2230	0.02638
0.968	-0.48	-0.80	0.3021	0.19758
0.970	-0.54	-0.75	0.3045	0.23963
0.972	-0.71	-0.70	0.0433	0.06227
0.974	-0.74	-0.67	-0.0088	-0.04721
0.975	-0.68	-0.73	0.0395	-0.05747
0.977	-0.64	-0.76	-0.0862	-0.03770
0.979	-0.61	-0.79	-0.0928	0.05589
0.981	-0.62	-0.76	-0.1920	0.00986
0.982	-0.57	-0.79	-0.2264	-0.04513
0.984	-0.55	-0.81	-0.2242	0.03850
0.986	-0.58	-0.77	-0.2033	0.16615
0.988	-0.49	-0.83	-0.0788	0.23598
0.989	-0.43	-0.88	-0.0273	0.18435
0.991	-0.54	-0.80	-0.2197	0.11469
0.000	0.21	0.86	0.4496	0.13263
0.002	0.18	0.84	0.5208	0.00463
0.004	0.47	0.76	0.4452	-0.09220
0.005	0.24	0.70	0.6627	0.10889
0.007	0.14	0.85	0.5083	-0.08504
0.009	0.08	0.86	0.4812	-0.14124
0.011	0.05	0.73	0.6822	-0.04680
0.012	0.01	0.65	0.7403	-0.15390
0.014	-0.08	0.64	0.7383	-0.20267
0.016	-0.14	0.63	0.7510	-0.16253
0.018	-0.21	0.61	0.7401	-0.18314
0.019	-0.24	0.61	0.7100	-0.24965
0.021	-0.24	0.60	0.6952	-0.31739
0.023	-0.25	0.50	0.7015	-0.44208
0.025	-0.29	0.20	0.6619	-0.66276
0.026	-0.35	-0.16	0.5751	-0.71934
0.028	-0.60	-0.44	0.3786	-0.54531
0.030	-0.83	-0.31	0.1601	-0.42760
0.032	-0.85	0.05	0.5133	-0.11030

0.033	-0.71	0.39	0.5894	0.00390
0.035	-0.51	0.59	0.6261	0.06222
0.037	-0.39	0.65	0.6459	0.08157
0.039	-0.31	0.70	0.6367	0.08270
0.040	-0.26	0.75	0.5991	0.08600
0.042	-0.16	0.80	0.5710	0.10796
0.044	-0.10	0.80	0.5835	0.11762
0.046	-0.10	0.81	0.5709	0.06627
0.047	-0.08	0.81	0.5812	0.03332
0.049	-0.03	0.83	0.5508	0.01805
0.051	0.02	0.85	0.5235	0.04046
0.053	0.04	0.86	0.4976	0.07232
0.054	0.05	0.87	0.4881	0.08833
0.056	0.10	0.90	0.4223	0.08197
0.058	0.20	0.93	0.2924	0.07523
0.060	0.11	0.94	0.3100	0.09265
0.061	0.13	0.96	0.2297	0.00705
0.063	0.12	0.96	0.2530	-0.04847
0.065	0.06	0.95	0.3027	-0.02533
0.067	0.07	0.97	0.2187	-0.01092
0.068	0.06	0.95	0.2384	-0.17420
0.070	-0.03	0.84	0.3857	-0.37950
0.072	-0.17	0.78	0.4623	-0.37759
0.074	-0.18	0.83	0.5170	-0.13067
0.075	-0.25	0.83	0.4768	-0.15141
0.077	-0.21	0.86	0.3887	-0.23894
0.079	-0.08	0.92	0.3276	-0.21823
0.081	-0.11	0.96	0.2385	-0.08667
0.082	-0.29	0.94	0.1452	0.11121
0.084	-0.41	0.88	0.1098	0.22106
0.086	-0.43	0.85	0.1364	0.25989
0.088	-0.40	0.86	0.0412	0.32167
0.089	-0.46	0.84	0.0299	0.29608
0.091	-0.44	0.80	-0.0518	0.39284
0.093	-0.41	0.82	-0.1336	0.37060
0.095	-0.46	0.84	-0.1329	0.25186
0.096	-0.48	0.84	-0.1277	0.22481
0.098	-0.45	0.84	-0.1051	0.28718
0.100	-0.42	0.87	-0.0820	0.24905
0.102	-0.40	0.90	0.0670	0.16780
0.104	-0.40	0.89	0.0437	0.23157
0.105	-0.36	0.90	0.0428	0.24010
0.107	-0.39	0.89	0.1121	0.19760
0.109	-0.43	0.86	0.2091	0.17759
0.111	-0.42	0.86	0.2442	0.14036
0.112	-0.42	0.87	0.2510	0.08620
0.114	-0.45	0.85	0.2538	0.06015

0.116	-0.38	0.85	0.3662	-0.07018
0.118	-0.42	0.83	0.3762	-0.04560
0.119	-0.46	0.81	0.3626	-0.03339
0.121	-0.49	0.81	0.3127	-0.05119
0.123	-0.51	0.83	0.2030	-0.06784
0.125	-0.52	0.83	0.1432	-0.11835
0.126	-0.49	0.84	0.1347	-0.19505
0.128	-0.44	0.85	0.1464	-0.25682
0.130	-0.45	0.84	0.1165	-0.27804
0.132	-0.39	0.87	0.1299	-0.26965
0.133	-0.39	0.87	0.1564	-0.26931
0.135	-0.37	0.85	0.2444	-0.27248
0.137	-0.31	0.85	0.3101	-0.27487
0.139	-0.39	0.86	0.2455	-0.23024
0.140	-0.43	0.85	0.1753	-0.25842
0.142	-0.24	0.86	0.2394	-0.38672
0.144	0.03	0.75	0.3536	-0.55857
0.146	0.04	0.74	0.4983	-0.45192
0.147	0.34	0.70	0.5367	-0.33734
0.149	0.51	0.63	0.5080	-0.29760
0.151	0.40	0.65	0.5402	-0.35094
0.153	0.46	0.66	0.5534	-0.20314
0.154	0.59	0.48	0.6443	0.08321
0.156	0.49	0.21	0.7651	0.36597
0.158	0.43	0.37	0.8142	0.13076
0.160	0.32	0.55	0.7729	0.04396
0.161	0.25	0.67	0.6956	0.05919
0.163	0.26	0.73	0.6139	0.13199
0.165	0.32	0.69	0.6277	0.19216
0.167	0.19	0.60	0.7728	0.07793
0.168	-0.10	0.55	0.8251	-0.09363
0.170	-0.32	0.44	0.8070	-0.23275
0.172	-0.35	0.45	0.8203	0.05341
0.174	-0.55	0.47	0.6874	0.01809
0.175	-0.61	0.40	0.6550	-0.19561
0.177	-0.58	0.34	0.6859	-0.27557
0.179	-0.58	0.39	0.6765	-0.23132
0.181	-0.61	0.26	0.6826	-0.30095
0.182	-0.62	0.13	0.7439	-0.22041
0.184	-0.67	0.11	0.7299	0.10082
0.186	-0.64	0.43	0.4883	0.39743
0.188	-0.55	0.16	0.5946	0.56041
0.189	-0.41	0.29	0.5568	0.66040
0.191	-0.38	0.59	0.3662	0.61568
0.193	-0.50	0.63	0.0468	0.58607
0.195	-0.44	0.68	-0.0657	0.58260
0.196	-0.22	0.79	-0.0292	0.56879

0.198	-0.11	0.78	0.0853	0.60497
0.200	-0.53	0.77	0.1061	0.34022
0.202	-0.56	0.79	0.0330	0.24079
0.204	-0.68	0.71	0.0465	0.15291
0.205	-0.80	0.59	0.0758	0.07796
0.207	-0.85	0.50	-0.0133	0.12931
0.209	-0.80	0.52	-0.1916	0.23977
0.211	-0.67	0.71	-0.1200	0.17190
0.212	-0.57	0.82	0.1124	-0.02582
0.214	-0.62	0.67	-0.1103	0.38454
0.216	-0.72	0.62	-0.1635	0.25837
0.218	-0.66	0.75	-0.0341	-0.00355
0.219	-0.45	0.86	0.0565	-0.23857
0.221	-0.38	0.86	0.0997	-0.31947
0.223	-0.74	0.66	-0.0998	-0.03385
0.225	-0.76	0.52	-0.3222	0.21870
0.226	-0.56	0.54	-0.5397	0.32764
0.228	-0.54	0.23	-0.3209	0.74022
0.230	-0.64	0.56	-0.1363	0.50092
0.232	-0.62	0.78	0.0630	-0.06165
0.233	-0.62	0.77	-0.1427	-0.07431
0.235	-0.56	0.74	-0.3178	0.17812
0.237	-0.34	0.72	-0.3969	0.46701
0.239	-0.36	0.70	-0.4138	0.46128
0.240	-0.43	0.70	-0.4044	0.41322
0.242	-0.34	0.71	-0.3038	0.54346
0.244	-0.46	0.71	-0.1512	0.51057
0.246	-0.40	0.76	-0.1476	0.48624
0.247	-0.25	0.79	-0.1694	0.53792
0.249	-0.25	0.73	-0.1709	0.60927
0.251	-0.25	0.70	-0.2943	0.60091
0.253	-0.18	0.59	-0.3527	0.70332
0.254	-0.13	0.42	-0.1655	0.88406
0.256	-0.20	0.50	-0.0084	0.84323
0.258	-0.23	0.57	-0.0758	0.78978
0.260	-0.37	0.53	-0.0685	0.75817
0.261	-0.54	0.32	0.0399	0.77765
0.263	-0.63	0.05	0.1395	0.76470
0.265	-0.60	-0.12	0.2208	0.76013
0.267	-0.62	-0.06	0.2179	0.75293
0.268	-0.73	0.08	0.0377	0.68032
0.270	-0.91	-0.22	0.2192	0.26459
0.272	-0.83	-0.05	0.5107	0.20698
0.274	-0.72	0.11	0.6688	0.13852
0.275	-0.81	-0.01	0.5751	0.09500
0.277	-0.80	-0.21	0.4550	-0.32507
0.279	-0.62	-0.23	0.3503	-0.65782

0.281	-0.65	-0.17	0.5065	-0.54021
0.282	-0.68	-0.22	0.6955	0.09088
0.284	-0.52	0.08	0.8060	0.27882
0.286	-0.75	0.02	0.6542	0.11774
0.288	-0.75	0.28	0.5966	0.04179
0.289	-0.40	0.57	0.6756	0.23263
0.291	-0.38	0.45	0.7858	0.18562
0.293	-0.54	0.07	0.8336	0.10305
0.295	-0.60	-0.02	0.7713	0.20351
0.296	-0.44	0.04	0.7636	0.47053
0.298	-0.16	-0.15	0.9681	0.12040
0.300	-0.09	-0.38	0.9107	-0.14367
0.302	-0.35	-0.41	0.7763	-0.33559
0.304	-0.48	-0.31	0.7475	-0.34361
0.305	-0.40	-0.23	0.7710	-0.43555
0.307	-0.37	-0.18	0.6968	-0.58799
0.309	-0.34	-0.19	0.5647	-0.72818
0.311	-0.24	-0.18	0.6235	-0.72086
0.312	-0.26	0.09	0.7159	-0.64201
0.314	-0.05	0.20	0.7436	-0.63715
0.316	0.32	0.42	0.7048	-0.47244
0.318	0.54	0.50	0.5973	-0.30601
0.319	0.70	0.46	0.5387	-0.07702
0.321	0.70	0.40	0.5754	0.14568
0.323	0.64	0.41	0.5579	0.33088
0.325	0.55	0.43	0.5853	0.41493
0.326	0.53	0.32	0.5575	0.54726
0.328	0.50	0.28	0.5742	0.58416
0.330	0.50	0.26	0.5779	0.58813
0.332	0.46	0.28	0.5949	0.59893
0.333	0.39	0.23	0.6468	0.61424
0.335	0.41	0.14	0.6607	0.61335
0.337	0.43	0.02	0.6918	0.58086
0.339	0.35	-0.04	0.7626	0.54170
0.340	0.24	-0.14	0.7764	0.56737
0.342	0.12	-0.12	0.7933	0.58359
0.344	0.10	-0.22	0.7859	0.56933
0.346	0.30	-0.39	0.7440	0.45388
0.347	0.34	-0.45	0.6830	0.46528
0.349	0.24	-0.43	0.7560	0.42398
0.351	0.24	-0.53	0.7853	0.20914
0.353	0.35	-0.47	0.8111	0.01685
0.354	0.23	-0.54	0.6386	0.50302
0.356	0.29	-0.37	0.6744	0.56993
0.358	0.40	-0.24	0.6920	0.54940
0.360	0.47	-0.25	0.6620	0.52151
0.361	0.42	-0.31	0.7044	0.47819

0.363	0.27	-0.26	0.8229	0.42589
0.365	0.44	-0.21	0.7359	0.47194
0.367	0.64	-0.15	0.5687	0.49224
0.368	0.72	0.04	0.5815	0.37444
0.370	0.82	0.01	0.5129	0.25622
0.372	0.79	0.00	0.5494	0.25767
0.374	0.77	-0.02	0.5948	0.22837
0.375	0.89	-0.13	0.4287	0.08183
0.377	0.94	-0.11	0.3069	0.11284
0.379	0.92	-0.01	0.3164	0.22292
0.381	0.92	0.07	0.3421	0.17991
0.382	0.94	0.12	0.2812	0.17496
0.384	0.97	0.08	0.1535	0.14828
0.386	0.97	0.06	0.1674	0.14219
0.388	0.97	0.08	0.2081	0.12931
0.389	0.97	0.10	0.1435	0.15431
0.391	0.97	0.12	0.1294	0.15994
0.393	0.97	0.09	0.1604	0.16346
0.395	0.97	0.03	0.1593	0.15379
0.396	0.95	-0.00	0.2789	0.12515
0.398	0.94	0.11	0.2131	0.23571
0.400	0.92	0.16	0.2099	0.29035
0.402	0.92	0.13	0.3040	0.22584
0.404	0.87	0.09	0.4583	0.15422
0.405	0.82	0.12	0.5308	0.16421
0.407	0.83	0.13	0.5282	0.12612
0.409	0.86	0.11	0.4975	0.03180
0.411	0.80	-0.00	0.5607	-0.19712
0.412	0.78	0.02	0.5845	-0.23222
0.414	0.75	0.01	0.6310	-0.17850
0.416	0.70	-0.05	0.7025	-0.10848
0.418	0.62	-0.07	0.7677	-0.15657
0.419	0.60	-0.05	0.7904	-0.10939
0.421	0.58	-0.07	0.8134	0.00774
0.423	0.52	-0.09	0.8445	0.08340
0.425	0.53	-0.03	0.8416	0.12204
0.426	0.47	0.02	0.8520	0.22186
0.428	0.44	0.05	0.8554	0.26570
0.430	0.41	0.06	0.8573	0.30033
0.432	0.40	0.09	0.8396	0.35934
0.433	0.40	0.12	0.8115	0.40505
0.435	0.41	0.12	0.8044	0.41739
0.437	0.40	0.10	0.8162	0.40750
0.439	0.36	0.10	0.8309	0.40976
0.440	0.41	0.08	0.8137	0.40867
0.442	0.45	0.06	0.7917	0.40825
0.444	0.48	0.04	0.7746	0.40925

0.446	0.51	-0.01	0.7585	0.40009
0.447	0.57	-0.08	0.7282	0.36729
0.449	0.62	-0.14	0.6892	0.34296
0.451	0.66	-0.18	0.6502	0.32613
0.453	0.72	-0.27	0.5834	0.24876
0.454	0.74	-0.34	0.5342	0.23951
0.456	0.77	-0.37	0.4684	0.23440
0.458	0.80	-0.37	0.4291	0.19595
0.460	0.80	-0.38	0.4320	0.14904
0.461	0.79	-0.40	0.4308	0.15026
0.463	0.80	-0.37	0.4499	0.16463
0.465	0.81	-0.29	0.4904	0.15696
0.467	0.76	-0.26	0.5776	0.12045
0.468	0.74	-0.25	0.6111	0.10970
0.470	0.73	-0.20	0.6475	0.08902
0.472	0.73	-0.12	0.6636	0.08251
0.474	0.74	-0.07	0.6635	0.07279
0.475	0.74	-0.05	0.6711	0.05580
0.477	0.73	-0.04	0.6791	0.00707
0.479	0.74	-0.00	0.6764	-0.03881
0.481	0.76	0.04	0.6429	-0.09189
0.482	0.76	0.08	0.6298	-0.12196
0.484	0.77	0.10	0.6162	-0.12189
0.486	0.78	0.10	0.6092	-0.09531
0.488	0.80	0.08	0.5892	-0.09951
0.489	0.80	0.08	0.5893	-0.11568
0.491	0.81	0.08	0.5663	-0.13588
0.493	0.82	0.11	0.5410	-0.14259
0.495	0.85	0.14	0.4804	-0.16881
0.496	0.85	0.18	0.4568	-0.19707
0.498	0.84	0.18	0.4483	-0.25130
0.500	0.86	0.16	0.4165	-0.26450
0.502	0.88	0.14	0.3768	-0.24289
0.504	0.90	0.14	0.3446	-0.23263
0.505	0.90	0.15	0.3249	-0.24942
0.507	0.91	0.17	0.2941	-0.23726
0.509	0.93	0.13	0.2873	-0.20533
0.511	0.92	0.12	0.3145	-0.17772
0.512	0.93	0.11	0.3176	-0.13885
0.514	0.94	0.11	0.2912	-0.11691
0.516	0.95	0.11	0.2703	-0.09437
0.518	0.95	0.12	0.2685	-0.07348
0.519	0.96	0.12	0.2554	-0.04919
0.521	0.97	0.11	0.2198	-0.03228
0.523	0.97	0.13	0.1829	0.01061
0.525	0.98	0.10	0.1797	0.03529
0.526	0.98	0.09	0.1812	0.05963

0.528	0.98	0.11	0.1780	0.07289
0.530	0.97	0.14	0.1597	0.06954
0.532	0.97	0.14	0.1653	0.06693
0.533	0.97	0.15	0.1602	0.07762
0.535	0.97	0.15	0.1742	0.07854
0.537	0.96	0.16	0.2044	0.08350
0.539	0.96	0.17	0.1919	0.08966
0.540	0.96	0.18	0.1711	0.11688
0.542	0.96	0.20	0.1692	0.13005
0.544	0.95	0.22	0.1770	0.10550
0.546	0.94	0.24	0.2185	0.07877
0.547	0.93	0.25	0.2485	0.09309
0.549	0.93	0.25	0.2452	0.11266
0.551	0.95	0.23	0.2079	0.09815
0.553	0.93	0.24	0.2716	0.11458
0.554	0.92	0.25	0.2985	0.09508
0.556	0.92	0.25	0.2771	0.07800
0.558	0.93	0.25	0.2498	0.05317
0.560	0.93	0.26	0.2657	0.03890
0.561	0.92	0.29	0.2763	0.00175
0.563	0.92	0.30	0.2578	-0.03319
0.565	0.91	0.30	0.2609	0.06879
0.567	0.92	0.26	0.2684	0.10890
0.568	0.91	0.25	0.2845	0.16003
0.570	0.90	0.27	0.3088	0.17032
0.572	0.87	0.30	0.3383	0.19582
0.574	0.86	0.30	0.3458	0.21569
0.575	0.87	0.29	0.3272	0.22278
0.577	0.89	0.29	0.2898	0.20816
0.579	0.89	0.28	0.2747	0.23746
0.581	0.88	0.26	0.3315	0.23063
0.582	0.87	0.25	0.3729	0.20599
0.584	0.88	0.25	0.3568	0.20673
0.586	0.87	0.26	0.3396	0.24116
0.588	0.85	0.24	0.3594	0.29445
0.589	0.83	0.25	0.3954	0.29895
0.591	0.83	0.26	0.4164	0.26696
0.593	0.80	0.21	0.4629	0.31629
0.595	0.81	0.19	0.4837	0.27342
0.596	0.83	0.18	0.4301	0.29464
0.598	0.87	0.18	0.3650	0.29221
0.600	0.87	0.16	0.4014	0.24847
0.602	0.84	0.14	0.4521	0.26079
0.604	0.84	0.15	0.4259	0.30720
0.605	0.86	0.17	0.3849	0.28459
0.607	0.85	0.12	0.4361	0.27798
0.609	0.82	0.15	0.4606	0.29054

0.611	0.81	0.19	0.4895	0.27047
0.612	0.82	0.19	0.4837	0.23498
0.614	0.85	0.18	0.4517	0.20211
0.616	0.87	0.16	0.4216	0.21761
0.618	0.88	0.13	0.4048	0.19745
0.619	0.89	0.10	0.4157	0.15455
0.621	0.93	0.12	0.3218	0.10577
0.623	0.93	0.13	0.3332	0.09488
0.625	0.93	0.11	0.3322	0.08385
0.626	0.95	0.09	0.2858	0.11572
0.628	0.97	0.07	0.2002	0.13971
0.630	0.98	0.04	0.1716	0.11038
0.632	0.98	0.04	0.1794	0.06528
0.633	0.97	0.07	0.2095	0.04310
0.635	0.99	0.07	0.1367	-0.02025
0.637	0.97	0.10	0.2132	-0.03356
0.639	0.97	0.07	0.2477	-0.01321
0.640	0.97	0.01	0.2376	0.01033
0.642	0.97	-0.01	0.2341	0.02085
0.644	0.96	-0.01	0.2816	0.02503
0.646	0.95	-0.06	0.3014	0.07895
0.647	0.94	-0.13	0.2917	0.13689
0.649	0.94	-0.11	0.2876	0.14648
0.651	0.93	-0.13	0.3072	0.16612
0.653	0.91	-0.15	0.3428	0.19907
0.654	0.88	-0.14	0.3815	0.25310
0.656	0.87	-0.10	0.3997	0.26273
0.658	0.86	-0.07	0.4283	0.25476
0.660	0.84	-0.05	0.4740	0.26036
0.661	0.80	-0.03	0.5188	0.28745
0.663	0.78	0.05	0.5501	0.29962
0.665	0.77	0.07	0.5505	0.31787
0.667	0.74	0.08	0.5818	0.33547
0.668	0.73	0.11	0.5869	0.33899
0.670	0.73	0.13	0.5728	0.35767
0.672	0.71	0.17	0.5822	0.35818
0.674	0.68	0.21	0.6144	0.33703
0.675	0.67	0.25	0.6129	0.32367
0.677	0.71	0.24	0.5632	0.34189
0.679	0.73	0.24	0.5501	0.31760
0.681	0.76	0.24	0.5377	0.28118
0.682	0.74	0.27	0.5653	0.24675
0.684	0.70	0.29	0.6159	0.22378
0.686	0.70	0.29	0.6262	0.18015
0.688	0.69	0.26	0.6540	0.15556
0.689	0.65	0.21	0.7196	0.15161
0.691	0.56	0.24	0.7837	0.13384

0.693	0.57	0.21	0.7862	0.10862
0.695	0.63	0.14	0.7537	0.10781
0.696	0.68	0.11	0.7211	0.06173
0.698	0.68	0.17	0.7136	0.00677
0.700	0.67	0.24	0.7016	0.00810
0.702	0.73	0.25	0.6293	-0.02416
0.704	0.80	0.23	0.5467	-0.10930
0.705	0.81	0.29	0.4922	-0.12467
0.707	0.83	0.29	0.4519	-0.14918
0.709	0.83	0.31	0.4233	-0.18800
0.711	0.84	0.31	0.4049	-0.20191
0.712	0.86	0.28	0.3777	-0.17207
0.714	0.89	0.24	0.3593	-0.13732
0.716	0.90	0.21	0.3599	-0.14991
0.718	0.89	0.20	0.3636	-0.18312
0.719	0.88	0.17	0.3751	-0.21560
0.721	0.90	0.21	0.3542	-0.15549
0.723	0.90	0.25	0.3366	-0.12376
0.725	0.91	0.24	0.3160	-0.11981
0.726	0.92	0.21	0.2877	-0.14111
0.728	0.92	0.20	0.2935	-0.14983
0.730	0.93	0.17	0.3026	-0.13688
0.732	0.95	0.12	0.2543	-0.10488
0.733	0.96	0.11	0.2252	-0.07862
0.735	0.97	0.12	0.2023	-0.07568
0.737	0.96	0.11	0.2593	-0.04676
0.739	0.95	0.11	0.2874	-0.00169
0.740	0.96	0.13	0.2584	0.01353
0.742	0.96	0.11	0.2485	-0.00850
0.744	0.95	0.08	0.2980	-0.00714
0.746	0.94	0.07	0.3290	0.02809
0.747	0.95	0.04	0.3031	0.09132
0.749	0.95	0.03	0.2793	0.10151
0.751	0.95	0.01	0.2907	0.10343
0.753	0.94	-0.01	0.3357	0.10151
0.754	0.92	-0.01	0.3676	0.11639
0.756	0.92	0.00	0.3594	0.14310
0.758	0.91	-0.01	0.3689	0.17689
0.760	0.90	-0.03	0.3963	0.19141
0.761	0.89	-0.02	0.4233	0.16574
0.763	0.88	-0.04	0.4152	0.20709
0.765	0.86	-0.07	0.4412	0.24195
0.767	0.83	-0.09	0.4923	0.24388
0.768	0.82	-0.08	0.5199	0.22835
0.770	0.83	-0.05	0.5054	0.22293
0.772	0.84	-0.03	0.4813	0.23397
0.774	0.84	-0.03	0.4794	0.24540

0.775	0.83	-0.04	0.5057	0.23832
0.777	0.83	-0.04	0.5140	0.21878
0.779	0.84	-0.04	0.5096	0.18058
0.781	0.87	-0.04	0.4734	0.13679
0.782	0.89	-0.04	0.4426	0.11308
0.784	0.89	-0.02	0.4322	0.11964
0.786	0.89	0.01	0.4340	0.12111
0.788	0.89	0.03	0.4348	0.11191
0.789	0.94	0.02	0.3368	0.08077
0.791	0.94	0.02	0.3205	0.06439
0.793	0.95	0.02	0.3180	0.00852
0.795	0.94	0.04	0.3375	-0.00894
0.796	0.93	0.06	0.3597	-0.01372
0.798	0.94	0.09	0.3280	-0.04615
0.800	0.94	0.11	0.3182	-0.09699
0.802	0.92	0.12	0.3713	-0.09743
0.804	0.88	0.14	0.4018	-0.22126
0.805	0.86	0.16	0.4095	-0.25902
0.807	0.85	0.16	0.4147	-0.29099
0.809	0.84	0.15	0.4257	-0.30621
0.811	0.82	0.18	0.4564	-0.30521
0.812	0.79	0.22	0.4737	-0.32271
0.814	0.79	0.23	0.4368	-0.35779
0.816	0.81	0.21	0.4021	-0.37308
0.818	0.82	0.25	0.3585	-0.36807
0.819	0.86	0.20	0.3200	-0.34677
0.821	0.88	0.22	0.2828	-0.30445
0.823	0.87	0.30	0.2427	-0.29225
0.825	0.86	0.32	0.2293	-0.33482
0.826	0.85	0.27	0.2850	-0.34095
0.828	0.86	0.27	0.3193	-0.27825
0.830	0.88	0.30	0.2869	-0.22930
0.832	0.90	0.28	0.2714	-0.20437
0.833	0.90	0.28	0.2780	-0.19729
0.835	0.90	0.29	0.2868	-0.17315
0.837	0.88	0.32	0.3066	-0.14532
0.839	0.88	0.32	0.3218	-0.11882
0.840	0.89	0.30	0.3300	-0.08365
0.842	0.89	0.30	0.3572	-0.03892
0.844	0.88	0.30	0.3761	0.00013
0.846	0.85	0.32	0.3999	0.07511
0.847	0.87	0.32	0.3781	0.06955
0.849	0.88	0.31	0.3467	0.07063
0.851	0.88	0.33	0.3235	0.07629
0.853	0.87	0.35	0.3417	0.07128
0.854	0.86	0.37	0.3558	0.05351
0.856	0.86	0.37	0.3557	0.04610

0.858	0.86	0.37	0.3423	0.04965
0.860	0.85	0.41	0.3218	0.06385
0.861	0.83	0.41	0.3658	0.07456
0.863	0.82	0.41	0.3888	0.06752
0.865	0.82	0.42	0.3885	0.04356
0.867	0.82	0.43	0.3880	0.02640
0.868	0.81	0.42	0.4047	0.03088
0.870	0.81	0.41	0.4249	0.03884
0.872	0.81	0.40	0.4298	0.04061
0.874	0.81	0.40	0.4201	0.10494
0.875	0.79	0.42	0.4323	0.10883
0.877	0.79	0.42	0.4445	0.09703
0.879	0.79	0.41	0.4541	0.07911
0.881	0.79	0.40	0.4537	0.08167
0.882	0.79	0.41	0.4497	0.09023
0.884	0.78	0.42	0.4536	0.09980
0.886	0.77	0.42	0.4737	0.11509
0.888	0.75	0.40	0.5126	0.07646
0.889	0.75	0.41	0.5150	0.10480
0.891	0.74	0.42	0.5094	0.10443
0.893	0.74	0.43	0.5046	0.06024
0.895	0.75	0.45	0.4831	0.05331
0.896	0.75	0.45	0.4709	0.09417
0.898	0.76	0.41	0.4839	0.13491
0.900	0.76	0.37	0.5141	0.13022
0.902	0.75	0.35	0.5433	0.13932
0.904	0.75	0.29	0.5512	0.20172
0.905	0.76	0.25	0.5678	0.18478
0.907	0.77	0.23	0.5784	0.14830
0.909	0.78	0.20	0.5699	0.16626
0.911	0.79	0.17	0.5565	0.18562
0.912	0.80	0.16	0.5473	0.19626
0.914	0.80	0.15	0.5310	0.24786
0.916	0.80	0.09	0.5417	0.23498
0.918	0.82	0.05	0.5021	0.26018
0.919	0.84	0.06	0.4751	0.25673
0.921	0.83	0.09	0.4953	0.23215
0.923	0.83	0.03	0.5152	0.22245
0.925	0.83	-0.09	0.4962	0.22543
0.926	0.84	-0.15	0.4798	0.22411
0.928	0.83	-0.12	0.5116	0.20467
0.930	0.85	-0.12	0.5012	0.11677
0.932	0.86	-0.11	0.4681	0.15662
0.933	0.87	-0.14	0.4473	0.16484
0.935	0.86	-0.18	0.4679	0.11895
0.937	0.85	-0.18	0.4882	0.10067
0.939	0.85	-0.16	0.4812	0.12757

0.940	0.85	-0.14	0.4850	0.14380
0.942	0.85	-0.14	0.4952	0.13673
0.944	0.79	-0.16	0.5924	0.01797
0.946	0.83	-0.10	0.5411	0.07479
0.947	0.82	-0.08	0.5506	0.12045
0.949	0.76	-0.08	0.6283	0.14395
0.951	0.69	-0.16	0.6994	0.08728
0.953	0.62	-0.25	0.7427	0.03093
0.954	0.36	-0.28	0.8885	0.06060
0.956	0.19	-0.23	0.9467	0.12917
0.958	0.21	-0.31	0.8939	0.25250
0.960	0.03	-0.45	0.8690	0.20346
0.961	-0.12	-0.55	0.8172	0.10889
0.963	-0.26	-0.51	0.8192	0.03993
0.965	-0.43	-0.50	0.7464	-0.01451
0.967	-0.52	-0.61	0.6017	-0.04536
0.968	-0.56	-0.75	0.3581	-0.06064
0.970	-0.61	-0.77	0.1750	-0.03677
0.972	-0.54	-0.82	0.1577	-0.11245
0.974	-0.56	-0.82	0.0102	-0.10306
0.975	-0.60	-0.78	-0.0738	-0.14719
0.977	-0.67	-0.73	-0.1039	-0.11325
0.979	-0.69	-0.71	-0.1361	0.02110
0.981	-0.72	-0.67	-0.1330	0.11134
0.982	-0.78	-0.58	-0.0781	0.22456

Table S3. Factor Loadings obtained from PCA with varimax rotation analysis based on data from HPLC chromatograms. (The highest contribution for values >0.9)

Variable / RT	Factor Loadings (Varimax normalized) Extraction: Principal components (Marked loadings are >.700000)			
	Factor1	Factor2	Factor3	Factor4
0.1	-0.50	0.12	-0.22	0.83
0.12	-0.37	0.07	-0.05	0.92
0.13	0.69	-0.23	0.60	-0.34
0.15	0.66	-0.22	0.49	-0.53
0.17	0.66	-0.23	0.47	-0.53
0.18	0.67	-0.25	0.48	-0.51
0.2	0.68	-0.28	0.49	-0.47
0.22	0.68	-0.31	0.51	-0.42
0.23	0.67	-0.33	0.55	-0.37
0.25	0.62	-0.36	0.63	-0.29
0.27	0.31	-0.38	0.87	-0.02
0.28	-0.77	0.10	0.20	0.60
0.3	-0.78	0.24	-0.20	0.54
0.32	-0.74	0.28	-0.33	0.52
0.33	-0.71	0.29	-0.40	0.51
0.35	-0.67	0.29	-0.46	0.50
0.37	-0.64	0.29	-0.51	0.50
0.38	-0.61	0.29	-0.55	0.49
0.4	-0.58	0.28	-0.60	0.48
0.42	-0.56	0.27	-0.63	0.47

0.43	-0.54	0.25	-0.66	0.46
0.45	-0.54	0.22	-0.65	0.49
0.47	-0.55	0.18	-0.60	0.56
0.48	-0.55	0.13	-0.48	0.67
0.5	-0.54	0.07	-0.34	0.76
0.52	-0.53	0.03	-0.23	0.82
0.53	-0.53	0.01	-0.15	0.84
0.55	-0.54	0.00	-0.10	0.83
0.57	-0.57	0.03	-0.04	0.82
0.58	-0.58	0.08	0.04	0.81
0.6	-0.58	0.17	0.14	0.79
0.62	-0.53	0.29	0.23	0.76
0.63	-0.45	0.41	0.30	0.73
0.65	-0.33	0.53	0.33	0.71
0.67	-0.17	0.64	0.29	0.69
0.68	0.06	0.70	0.22	0.68
0.7	0.37	0.67	0.11	0.64
0.72	0.65	0.55	0.03	0.52
0.73	0.85	0.43	0.01	0.31

0.75	0.93	0.33	0.16	-0.04
0.77	0.85	0.25	0.40	-0.25
0.78	0.77	0.27	0.54	-0.21
0.8	0.72	0.28	0.61	-0.17
0.82	0.68	0.30	0.66	-0.10
0.83	0.65	0.33	0.69	-0.03
0.85	0.61	0.35	0.71	0.02
0.87	0.55	0.36	0.75	0.07
0.88	0.45	0.33	0.83	0.08
0.9	0.23	0.25	0.94	0.05
0.92	-0.06	0.12	0.99	-0.03
0.93	-0.17	0.10	0.98	-0.06
0.95	-0.10	0.19	0.98	-0.03
0.97	0.12	0.30	0.94	0.07
0.98	0.37	0.35	0.84	0.19
1	0.52	0.33	0.75	0.25
1.02	0.62	0.26	0.68	0.29
1.03	0.69	0.18	0.63	0.30
1.05	0.76	0.10	0.57	0.29
1.07	0.82	0.04	0.52	0.24
1.08	0.87	0.01	0.46	0.18
1.1	0.91	0.01	0.40	0.09
1.12	0.94	0.05	0.33	0.02
1.13	0.96	0.09	0.27	-0.04
1.15	0.96	0.14	0.24	-0.08
1.17	0.95	0.19	0.24	-0.11
1.18	0.93	0.22	0.27	-0.13
1.2	0.92	0.22	0.28	-0.15
1.22	0.93	0.19	0.27	-0.16
1.23	0.95	0.15	0.23	-0.16
1.25	0.96	0.12	0.19	-0.17
1.27	0.97	0.10	0.15	-0.17
1.28	0.97	0.08	0.13	-0.16
1.3	0.98	0.08	0.12	-0.15
1.32	0.98	0.09	0.14	-0.12
1.33	0.97	0.11	0.19	-0.08
1.35	0.96	0.13	0.25	-0.04
1.37	0.94	0.16	0.31	-0.01
1.38	0.91	0.18	0.37	0.01
1.4	0.89	0.20	0.41	0.01
1.42	0.87	0.21	0.44	0.01
1.43	0.86	0.21	0.47	0.01
1.45	0.85	0.21	0.49	0.02
1.47	0.84	0.20	0.50	0.02
1.48	0.83	0.20	0.52	0.02
1.5	0.83	0.19	0.53	0.02
1.52	0.82	0.18	0.54	0.01

1.53	0.82	0.18	0.54	0.01
1.55	0.81	0.18	0.55	0.00
1.57	0.80	0.18	0.57	0.00
1.58	0.79	0.18	0.59	0.00
1.6	0.77	0.18	0.61	0.00
1.62	0.74	0.18	0.64	0.01
1.63	0.72	0.18	0.67	0.01
1.65	0.70	0.18	0.69	0.02
1.67	0.69	0.18	0.70	0.03
1.68	0.69	0.19	0.70	0.04
1.7	0.71	0.20	0.68	0.04
1.72	0.73	0.20	0.65	0.03
1.73	0.76	0.21	0.61	0.02
1.75	0.79	0.21	0.57	0.00
1.77	0.82	0.21	0.53	-0.01
1.78	0.84	0.21	0.49	-0.03
1.8	0.86	0.21	0.46	-0.05
1.82	0.87	0.21	0.43	-0.06
1.83	0.88	0.21	0.41	-0.07
1.85	0.89	0.22	0.39	-0.08
1.87	0.90	0.22	0.37	-0.09
1.88	0.90	0.22	0.36	-0.09
1.9	0.91	0.22	0.35	-0.10
1.92	0.91	0.22	0.34	-0.10
1.93	0.91	0.23	0.33	-0.10
1.95	0.91	0.24	0.33	-0.10
1.97	0.91	0.24	0.32	-0.10
1.98	0.91	0.25	0.33	-0.09
2	0.90	0.27	0.34	-0.09
2.02	0.89	0.28	0.35	-0.08
2.03	0.88	0.30	0.36	-0.07
2.05	0.87	0.31	0.38	-0.07
2.07	0.86	0.33	0.39	-0.06
2.08	0.85	0.35	0.40	-0.05
2.1	0.83	0.37	0.41	-0.05
2.12	0.82	0.38	0.41	-0.04
2.13	0.82	0.40	0.42	-0.04
2.15	0.81	0.41	0.42	-0.03
2.17	0.80	0.43	0.42	-0.03
2.18	0.80	0.44	0.41	-0.03
2.2	0.79	0.45	0.41	-0.03
2.22	0.79	0.46	0.41	-0.03
2.23	0.78	0.47	0.41	-0.03
2.25	0.77	0.48	0.42	-0.03
2.27	0.76	0.49	0.43	-0.03
2.28	0.75	0.50	0.43	-0.03
2.3	0.73	0.51	0.45	-0.03

2.32	0.72	0.53	0.46	-0.03
2.33	0.70	0.54	0.47	-0.02
2.35	0.68	0.55	0.48	-0.01
2.37	0.66	0.57	0.49	-0.01
2.38	0.64	0.58	0.50	0.00
2.4	0.62	0.60	0.51	0.01
2.42	0.60	0.61	0.51	0.03
2.43	0.58	0.63	0.52	0.04
2.45	0.57	0.64	0.52	0.05
2.47	0.56	0.65	0.52	0.06
2.48	0.55	0.66	0.51	0.07
2.5	0.55	0.66	0.50	0.09
2.52	0.56	0.66	0.49	0.10
2.53	0.57	0.65	0.48	0.11
2.55	0.60	0.65	0.46	0.11
2.57	0.62	0.63	0.45	0.12
2.58	0.65	0.61	0.43	0.12
2.6	0.68	0.59	0.41	0.12
2.62	0.71	0.56	0.40	0.12
2.63	0.74	0.54	0.38	0.12
2.65	0.77	0.51	0.37	0.11
2.67	0.79	0.49	0.36	0.11
2.68	0.81	0.47	0.35	0.10
2.7	0.82	0.45	0.34	0.10
2.72	0.83	0.44	0.34	0.09
2.73	0.84	0.43	0.33	0.09
2.75	0.85	0.41	0.33	0.08
2.77	0.85	0.40	0.32	0.06
2.78	0.86	0.39	0.32	0.05
2.8	0.87	0.38	0.31	0.04
2.82	0.88	0.37	0.31	0.02
2.83	0.88	0.36	0.31	0.01
2.85	0.89	0.34	0.31	-0.01
2.87	0.89	0.33	0.31	-0.02
2.88	0.90	0.32	0.31	-0.03
2.9	0.90	0.30	0.32	-0.04
2.92	0.90	0.29	0.32	-0.05
2.93	0.90	0.27	0.33	-0.06
2.95	0.91	0.26	0.33	-0.07
2.97	0.91	0.24	0.33	-0.08
2.98	0.92	0.22	0.32	-0.09
3	0.92	0.21	0.31	-0.10
3.02	0.93	0.19	0.30	-0.11
3.03	0.93	0.18	0.29	-0.12
3.05	0.94	0.17	0.28	-0.12
3.07	0.94	0.16	0.27	-0.13
3.08	0.94	0.15	0.27	-0.14

3.1	0.94	0.14	0.26	-0.14
3.12	0.94	0.14	0.26	-0.15
3.13	0.94	0.13	0.26	-0.15
3.15	0.94	0.13	0.26	-0.15
3.17	0.94	0.13	0.27	-0.15
3.18	0.94	0.13	0.28	-0.15
3.2	0.94	0.12	0.29	-0.15
3.22	0.94	0.12	0.29	-0.15
3.23	0.93	0.12	0.30	-0.15
3.25	0.93	0.12	0.31	-0.15
3.27	0.93	0.12	0.31	-0.15
3.28	0.93	0.12	0.32	-0.15
3.3	0.93	0.12	0.32	-0.15
3.32	0.93	0.12	0.32	-0.14
3.33	0.93	0.13	0.33	-0.14
3.35	0.93	0.13	0.33	-0.13
3.37	0.92	0.14	0.33	-0.13
3.38	0.92	0.14	0.34	-0.12
3.4	0.92	0.15	0.34	-0.11
3.42	0.92	0.16	0.34	-0.10
3.43	0.92	0.16	0.35	-0.10
3.45	0.91	0.18	0.35	-0.09
3.47	0.91	0.19	0.36	-0.08
3.48	0.91	0.20	0.36	-0.08
3.5	0.90	0.22	0.36	-0.08
3.52	0.90	0.23	0.36	-0.07
3.53	0.90	0.25	0.36	-0.07
3.55	0.89	0.27	0.35	-0.07
3.57	0.89	0.29	0.34	-0.07
3.58	0.89	0.31	0.33	-0.06
3.6	0.89	0.33	0.31	-0.06
3.62	0.89	0.35	0.29	-0.06
3.63	0.89	0.37	0.27	-0.06
3.65	0.89	0.39	0.24	-0.05
3.67	0.89	0.40	0.22	-0.05
3.68	0.89	0.41	0.20	-0.05
3.7	0.89	0.42	0.18	-0.05
3.72	0.89	0.43	0.16	-0.05
3.73	0.88	0.44	0.15	-0.05
3.75	0.88	0.45	0.15	-0.05
3.77	0.88	0.46	0.14	-0.04
3.78	0.87	0.47	0.14	-0.04
3.8	0.87	0.48	0.14	-0.04
3.82	0.86	0.49	0.14	-0.04
3.83	0.85	0.50	0.15	-0.03
3.85	0.85	0.51	0.15	-0.03
3.87	0.84	0.52	0.15	-0.03

3.88	0.83	0.53	0.15	-0.03
3.9	0.83	0.54	0.15	-0.02
3.92	0.83	0.54	0.15	-0.02
3.93	0.82	0.55	0.14	-0.02
3.95	0.82	0.55	0.14	-0.02
3.97	0.83	0.55	0.13	-0.02
3.98	0.83	0.54	0.13	-0.02
4	0.83	0.54	0.12	-0.02
4.02	0.83	0.54	0.11	-0.02
4.03	0.84	0.54	0.11	-0.02
4.05	0.84	0.54	0.10	-0.02
4.07	0.84	0.54	0.10	-0.02
4.08	0.84	0.54	0.10	-0.01
4.1	0.83	0.54	0.10	-0.01
4.12	0.83	0.55	0.09	-0.00
4.13	0.83	0.55	0.09	0.00
4.15	0.82	0.56	0.09	0.01
4.17	0.82	0.57	0.09	0.01
4.18	0.81	0.58	0.09	0.02
4.2	0.80	0.59	0.09	0.03
4.22	0.79	0.60	0.09	0.04
4.23	0.78	0.61	0.09	0.05
4.25	0.77	0.63	0.09	0.06
4.27	0.75	0.65	0.10	0.07
4.28	0.73	0.67	0.10	0.09
4.3	0.70	0.70	0.11	0.11
4.32	0.67	0.72	0.13	0.13
4.33	0.64	0.74	0.14	0.15
4.35	0.61	0.76	0.15	0.17
4.37	0.57	0.78	0.16	0.19
4.38	0.54	0.80	0.17	0.21
4.4	0.51	0.81	0.18	0.22
4.42	0.48	0.82	0.19	0.24
4.43	0.45	0.83	0.19	0.25
4.45	0.43	0.84	0.19	0.26
4.47	0.41	0.85	0.19	0.27
4.48	0.39	0.86	0.19	0.27
4.5	0.38	0.87	0.18	0.27
4.52	0.36	0.87	0.17	0.28
4.53	0.35	0.88	0.17	0.28
4.55	0.34	0.89	0.16	0.28
4.57	0.32	0.89	0.15	0.28
4.58	0.31	0.90	0.14	0.28
4.6	0.30	0.90	0.14	0.28
4.62	0.28	0.91	0.13	0.29
4.63	0.27	0.91	0.13	0.29
4.65	0.25	0.91	0.13	0.29

4.67	0.23	0.92	0.13	0.30
4.68	0.21	0.92	0.13	0.30
4.7	0.19	0.92	0.13	0.31
4.72	0.18	0.93	0.13	0.31
4.73	0.16	0.93	0.13	0.31
4.75	0.15	0.93	0.13	0.32
4.77	0.14	0.93	0.13	0.32
4.78	0.14	0.93	0.13	0.32
4.8	0.14	0.93	0.12	0.32
4.82	0.15	0.93	0.12	0.32
4.83	0.17	0.93	0.11	0.31
4.85	0.18	0.93	0.11	0.31
4.87	0.20	0.93	0.10	0.30
4.88	0.21	0.92	0.09	0.30
4.9	0.23	0.92	0.09	0.30
4.92	0.24	0.92	0.08	0.30
4.93	0.24	0.92	0.08	0.30
4.95	0.23	0.92	0.08	0.30
4.97	0.22	0.92	0.08	0.30
4.98	0.21	0.92	0.09	0.31
5	0.19	0.93	0.09	0.31
5.02	0.17	0.93	0.10	0.32
5.03	0.14	0.93	0.11	0.32
5.05	0.12	0.93	0.11	0.32
5.07	0.10	0.93	0.12	0.32
5.08	0.08	0.93	0.12	0.32
5.1	0.07	0.94	0.13	0.32
5.12	0.06	0.94	0.13	0.32
5.13	0.06	0.94	0.13	0.31
5.15	0.07	0.94	0.14	0.30
5.17	0.10	0.94	0.14	0.29
5.18	0.14	0.94	0.14	0.28
5.2	0.20	0.94	0.14	0.26
5.22	0.28	0.92	0.14	0.22
5.23	0.37	0.90	0.13	0.18
5.25	0.49	0.86	0.12	0.13
5.27	0.59	0.79	0.10	0.07
5.28	0.69	0.72	0.09	0.02
5.3	0.75	0.66	0.07	-0.02
5.32	0.79	0.60	0.06	-0.05
5.33	0.82	0.57	0.06	-0.07
5.35	0.83	0.55	0.05	-0.07
5.37	0.84	0.54	0.05	-0.07
5.38	0.84	0.54	0.06	-0.06
5.4	0.83	0.55	0.06	-0.05
5.42	0.82	0.56	0.07	-0.04
5.43	0.81	0.58	0.08	-0.03

5.45	0.79	0.60	0.09	-0.01
5.47	0.78	0.62	0.09	-0.00
5.48	0.76	0.64	0.10	0.01
5.5	0.74	0.66	0.11	0.02
5.52	0.72	0.68	0.11	0.03
5.53	0.70	0.70	0.12	0.04
5.55	0.69	0.72	0.12	0.05
5.57	0.67	0.73	0.13	0.06
5.58	0.65	0.75	0.13	0.07
5.6	0.63	0.76	0.14	0.08
5.62	0.61	0.77	0.14	0.09
5.63	0.59	0.79	0.15	0.11
5.65	0.58	0.79	0.16	0.12
5.67	0.56	0.80	0.16	0.14
5.68	0.55	0.80	0.17	0.16
5.7	0.54	0.81	0.18	0.17
5.72	0.53	0.81	0.19	0.19
5.73	0.52	0.80	0.19	0.21
5.75	0.52	0.80	0.20	0.23
5.77	0.52	0.79	0.21	0.25
5.78	0.52	0.78	0.21	0.27
5.8	0.53	0.77	0.21	0.28
5.82	0.53	0.76	0.22	0.29
5.83	0.55	0.75	0.21	0.30
5.85	0.56	0.74	0.21	0.30
5.87	0.57	0.73	0.21	0.30
5.88	0.59	0.73	0.20	0.30
5.9	0.60	0.72	0.20	0.30
5.92	0.60	0.72	0.19	0.29
5.93	0.61	0.71	0.19	0.29
5.95	0.61	0.72	0.19	0.29
5.97	0.60	0.72	0.19	0.30
5.98	0.59	0.72	0.19	0.30
6	0.59	0.73	0.19	0.31
6.02	0.58	0.73	0.19	0.31
6.03	0.57	0.73	0.19	0.32
6.05	0.56	0.73	0.19	0.33
6.07	0.56	0.73	0.19	0.33
6.08	0.56	0.73	0.19	0.34
6.1	0.56	0.73	0.20	0.34
6.12	0.56	0.72	0.20	0.35
6.13	0.56	0.72	0.21	0.36
6.15	0.56	0.71	0.22	0.36
6.17	0.55	0.72	0.24	0.36
6.18	0.53	0.73	0.25	0.35
6.2	0.51	0.74	0.27	0.34
6.22	0.48	0.76	0.30	0.32

6.23	0.45	0.78	0.32	0.29
6.25	0.42	0.80	0.36	0.25
6.27	0.39	0.80	0.40	0.21
6.28	0.37	0.79	0.46	0.17
6.3	0.35	0.77	0.51	0.13
6.32	0.34	0.74	0.57	0.09
6.33	0.32	0.71	0.63	0.06
6.35	0.31	0.67	0.67	0.03
6.37	0.29	0.64	0.71	0.00
6.38	0.28	0.62	0.73	-0.02
6.4	0.28	0.61	0.74	-0.04
6.42	0.32	0.61	0.73	-0.07
6.43	0.40	0.60	0.68	-0.12
6.45	0.53	0.58	0.60	-0.18
6.47	0.67	0.52	0.47	-0.23
6.48	0.78	0.45	0.33	-0.27
6.5	0.85	0.38	0.22	-0.30
6.52	0.88	0.33	0.14	-0.31
6.53	0.90	0.30	0.09	-0.31
6.55	0.90	0.28	0.07	-0.32
6.57	0.90	0.28	0.06	-0.32
6.58	0.90	0.28	0.06	-0.32
6.6	0.90	0.28	0.06	-0.33
6.62	0.90	0.29	0.07	-0.33
6.63	0.89	0.30	0.09	-0.33
6.65	0.88	0.32	0.11	-0.34
6.67	0.86	0.34	0.14	-0.35
6.68	0.84	0.36	0.18	-0.35
6.7	0.81	0.40	0.24	-0.36
6.72	0.76	0.44	0.31	-0.36
6.73	0.70	0.49	0.39	-0.35
6.75	0.62	0.53	0.47	-0.34
6.77	0.53	0.57	0.53	-0.32
6.78	0.46	0.61	0.58	-0.29
6.8	0.40	0.65	0.59	-0.25
6.82	0.38	0.69	0.58	-0.21
6.83	0.38	0.73	0.54	-0.17
6.85	0.41	0.77	0.48	-0.12
6.87	0.44	0.79	0.41	-0.09
6.88	0.48	0.81	0.34	-0.06
6.9	0.52	0.81	0.28	-0.04
6.92	0.55	0.80	0.23	-0.03
6.93	0.58	0.79	0.20	-0.03
6.95	0.60	0.78	0.18	-0.03
6.97	0.61	0.78	0.16	-0.03
6.98	0.62	0.77	0.15	-0.03
7	0.62	0.77	0.15	-0.03

7.02	0.62	0.77	0.15	-0.03
7.03	0.62	0.77	0.15	-0.03
7.05	0.61	0.78	0.16	-0.03
7.07	0.59	0.79	0.16	-0.02
7.08	0.57	0.80	0.18	-0.01
7.1	0.55	0.81	0.19	0.00
7.12	0.52	0.83	0.21	0.02
7.13	0.49	0.84	0.23	0.03
7.15	0.46	0.85	0.25	0.05
7.17	0.43	0.86	0.26	0.06
7.18	0.42	0.86	0.28	0.07
7.2	0.43	0.86	0.28	0.08
7.22	0.45	0.85	0.27	0.07
7.23	0.50	0.83	0.25	0.06
7.25	0.56	0.80	0.22	0.03
7.27	0.62	0.76	0.18	0.01
7.28	0.67	0.73	0.14	-0.01
7.3	0.71	0.69	0.10	-0.03
7.32	0.74	0.67	0.07	-0.05
7.33	0.75	0.66	0.05	-0.05
7.35	0.74	0.67	0.04	-0.05
7.37	0.73	0.68	0.04	-0.05
7.38	0.70	0.71	0.05	-0.04
7.4	0.67	0.74	0.06	-0.03
7.42	0.64	0.77	0.08	-0.01
7.43	0.61	0.79	0.09	-0.01
7.45	0.59	0.80	0.10	-0.01
7.47	0.59	0.80	0.10	-0.02
7.48	0.61	0.79	0.09	-0.04
7.5	0.65	0.75	0.07	-0.07
7.52	0.69	0.71	0.04	-0.09
7.53	0.74	0.67	0.01	-0.12
7.55	0.77	0.62	-0.01	-0.13
7.57	0.79	0.59	-0.02	-0.14
7.58	0.81	0.57	-0.02	-0.14
7.6	0.81	0.57	-0.01	-0.13
7.62	0.81	0.58	0.01	-0.12
7.63	0.79	0.60	0.04	-0.10
7.65	0.76	0.64	0.09	-0.08
7.67	0.71	0.69	0.14	-0.05
7.68	0.65	0.73	0.20	-0.02
7.7	0.57	0.78	0.27	0.01
7.72	0.49	0.81	0.32	0.04
7.73	0.42	0.83	0.37	0.06
7.75	0.38	0.83	0.40	0.08
7.77	0.36	0.83	0.41	0.08
7.78	0.38	0.83	0.41	0.07

7.8	0.43	0.81	0.40	0.05
7.82	0.50	0.78	0.37	0.03
7.83	0.58	0.75	0.33	-0.00
7.85	0.66	0.70	0.28	-0.04
7.87	0.73	0.65	0.23	-0.06
7.88	0.77	0.60	0.19	-0.09
7.9	0.80	0.57	0.16	-0.10
7.92	0.82	0.55	0.14	-0.11
7.93	0.82	0.54	0.13	-0.11
7.95	0.82	0.55	0.14	-0.10
7.97	0.80	0.57	0.16	-0.09
7.98	0.78	0.59	0.19	-0.07
8	0.75	0.62	0.24	-0.04
8.02	0.71	0.65	0.29	-0.00
8.03	0.67	0.66	0.34	0.03
8.05	0.63	0.66	0.39	0.06
8.07	0.62	0.65	0.43	0.09
8.08	0.63	0.62	0.45	0.10
8.1	0.67	0.58	0.45	0.11
8.12	0.73	0.52	0.43	0.10
8.13	0.80	0.45	0.38	0.09
8.15	0.86	0.38	0.33	0.07
8.17	0.90	0.32	0.29	0.05
8.18	0.93	0.27	0.25	0.04
8.2	0.94	0.24	0.24	0.03
8.22	0.95	0.21	0.25	0.02
8.23	0.94	0.17	0.30	0.02
8.25	0.91	0.13	0.40	0.02
8.27	0.83	0.07	0.56	-0.00
8.28	0.67	-0.02	0.74	-0.04
8.3	0.47	-0.11	0.87	-0.08
8.32	0.30	-0.17	0.93	-0.12
8.33	0.19	-0.21	0.95	-0.15
8.35	0.12	-0.24	0.95	-0.17
8.37	0.08	-0.25	0.95	-0.18
8.38	0.06	-0.26	0.95	-0.19
8.4	0.05	-0.26	0.94	-0.20
8.42	0.05	-0.27	0.94	-0.20
8.43	0.05	-0.27	0.94	-0.21
8.45	0.05	-0.27	0.94	-0.21
8.47	0.05	-0.27	0.94	-0.21
8.48	0.05	-0.27	0.94	-0.21
8.5	0.06	-0.27	0.94	-0.21
8.52	0.06	-0.27	0.94	-0.21
8.53	0.06	-0.27	0.94	-0.21
8.55	0.06	-0.27	0.94	-0.21
8.57	0.06	-0.27	0.94	-0.21

8.58	0.06	-0.27	0.94	-0.21
8.6	0.06	-0.27	0.94	-0.22
8.62	0.07	-0.26	0.94	-0.22
8.63	0.09	-0.26	0.94	-0.22
8.65	0.11	-0.25	0.94	-0.21
8.67	0.15	-0.23	0.94	-0.21
8.68	0.20	-0.21	0.94	-0.20
8.7	0.26	-0.18	0.93	-0.19
8.72	0.34	-0.14	0.92	-0.16
8.73	0.43	-0.09	0.89	-0.13
8.75	0.51	-0.03	0.85	-0.10
8.77	0.59	0.03	0.80	-0.06
8.78	0.65	0.08	0.76	-0.02
8.8	0.68	0.12	0.72	0.00
8.82	0.70	0.16	0.70	0.02
8.83	0.70	0.18	0.69	0.04
8.85	0.69	0.20	0.69	0.06
8.87	0.67	0.22	0.70	0.08
8.88	0.65	0.23	0.72	0.11
8.9	0.62	0.24	0.73	0.13
8.92	0.59	0.24	0.75	0.16
8.93	0.56	0.24	0.77	0.18
8.95	0.53	0.23	0.79	0.19
8.97	0.51	0.22	0.81	0.20
8.98	0.49	0.21	0.82	0.20
9	0.49	0.20	0.83	0.19
9.02	0.49	0.20	0.83	0.18
9.03	0.49	0.20	0.83	0.16
9.05	0.50	0.20	0.83	0.13
9.07	0.51	0.21	0.82	0.11
9.08	0.53	0.22	0.81	0.08
9.1	0.56	0.24	0.79	0.05
9.12	0.59	0.26	0.76	0.02
9.13	0.64	0.27	0.72	-0.01
9.15	0.69	0.29	0.66	-0.04
9.17	0.74	0.31	0.59	-0.07
9.18	0.79	0.32	0.51	-0.10
9.2	0.84	0.33	0.42	-0.12
9.22	0.87	0.34	0.34	-0.13
9.23	0.89	0.34	0.28	-0.14
9.25	0.90	0.35	0.23	-0.15
9.27	0.90	0.36	0.19	-0.15
9.28	0.90	0.38	0.17	-0.14
9.3	0.89	0.40	0.16	-0.13
9.32	0.88	0.42	0.17	-0.13
9.33	0.87	0.45	0.18	-0.12
9.35	0.84	0.49	0.20	-0.10

9.37	0.82	0.52	0.21	-0.09
9.38	0.80	0.55	0.22	-0.09
9.4	0.79	0.57	0.22	-0.08
9.42	0.78	0.59	0.21	-0.09
9.43	0.77	0.60	0.20	-0.09
9.45	0.77	0.60	0.18	-0.10
9.47	0.77	0.61	0.16	-0.10
9.48	0.77	0.61	0.14	-0.11
9.5	0.77	0.62	0.13	-0.11
9.52	0.76	0.63	0.12	-0.12
9.53	0.75	0.64	0.12	-0.12
9.55	0.74	0.65	0.12	-0.12
9.57	0.72	0.67	0.13	-0.12
9.58	0.71	0.68	0.14	-0.12
9.6	0.70	0.69	0.15	-0.11
9.62	0.69	0.70	0.17	-0.11
9.63	0.68	0.70	0.19	-0.11
9.65	0.68	0.70	0.20	-0.11
9.67	0.69	0.69	0.22	-0.11
9.68	0.70	0.67	0.22	-0.11
9.7	0.71	0.65	0.23	-0.11
9.72	0.73	0.63	0.23	-0.11
9.73	0.75	0.61	0.23	-0.11
9.75	0.77	0.59	0.23	-0.11
9.77	0.79	0.56	0.23	-0.10
9.78	0.80	0.54	0.23	-0.10
9.8	0.81	0.52	0.23	-0.10
9.82	0.83	0.50	0.23	-0.10
9.83	0.84	0.48	0.22	-0.10
9.85	0.86	0.45	0.22	-0.10
9.87	0.87	0.43	0.21	-0.11
9.88	0.88	0.41	0.19	-0.11
9.9	0.89	0.39	0.18	-0.12
9.92	0.90	0.38	0.17	-0.12
9.93	0.91	0.37	0.16	-0.12
9.95	0.91	0.36	0.15	-0.12
9.97	0.91	0.36	0.15	-0.12
9.98	0.91	0.36	0.15	-0.12
10	0.91	0.37	0.16	-0.11
10.02	0.91	0.37	0.17	-0.10
10.03	0.90	0.38	0.19	-0.09
10.05	0.89	0.39	0.21	-0.08
10.07	0.88	0.40	0.24	-0.07
10.08	0.87	0.41	0.26	-0.06
10.1	0.86	0.41	0.29	-0.04
10.12	0.85	0.42	0.31	-0.03
10.13	0.84	0.42	0.34	-0.02

10.15	0.84	0.41	0.36	-0.01
10.17	0.83	0.41	0.38	0.00
10.18	0.83	0.40	0.40	0.01
10.2	0.82	0.39	0.41	0.02
10.22	0.81	0.39	0.43	0.03
10.23	0.81	0.38	0.45	0.04
10.25	0.80	0.37	0.47	0.05
10.27	0.79	0.37	0.49	0.06
10.28	0.78	0.36	0.51	0.07
10.3	0.76	0.35	0.54	0.07
10.32	0.75	0.34	0.56	0.07
10.33	0.73	0.33	0.59	0.07
10.35	0.72	0.32	0.61	0.06
10.37	0.71	0.31	0.63	0.05
10.38	0.70	0.30	0.65	0.04
10.4	0.69	0.30	0.66	0.03
10.42	0.69	0.29	0.66	0.02
10.43	0.69	0.29	0.67	0.01
10.45	0.69	0.28	0.66	-0.01
10.47	0.70	0.28	0.65	-0.02
10.48	0.71	0.28	0.64	-0.03
10.5	0.72	0.29	0.63	-0.03
10.52	0.73	0.29	0.62	-0.04
10.53	0.73	0.30	0.61	-0.04
10.55	0.74	0.30	0.60	-0.04
10.57	0.74	0.32	0.59	-0.04
10.58	0.73	0.34	0.59	-0.04
10.6	0.73	0.37	0.58	-0.03
10.62	0.71	0.41	0.58	-0.02
10.63	0.68	0.45	0.57	-0.02
10.65	0.65	0.51	0.56	-0.01
10.67	0.61	0.57	0.55	0.00
10.68	0.56	0.63	0.54	0.01
10.7	0.51	0.69	0.52	0.02
10.72	0.46	0.74	0.49	0.03
10.73	0.42	0.78	0.46	0.04
10.75	0.38	0.82	0.43	0.04
10.77	0.35	0.84	0.41	0.05
10.78	0.33	0.86	0.38	0.05
10.8	0.31	0.88	0.35	0.05
10.82	0.30	0.90	0.32	0.05
10.83	0.29	0.91	0.30	0.06
10.85	0.28	0.92	0.27	0.06
10.87	0.28	0.93	0.25	0.06
10.88	0.27	0.93	0.23	0.06
10.9	0.25	0.94	0.21	0.07
10.92	0.24	0.95	0.20	0.07

10.93	0.23	0.95	0.19	0.07
10.95	0.22	0.96	0.18	0.07
10.97	0.21	0.96	0.18	0.07
10.98	0.21	0.96	0.18	0.07
11	0.21	0.96	0.19	0.07
11.02	0.22	0.95	0.20	0.07
11.03	0.23	0.95	0.21	0.07
11.05	0.25	0.94	0.22	0.06
11.07	0.28	0.93	0.24	0.06
11.08	0.32	0.91	0.26	0.06
11.1	0.37	0.88	0.29	0.05
11.12	0.43	0.85	0.32	0.04
11.13	0.49	0.80	0.34	0.03
11.15	0.55	0.75	0.37	0.03
11.17	0.60	0.69	0.40	0.02
11.18	0.64	0.64	0.42	0.02
11.2	0.68	0.59	0.44	0.01
11.22	0.70	0.55	0.45	0.01
11.23	0.72	0.52	0.46	0.01
11.25	0.73	0.50	0.46	0.02
11.27	0.75	0.48	0.46	0.02
11.28	0.76	0.47	0.45	0.03
11.3	0.77	0.46	0.45	0.03
11.32	0.78	0.45	0.44	0.04
11.33	0.79	0.44	0.43	0.04
11.35	0.80	0.44	0.42	0.05
11.37	0.80	0.43	0.41	0.05
11.38	0.81	0.43	0.40	0.05
11.4	0.81	0.42	0.40	0.05
11.42	0.81	0.42	0.40	0.05
11.43	0.81	0.42	0.40	0.05
11.45	0.81	0.42	0.40	0.05
11.47	0.81	0.42	0.40	0.04
11.48	0.81	0.43	0.41	0.04
11.5	0.80	0.43	0.41	0.04
11.52	0.80	0.43	0.42	0.03
11.53	0.80	0.43	0.42	0.03
11.55	0.79	0.44	0.42	0.03
11.57	0.79	0.44	0.42	0.02
11.58	0.79	0.44	0.42	0.02
11.6	0.79	0.45	0.42	0.02
11.62	0.79	0.45	0.41	0.01
11.63	0.79	0.46	0.40	0.01
11.65	0.79	0.47	0.39	0.01
11.67	0.79	0.48	0.38	0.01
11.68	0.79	0.49	0.37	0.01
11.7	0.79	0.50	0.36	0.02

11.72	0.78	0.51	0.35	0.02
11.73	0.78	0.52	0.35	0.03
11.75	0.77	0.53	0.34	0.04
11.77	0.77	0.54	0.34	0.04
11.78	0.76	0.55	0.34	0.05
11.8	0.76	0.56	0.34	0.05
11.82	0.76	0.56	0.34	0.05
11.83	0.76	0.56	0.33	0.05
11.85	0.77	0.55	0.33	0.04
11.87	0.78	0.54	0.32	0.03
11.88	0.79	0.53	0.31	0.02
11.9	0.80	0.52	0.29	0.01
11.92	0.82	0.51	0.28	-0.01
11.93	0.83	0.49	0.27	-0.02
11.95	0.84	0.48	0.26	-0.03
11.97	0.85	0.47	0.25	-0.04
11.98	0.85	0.46	0.24	-0.05
12	0.86	0.46	0.24	-0.06
12.02	0.86	0.45	0.24	-0.06
12.03	0.86	0.45	0.24	-0.07
12.05	0.86	0.45	0.25	-0.07
12.07	0.85	0.45	0.25	-0.07
12.08	0.85	0.45	0.26	-0.07
12.1	0.84	0.45	0.28	-0.07
12.12	0.84	0.46	0.29	-0.07
12.13	0.83	0.46	0.30	-0.07
12.15	0.82	0.47	0.31	-0.07
12.17	0.82	0.47	0.32	-0.06
12.18	0.81	0.48	0.33	-0.06
12.2	0.81	0.48	0.34	-0.06
12.22	0.81	0.48	0.34	-0.06
12.23	0.81	0.48	0.33	-0.05
12.25	0.81	0.48	0.33	-0.05
12.27	0.81	0.49	0.32	-0.05
12.28	0.81	0.49	0.31	-0.05
12.3	0.82	0.49	0.31	-0.05
12.32	0.82	0.49	0.30	-0.05
12.33	0.82	0.49	0.29	-0.05
12.35	0.82	0.50	0.29	-0.05
12.37	0.81	0.50	0.29	-0.05
12.38	0.81	0.51	0.28	-0.05
12.4	0.81	0.52	0.28	-0.04
12.42	0.80	0.53	0.28	-0.04
12.43	0.79	0.54	0.28	-0.03
12.45	0.79	0.55	0.28	-0.03
12.47	0.78	0.56	0.28	-0.02
12.48	0.77	0.57	0.28	-0.02

12.5	0.77	0.58	0.28	-0.01
12.52	0.76	0.59	0.28	-0.01
12.53	0.76	0.59	0.27	-0.01
12.55	0.75	0.60	0.27	-0.00
12.57	0.75	0.61	0.26	-0.00
12.58	0.74	0.62	0.26	-0.00
12.6	0.74	0.62	0.25	0.00
12.62	0.74	0.63	0.25	0.00
12.63	0.74	0.63	0.24	0.00
12.65	0.73	0.64	0.24	0.00
12.67	0.73	0.64	0.23	-0.00
12.68	0.73	0.64	0.23	-0.00
12.7	0.73	0.64	0.22	-0.00
12.72	0.74	0.64	0.22	-0.01
12.73	0.74	0.64	0.21	-0.01
12.75	0.74	0.64	0.21	-0.01
12.77	0.74	0.64	0.21	-0.01
12.78	0.75	0.63	0.20	-0.02
12.8	0.76	0.62	0.19	-0.02
12.82	0.77	0.61	0.18	-0.03
12.83	0.78	0.60	0.17	-0.03
12.85	0.80	0.58	0.15	-0.04
12.87	0.82	0.56	0.13	-0.06
12.88	0.84	0.53	0.11	-0.07
12.9	0.86	0.50	0.08	-0.09
12.92	0.88	0.46	0.05	-0.10
12.93	0.90	0.43	0.02	-0.12
12.95	0.91	0.39	-0.01	-0.14
12.97	0.92	0.36	-0.04	-0.15
12.98	0.93	0.33	-0.06	-0.16
13	0.93	0.31	-0.07	-0.17
13.02	0.94	0.29	-0.09	-0.18
13.03	0.94	0.27	-0.10	-0.18
13.05	0.94	0.26	-0.11	-0.19
13.07	0.94	0.25	-0.11	-0.19
13.08	0.94	0.24	-0.12	-0.19
13.1	0.94	0.24	-0.12	-0.19
13.12	0.95	0.24	-0.12	-0.18
13.13	0.94	0.25	-0.11	-0.18
13.15	0.94	0.26	-0.11	-0.17
13.17	0.94	0.28	-0.10	-0.17
13.18	0.94	0.30	-0.09	-0.16
13.2	0.93	0.33	-0.08	-0.15
13.22	0.92	0.37	-0.06	-0.13
13.23	0.90	0.42	-0.05	-0.12
13.25	0.87	0.49	-0.02	-0.09
13.27	0.83	0.56	0.00	-0.07

13.28	0.77	0.63	0.03	-0.04
13.3	0.70	0.71	0.05	-0.01
13.32	0.62	0.78	0.08	0.02
13.33	0.54	0.84	0.09	0.04
13.35	0.45	0.88	0.11	0.06
13.37	0.38	0.92	0.11	0.08
13.38	0.31	0.94	0.12	0.09
13.4	0.25	0.96	0.12	0.10
13.42	0.20	0.97	0.12	0.11
13.43	0.16	0.97	0.11	0.11
13.45	0.13	0.98	0.11	0.12
13.47	0.10	0.98	0.10	0.12
13.48	0.08	0.98	0.10	0.12
13.5	0.06	0.99	0.10	0.12
13.52	0.05	0.99	0.09	0.12
13.53	0.04	0.99	0.09	0.12
13.55	0.04	0.99	0.08	0.12
13.57	0.03	0.99	0.08	0.11
13.58	0.03	0.99	0.08	0.11
13.6	0.03	0.99	0.08	0.11
13.62	0.04	0.99	0.08	0.11
13.63	0.05	0.99	0.08	0.11
13.65	0.06	0.99	0.08	0.10
13.67	0.07	0.99	0.08	0.10
13.68	0.08	0.99	0.09	0.10
13.7	0.10	0.99	0.09	0.10
13.72	0.13	0.98	0.09	0.09
13.73	0.15	0.98	0.10	0.09
13.75	0.18	0.97	0.10	0.09
13.77	0.22	0.97	0.11	0.08
13.78	0.26	0.96	0.11	0.08
13.8	0.30	0.94	0.12	0.08
13.82	0.35	0.93	0.12	0.07
13.83	0.40	0.91	0.13	0.07
13.85	0.45	0.88	0.13	0.07
13.87	0.50	0.85	0.14	0.07
13.88	0.54	0.82	0.14	0.07
13.9	0.59	0.79	0.14	0.06
13.92	0.63	0.76	0.14	0.06
13.93	0.67	0.73	0.14	0.06
13.95	0.70	0.69	0.14	0.06
13.97	0.73	0.66	0.14	0.06
13.98	0.76	0.63	0.13	0.06
14	0.78	0.61	0.13	0.06
14.02	0.80	0.58	0.13	0.06
14.03	0.81	0.56	0.13	0.07
14.05	0.83	0.54	0.13	0.07

14.07	0.84	0.52	0.13	0.08
14.08	0.85	0.51	0.13	0.08
14.1	0.86	0.49	0.13	0.09
14.12	0.86	0.48	0.13	0.09
14.13	0.87	0.46	0.13	0.10
14.15	0.88	0.45	0.13	0.11
14.17	0.88	0.44	0.13	0.11
14.18	0.89	0.43	0.13	0.11
14.2	0.89	0.42	0.13	0.12
14.22	0.89	0.41	0.13	0.12
14.23	0.90	0.41	0.14	0.12
14.25	0.90	0.40	0.14	0.12
14.27	0.90	0.40	0.14	0.13
14.28	0.90	0.40	0.14	0.13
14.3	0.90	0.40	0.14	0.13
14.32	0.89	0.41	0.14	0.13
14.33	0.89	0.41	0.14	0.12
14.35	0.89	0.42	0.14	0.12
14.37	0.89	0.42	0.14	0.12
14.38	0.88	0.43	0.15	0.11
14.4	0.88	0.44	0.15	0.11
14.42	0.88	0.45	0.15	0.10
14.43	0.87	0.46	0.15	0.10
14.45	0.87	0.47	0.15	0.09
14.47	0.86	0.48	0.16	0.08
14.48	0.86	0.48	0.16	0.08
14.5	0.85	0.49	0.16	0.08
14.52	0.85	0.50	0.17	0.07
14.53	0.84	0.51	0.17	0.07
14.55	0.83	0.52	0.17	0.07
14.57	0.83	0.53	0.18	0.07
14.58	0.82	0.54	0.18	0.08
14.6	0.82	0.54	0.18	0.08
14.62	0.81	0.55	0.18	0.09
14.63	0.80	0.56	0.18	0.10
14.65	0.79	0.57	0.19	0.11
14.67	0.78	0.58	0.19	0.13
14.68	0.76	0.60	0.19	0.14
14.7	0.75	0.61	0.19	0.16
14.72	0.73	0.63	0.19	0.18
14.73	0.71	0.65	0.19	0.21
14.75	0.69	0.67	0.18	0.23
14.77	0.66	0.68	0.18	0.25
14.78	0.63	0.70	0.17	0.28
14.8	0.60	0.72	0.16	0.31
14.82	0.57	0.73	0.16	0.33
14.83	0.54	0.74	0.15	0.36

14.85	0.52	0.75	0.14	0.38
14.87	0.49	0.76	0.13	0.41
14.88	0.46	0.76	0.13	0.43
14.9	0.44	0.77	0.12	0.45
14.92	0.42	0.77	0.11	0.47
14.93	0.40	0.77	0.11	0.49
14.95	0.38	0.76	0.10	0.51
14.97	0.37	0.76	0.09	0.53
14.98	0.36	0.75	0.09	0.54
15	0.35	0.75	0.08	0.56
15.02	0.34	0.74	0.08	0.57
15.03	0.34	0.74	0.07	0.58
15.05	0.34	0.73	0.07	0.59
15.07	0.34	0.73	0.07	0.59
15.08	0.34	0.72	0.06	0.60
15.1	0.35	0.72	0.06	0.60
15.12	0.36	0.71	0.06	0.60
15.13	0.36	0.71	0.06	0.60
15.15	0.37	0.71	0.06	0.60
15.17	0.37	0.71	0.05	0.60
15.18	0.38	0.71	0.05	0.59
15.2	0.38	0.72	0.05	0.57
15.22	0.38	0.74	0.05	0.56
15.23	0.37	0.75	0.05	0.54
15.25	0.37	0.78	0.04	0.51
15.27	0.35	0.80	0.04	0.48
15.28	0.34	0.83	0.04	0.45
15.3	0.32	0.86	0.04	0.41
15.32	0.29	0.88	0.04	0.36
15.33	0.26	0.91	0.03	0.32
15.35	0.24	0.93	0.03	0.27
15.37	0.21	0.95	0.03	0.23
15.38	0.18	0.96	0.02	0.19
15.4	0.15	0.98	0.02	0.15
15.42	0.13	0.98	0.02	0.12
15.43	0.11	0.99	0.01	0.09
15.45	0.09	0.99	0.01	0.07
15.47	0.07	1.00	0.01	0.05
15.48	0.06	1.00	0.01	0.03
15.5	0.05	1.00	0.01	0.02
15.52	0.04	1.00	0.00	0.01
15.53	0.03	1.00	0.00	0.00
15.55	0.02	1.00	0.00	-0.00
15.57	0.02	1.00	0.00	-0.01
15.58	0.01	1.00	0.00	-0.01
15.6	0.01	1.00	0.00	-0.02
15.62	0.01	1.00	0.00	-0.02

15.63	0.01	1.00	0.00	-0.02
15.65	0.01	1.00	0.01	-0.02
15.67	0.02	1.00	0.01	-0.02
15.68	0.02	1.00	0.01	-0.02
15.7	0.03	1.00	0.01	-0.02
15.72	0.04	1.00	0.01	-0.02
15.73	0.06	1.00	0.02	-0.02
15.75	0.07	1.00	0.02	-0.01
15.77	0.10	0.99	0.03	-0.01
15.78	0.12	0.99	0.03	-0.01
15.8	0.15	0.99	0.04	-0.01
15.82	0.19	0.98	0.05	-0.00
15.83	0.23	0.97	0.06	0.00
15.85	0.27	0.96	0.07	0.00
15.87	0.32	0.94	0.08	0.01
15.88	0.37	0.92	0.09	0.02
15.9	0.43	0.90	0.10	0.02
15.92	0.48	0.87	0.11	0.03
15.93	0.52	0.84	0.12	0.04
15.95	0.57	0.81	0.13	0.04
15.97	0.61	0.78	0.14	0.05
15.98	0.64	0.75	0.15	0.05
16	0.67	0.72	0.15	0.06
16.02	0.70	0.70	0.16	0.06
16.03	0.72	0.67	0.16	0.06
16.05	0.74	0.65	0.16	0.06
16.07	0.75	0.63	0.16	0.06
16.08	0.77	0.62	0.16	0.06
16.1	0.78	0.60	0.16	0.05
16.12	0.79	0.59	0.15	0.05
16.13	0.80	0.58	0.15	0.05
16.15	0.81	0.57	0.14	0.05
16.17	0.81	0.56	0.14	0.05
16.18	0.82	0.56	0.14	0.05
16.2	0.82	0.56	0.13	0.06
16.22	0.82	0.56	0.13	0.06
16.23	0.82	0.56	0.13	0.06
16.25	0.82	0.56	0.13	0.06
16.27	0.82	0.56	0.12	0.06
16.28	0.81	0.56	0.12	0.06
16.3	0.81	0.57	0.12	0.06
16.32	0.81	0.57	0.11	0.06
16.33	0.81	0.57	0.11	0.06
16.35	0.81	0.58	0.11	0.06
16.37	0.81	0.58	0.11	0.07
16.38	0.80	0.58	0.11	0.07
16.4	0.80	0.58	0.11	0.07

16.42	0.80	0.58	0.11	0.07
16.43	0.81	0.58	0.11	0.07
16.45	0.81	0.58	0.11	0.07
16.47	0.81	0.57	0.11	0.07
16.48	0.81	0.57	0.11	0.06
16.5	0.81	0.57	0.10	0.06
16.52	0.82	0.56	0.10	0.06
16.53	0.82	0.56	0.10	0.06
16.55	0.82	0.56	0.10	0.06
16.57	0.83	0.55	0.09	0.05
16.58	0.83	0.55	0.09	0.05
16.6	0.83	0.54	0.09	0.05
16.62	0.83	0.54	0.08	0.05
16.63	0.84	0.54	0.08	0.05
16.65	0.84	0.54	0.08	0.05
16.67	0.84	0.53	0.08	0.05
16.68	0.84	0.53	0.08	0.05
16.7	0.84	0.53	0.08	0.05
16.72	0.85	0.52	0.07	0.06
16.73	0.85	0.52	0.07	0.06
16.75	0.85	0.52	0.07	0.06
16.77	0.85	0.52	0.06	0.06
16.78	0.85	0.52	0.06	0.06
16.8	0.85	0.52	0.06	0.06
16.82	0.85	0.52	0.06	0.06
16.83	0.85	0.52	0.07	0.06
16.85	0.84	0.53	0.07	0.06
16.87	0.84	0.53	0.07	0.06
16.88	0.84	0.53	0.07	0.06
16.9	0.84	0.53	0.07	0.06
16.92	0.84	0.54	0.06	0.06
16.93	0.84	0.54	0.06	0.05
16.95	0.84	0.54	0.05	0.05
16.97	0.84	0.53	0.05	0.05
16.98	0.84	0.53	0.04	0.04
17	0.85	0.53	0.04	0.04
17.02	0.85	0.52	0.03	0.04
17.03	0.85	0.52	0.03	0.03
17.05	0.86	0.52	0.02	0.03
17.07	0.86	0.51	0.02	0.02
17.08	0.86	0.51	0.01	0.02
17.1	0.86	0.50	0.01	0.02
17.12	0.87	0.50	-0.00	0.01
17.13	0.87	0.50	-0.01	0.01
17.15	0.87	0.50	-0.01	0.01
17.17	0.87	0.50	-0.02	0.00
17.18	0.87	0.50	-0.02	0.00

17.2	0.86	0.50	-0.02	-0.00
17.22	0.86	0.51	-0.03	-0.00
17.23	0.86	0.51	-0.03	-0.00
17.25	0.86	0.52	-0.03	0.00
17.27	0.85	0.52	-0.03	0.00
17.28	0.85	0.53	-0.03	0.01
17.3	0.85	0.53	-0.03	0.02
17.32	0.84	0.54	-0.03	0.03
17.33	0.84	0.54	-0.03	0.04
17.35	0.84	0.54	-0.02	0.05
17.37	0.83	0.55	-0.02	0.07
17.38	0.83	0.55	-0.02	0.09
17.4	0.83	0.55	-0.02	0.11
17.42	0.83	0.55	-0.01	0.13
17.43	0.82	0.55	-0.01	0.16
17.45	0.82	0.54	-0.00	0.18
17.47	0.82	0.54	0.00	0.20
17.48	0.81	0.54	0.01	0.22
17.5	0.81	0.54	0.02	0.24
17.52	0.80	0.54	0.03	0.26
17.53	0.80	0.54	0.05	0.27
17.55	0.79	0.54	0.07	0.28
17.57	0.78	0.54	0.09	0.29
17.58	0.77	0.54	0.12	0.30
17.6	0.76	0.55	0.15	0.31
17.62	0.75	0.55	0.18	0.31
17.63	0.74	0.56	0.22	0.31
17.65	0.72	0.56	0.26	0.32
17.67	0.70	0.57	0.30	0.31
17.68	0.68	0.57	0.35	0.31
17.7	0.66	0.57	0.39	0.30
17.72	0.64	0.57	0.44	0.29
17.73	0.61	0.56	0.48	0.28
17.75	0.59	0.56	0.52	0.27
17.77	0.56	0.55	0.57	0.26
17.78	0.54	0.54	0.61	0.24
17.8	0.51	0.53	0.64	0.22
17.82	0.49	0.51	0.67	0.21
17.83	0.47	0.50	0.70	0.19
17.85	0.46	0.49	0.73	0.17
17.87	0.45	0.47	0.75	0.14
17.88	0.44	0.45	0.76	0.12
17.9	0.44	0.44	0.78	0.09
17.92	0.45	0.42	0.78	0.07
17.93	0.47	0.41	0.78	0.04
17.95	0.49	0.39	0.78	0.01
17.97	0.52	0.37	0.77	-0.03

17.98	0.56	0.35	0.75	-0.06
18	0.61	0.33	0.72	-0.10
18.02	0.65	0.31	0.68	-0.13
18.03	0.70	0.29	0.63	-0.17
18.05	0.75	0.27	0.57	-0.20
18.07	0.79	0.25	0.51	-0.22
18.08	0.83	0.23	0.45	-0.24
18.1	0.86	0.21	0.38	-0.26
18.12	0.89	0.19	0.32	-0.27
18.13	0.91	0.18	0.26	-0.28
18.15	0.92	0.17	0.20	-0.28
18.17	0.93	0.16	0.15	-0.29
18.18	0.94	0.15	0.10	-0.28
18.2	0.95	0.15	0.06	-0.28
18.22	0.95	0.14	0.03	-0.28
18.23	0.95	0.14	-0.01	-0.27
18.25	0.95	0.14	-0.03	-0.27
18.27	0.95	0.15	-0.06	-0.26
18.28	0.95	0.15	-0.07	-0.25
18.3	0.95	0.16	-0.09	-0.25
18.32	0.95	0.16	-0.10	-0.24
18.33	0.95	0.17	-0.11	-0.23
18.35	0.95	0.18	-0.11	-0.22
18.37	0.95	0.19	-0.12	-0.21
18.38	0.95	0.20	-0.12	-0.20
18.4	0.95	0.22	-0.11	-0.19
18.42	0.95	0.24	-0.11	-0.17
18.43	0.95	0.26	-0.10	-0.16
18.45	0.95	0.28	-0.09	-0.14
18.47	0.94	0.30	-0.07	-0.13
18.48	0.94	0.33	-0.06	-0.11
18.5	0.93	0.35	-0.05	-0.10
18.52	0.92	0.38	-0.03	-0.08
18.53	0.91	0.40	-0.01	-0.06
18.55	0.91	0.42	-0.00	-0.05
18.57	0.90	0.44	0.01	-0.04
18.58	0.89	0.46	0.02	-0.02
18.6	0.88	0.47	0.03	-0.01
18.62	0.87	0.48	0.04	-0.01
18.63	0.87	0.49	0.05	-0.00
18.65	0.86	0.50	0.05	-0.00
18.67	0.86	0.51	0.06	-0.00
18.68	0.86	0.51	0.06	-0.01
18.7	0.85	0.52	0.06	-0.01
18.72	0.85	0.52	0.06	-0.01
18.73	0.85	0.52	0.06	-0.01
18.75	0.85	0.52	0.06	-0.01

18.77	0.85	0.52	0.06	-0.01
18.78	0.85	0.52	0.06	-0.00
18.8	0.85	0.52	0.06	0.00
18.82	0.86	0.51	0.06	0.01
18.83	0.86	0.51	0.07	0.02
18.85	0.86	0.51	0.07	0.03
18.87	0.86	0.50	0.08	0.04
18.88	0.86	0.50	0.08	0.04
18.9	0.87	0.49	0.09	0.05
18.92	0.87	0.48	0.09	0.06
18.93	0.87	0.48	0.10	0.07
18.95	0.87	0.47	0.10	0.07
18.97	0.87	0.47	0.11	0.08
18.98	0.88	0.46	0.12	0.08
19	0.88	0.46	0.12	0.08
19.02	0.88	0.46	0.13	0.08
19.03	0.88	0.46	0.13	0.08
19.05	0.87	0.46	0.14	0.07
19.07	0.87	0.46	0.14	0.07
19.08	0.87	0.46	0.14	0.07
19.1	0.87	0.47	0.14	0.06
19.12	0.87	0.47	0.14	0.06
19.13	0.87	0.47	0.14	0.06
19.15	0.87	0.48	0.14	0.06
19.17	0.86	0.48	0.14	0.05
19.18	0.86	0.48	0.14	0.05
19.2	0.86	0.48	0.14	0.04
19.22	0.86	0.49	0.14	0.04
19.23	0.86	0.49	0.15	0.03
19.25	0.86	0.49	0.15	0.03
19.27	0.86	0.49	0.15	0.03
19.28	0.86	0.49	0.15	0.03
19.3	0.85	0.50	0.14	0.03
19.32	0.85	0.50	0.14	0.03
19.33	0.85	0.51	0.14	0.04
19.35	0.85	0.51	0.14	0.04
19.37	0.84	0.52	0.14	0.05
19.38	0.84	0.52	0.14	0.06
19.4	0.83	0.53	0.14	0.07
19.42	0.82	0.54	0.15	0.08
19.43	0.81	0.56	0.15	0.09
19.45	0.80	0.57	0.16	0.10
19.47	0.79	0.58	0.17	0.12
19.48	0.77	0.59	0.19	0.13
19.5	0.76	0.60	0.21	0.14
19.52	0.74	0.61	0.24	0.14
19.53	0.72	0.62	0.27	0.15

19.55	0.70	0.62	0.31	0.15
19.57	0.68	0.63	0.35	0.14
19.58	0.67	0.62	0.38	0.14
19.6	0.65	0.62	0.42	0.13
19.62	0.64	0.61	0.45	0.12
19.63	0.63	0.60	0.48	0.10
19.65	0.62	0.60	0.50	0.09
19.67	0.62	0.59	0.51	0.08
19.68	0.63	0.57	0.52	0.06
19.7	0.65	0.56	0.51	0.04
19.72	0.68	0.55	0.49	0.03
19.73	0.71	0.53	0.46	0.01
19.75	0.75	0.51	0.42	-0.01
19.77	0.79	0.49	0.37	-0.02
19.78	0.82	0.47	0.33	-0.03
19.8	0.84	0.46	0.28	-0.04
19.82	0.86	0.45	0.24	-0.04
19.83	0.87	0.44	0.21	-0.04
19.85	0.88	0.45	0.18	-0.04
19.87	0.88	0.45	0.17	-0.03
19.88	0.88	0.45	0.16	-0.03
19.9	0.87	0.46	0.15	-0.02
19.92	0.87	0.46	0.15	-0.02
19.93	0.87	0.47	0.14	-0.01
19.95	0.87	0.47	0.14	-0.01
19.97	0.87	0.47	0.13	-0.01
19.98	0.87	0.47	0.13	-0.00
20	0.87	0.47	0.13	0.00
20.02	0.88	0.47	0.12	0.01
20.03	0.88	0.46	0.12	0.01
20.05	0.88	0.46	0.12	0.01
20.07	0.88	0.46	0.12	0.01
20.08	0.88	0.45	0.12	0.02
20.1	0.89	0.45	0.11	0.02
20.12	0.89	0.45	0.11	0.02
20.13	0.89	0.44	0.11	0.02
20.15	0.89	0.44	0.10	0.02
20.17	0.90	0.43	0.09	0.01
20.18	0.91	0.42	0.07	0.00
20.2	0.92	0.40	0.06	-0.01
20.22	0.93	0.37	0.04	-0.03
20.23	0.94	0.34	0.02	-0.06
20.25	0.95	0.32	0.00	-0.08
20.27	0.95	0.29	-0.01	-0.09
20.28	0.96	0.27	-0.02	-0.11
20.3	0.96	0.26	-0.02	-0.11
20.32	0.96	0.25	-0.02	-0.12

20.33	0.96	0.26	-0.02	-0.11
20.35	0.96	0.27	-0.01	-0.11
20.37	0.95	0.29	0.00	-0.09
20.38	0.95	0.31	0.02	-0.07
20.4	0.94	0.34	0.04	-0.04
20.42	0.93	0.37	0.07	-0.01
20.43	0.91	0.40	0.11	0.02
20.45	0.90	0.42	0.15	0.05
20.47	0.88	0.43	0.20	0.07
20.48	0.86	0.44	0.24	0.09
20.5	0.84	0.44	0.29	0.11
20.52	0.83	0.44	0.33	0.11
20.53	0.82	0.43	0.37	0.11
20.55	0.81	0.42	0.39	0.10
20.57	0.82	0.41	0.40	0.09
20.58	0.83	0.40	0.39	0.07
20.6	0.85	0.39	0.35	0.05
20.62	0.87	0.38	0.30	0.03
20.63	0.90	0.37	0.24	0.01
20.65	0.91	0.37	0.17	-0.00
20.67	0.93	0.36	0.12	-0.01
20.68	0.93	0.35	0.08	-0.02
20.7	0.94	0.34	0.06	-0.02
20.72	0.94	0.33	0.07	-0.03
20.73	0.94	0.32	0.09	-0.04
20.75	0.94	0.31	0.12	-0.05
20.77	0.94	0.30	0.15	-0.06
20.78	0.94	0.29	0.19	-0.06
20.8	0.93	0.28	0.24	-0.07
20.82	0.92	0.26	0.28	-0.07
20.83	0.91	0.25	0.33	-0.07
20.85	0.89	0.24	0.38	-0.07
20.87	0.88	0.23	0.42	-0.06
20.88	0.87	0.23	0.44	-0.05
20.9	0.86	0.23	0.45	-0.04
20.92	0.87	0.23	0.43	-0.03
20.93	0.89	0.25	0.39	-0.01
20.95	0.91	0.26	0.33	0.00
20.97	0.92	0.27	0.28	0.01
20.98	0.93	0.27	0.25	0.02
21	0.93	0.27	0.23	0.03
21.02	0.94	0.27	0.22	0.03
21.03	0.94	0.27	0.22	0.03
21.05	0.94	0.28	0.21	0.02
21.07	0.94	0.28	0.21	0.02
21.08	0.93	0.29	0.21	0.01
21.1	0.93	0.30	0.20	0.01

21.12	0.93	0.31	0.20	-0.00
21.13	0.93	0.32	0.19	-0.01
21.15	0.93	0.32	0.18	-0.02
21.17	0.93	0.33	0.16	-0.03
21.18	0.93	0.33	0.15	-0.03
21.2	0.93	0.34	0.12	-0.04
21.22	0.94	0.34	0.10	-0.04
21.23	0.94	0.33	0.08	-0.04
21.25	0.94	0.33	0.05	-0.04
21.27	0.95	0.32	0.04	-0.03
21.28	0.95	0.30	0.02	-0.03
21.3	0.96	0.28	0.01	-0.02
21.32	0.96	0.26	0.00	-0.01
21.33	0.97	0.25	-0.00	0.01
21.35	0.97	0.24	-0.01	0.02
21.37	0.97	0.23	-0.01	0.02
21.38	0.97	0.23	-0.02	0.03
21.4	0.97	0.23	-0.02	0.03
21.42	0.97	0.23	-0.02	0.02
21.43	0.97	0.23	-0.02	0.02
21.45	0.97	0.23	-0.02	0.01
21.47	0.97	0.23	-0.02	0.00
21.48	0.97	0.23	-0.01	-0.01
21.5	0.97	0.23	-0.01	-0.01
21.52	0.97	0.23	-0.01	-0.01
21.53	0.97	0.22	-0.01	-0.01
21.55	0.97	0.22	-0.01	-0.01
21.57	0.97	0.22	-0.02	-0.01
21.58	0.97	0.22	-0.02	-0.00
21.6	0.97	0.22	-0.03	0.01
21.62	0.97	0.22	-0.03	0.02
21.63	0.97	0.22	-0.04	0.04
21.65	0.97	0.22	-0.04	0.05
21.67	0.97	0.22	-0.03	0.07
21.68	0.97	0.21	-0.03	0.09
21.7	0.97	0.21	-0.03	0.10
21.72	0.97	0.21	-0.03	0.10
21.73	0.97	0.21	-0.03	0.09
21.75	0.97	0.21	-0.03	0.08
21.77	0.97	0.21	-0.03	0.07
21.78	0.97	0.22	-0.04	0.06
21.8	0.97	0.22	-0.04	0.06
21.82	0.97	0.23	-0.04	0.05
21.83	0.97	0.23	-0.04	0.05
21.85	0.97	0.24	-0.04	0.06
21.87	0.97	0.24	-0.05	0.06
21.88	0.97	0.24	-0.05	0.07

21.9	0.96	0.25	-0.06	0.09
21.92	0.96	0.25	-0.07	0.10
21.93	0.96	0.25	-0.09	0.11
21.95	0.96	0.24	-0.10	0.11
21.97	0.96	0.24	-0.11	0.12
21.98	0.96	0.23	-0.11	0.11
22	0.96	0.22	-0.11	0.11
22.02	0.96	0.22	-0.10	0.11
22.03	0.97	0.21	-0.09	0.11
22.05	0.97	0.21	-0.08	0.10
22.07	0.97	0.20	-0.07	0.10
22.08	0.97	0.20	-0.05	0.10
22.1	0.97	0.20	-0.04	0.09
22.12	0.98	0.20	-0.03	0.08
22.13	0.98	0.20	-0.02	0.08
22.15	0.98	0.19	-0.02	0.06
22.17	0.98	0.18	-0.02	0.05
22.18	0.98	0.17	-0.03	0.04
22.2	0.99	0.16	-0.06	0.03
22.22	0.99	0.13	-0.10	0.02
22.23	0.98	0.11	-0.16	0.00
22.25	0.97	0.08	-0.23	-0.02
22.27	0.95	0.06	-0.31	-0.05
22.28	0.92	0.04	-0.39	-0.07
22.3	0.88	0.03	-0.47	-0.09
22.32	0.84	0.03	-0.53	-0.10
22.33	0.80	0.04	-0.59	-0.10
22.35	0.77	0.06	-0.62	-0.10
22.37	0.74	0.09	-0.65	-0.11
22.38	0.72	0.12	-0.67	-0.11
22.4	0.71	0.16	-0.68	-0.11
22.42	0.70	0.20	-0.68	-0.11
22.43	0.69	0.25	-0.67	-0.10
22.45	0.68	0.30	-0.66	-0.10
22.47	0.68	0.35	-0.63	-0.09
22.48	0.68	0.41	-0.60	-0.07
22.5	0.68	0.46	-0.56	-0.05
22.52	0.69	0.51	-0.52	-0.03
22.53	0.69	0.56	-0.46	-0.00
22.55	0.68	0.61	-0.40	0.02
22.57	0.68	0.65	-0.33	0.04
22.58	0.67	0.69	-0.25	0.06
22.6	0.67	0.72	-0.17	0.08
22.62	0.66	0.74	-0.08	0.09
22.63	0.66	0.75	-0.00	0.10
22.65	0.67	0.73	0.07	0.10
22.67	0.69	0.71	0.13	0.10

22.68	0.73	0.66	0.16	0.09
22.7	0.78	0.60	0.17	0.07
22.72	0.84	0.51	0.14	0.05
22.73	0.90	0.42	0.09	0.03
22.75	0.95	0.32	0.02	0.01
22.77	0.97	0.24	-0.05	-0.01
22.78	0.98	0.18	-0.11	-0.01
22.8	0.98	0.13	-0.15	-0.00
22.82	0.98	0.09	-0.18	0.01
22.83	0.98	0.07	-0.20	0.03
22.85	0.98	0.06	-0.21	0.06
22.87	0.97	0.05	-0.21	0.08
22.88	0.97	0.05	-0.21	0.09
22.9	0.97	0.05	-0.20	0.11
22.92	0.97	0.05	-0.20	0.12
22.93	0.97	0.06	-0.20	0.12
22.95	0.97	0.07	-0.21	0.11
22.97	0.97	0.08	-0.22	0.09
22.98	0.97	0.08	-0.23	0.07
23	0.97	0.08	-0.24	0.04
23.02	0.96	0.07	-0.26	0.01
23.03	0.96	0.06	-0.26	-0.02
23.05	0.96	0.05	-0.27	-0.04
23.07	0.96	0.05	-0.27	-0.05
23.08	0.96	0.04	-0.28	-0.05
23.1	0.96	0.04	-0.28	-0.05
23.12	0.96	0.04	-0.28	-0.05
23.13	0.96	0.04	-0.28	-0.05
23.15	0.96	0.05	-0.28	-0.05
23.17	0.96	0.05	-0.28	-0.05
23.18	0.96	0.04	-0.28	-0.04
23.2	0.96	0.04	-0.28	-0.04
23.22	0.96	0.04	-0.29	-0.03
23.23	0.95	0.04	-0.29	-0.02
23.25	0.95	0.03	-0.30	-0.02
23.27	0.95	0.04	-0.32	-0.01
23.28	0.94	0.04	-0.33	-0.01
23.3	0.94	0.05	-0.34	-0.00
23.32	0.94	0.06	-0.34	0.00
23.33	0.94	0.07	-0.34	0.00
23.35	0.94	0.08	-0.34	0.01
23.37	0.94	0.09	-0.34	0.01
23.38	0.94	0.08	-0.34	0.02
23.4	0.94	0.08	-0.33	0.02
23.42	0.94	0.07	-0.34	0.02
23.43	0.94	0.05	-0.34	0.02
23.45	0.94	0.04	-0.34	0.02

23.47	0.94	0.03	-0.34	0.03
23.48	0.94	0.02	-0.35	0.04
23.5	0.94	0.02	-0.35	0.05
23.52	0.93	0.01	-0.35	0.06
23.53	0.93	0.00	-0.35	0.07
23.55	0.93	-0.01	-0.36	0.08
23.57	0.93	-0.02	-0.36	0.09
23.58	0.92	-0.04	-0.38	0.09
23.6	0.92	-0.05	-0.39	0.08
23.62	0.91	-0.07	-0.41	0.07
23.63	0.90	-0.08	-0.42	0.06
23.65	0.90	-0.08	-0.43	0.05
23.67	0.89	-0.08	-0.44	0.04
23.68	0.89	-0.08	-0.44	0.03
23.7	0.89	-0.08	-0.44	0.04
23.72	0.90	-0.08	-0.43	0.05
23.73	0.90	-0.09	-0.43	0.06
23.75	0.90	-0.09	-0.42	0.08
23.77	0.90	-0.11	-0.42	0.09
23.78	0.89	-0.12	-0.42	0.10
23.8	0.89	-0.13	-0.43	0.10
23.82	0.88	-0.14	-0.44	0.09
23.83	0.88	-0.15	-0.44	0.08
23.85	0.88	-0.16	-0.45	0.05
23.87	0.88	-0.17	-0.45	0.02
23.88	0.88	-0.18	-0.44	-0.01
23.9	0.89	-0.19	-0.42	-0.04
23.92	0.89	-0.21	-0.39	-0.07
23.93	0.90	-0.23	-0.35	-0.10
23.95	0.91	-0.25	-0.32	-0.12
23.97	0.91	-0.27	-0.29	-0.13
23.98	0.90	-0.29	-0.29	-0.14
24	0.89	-0.30	-0.31	-0.14
24.02	0.87	-0.30	-0.36	-0.14
24.03	0.84	-0.29	-0.43	-0.13
24.05	0.80	-0.29	-0.51	-0.13
24.07	0.75	-0.27	-0.60	-0.12
24.08	0.69	-0.26	-0.66	-0.12
24.1	0.64	-0.26	-0.72	-0.12
24.12	0.60	-0.25	-0.75	-0.12
24.13	0.57	-0.25	-0.77	-0.12
24.15	0.57	-0.25	-0.78	-0.11
24.17	0.57	-0.25	-0.77	-0.10
24.18	0.59	-0.25	-0.76	-0.09
24.2	0.62	-0.25	-0.74	-0.06
24.22	0.65	-0.25	-0.72	-0.04
24.23	0.68	-0.26	-0.69	-0.02

24.25	0.70	-0.26	-0.66	0.00
24.27	0.72	-0.26	-0.64	0.01
24.28	0.73	-0.25	-0.63	0.02
24.3	0.73	-0.25	-0.63	0.01
24.32	0.73	-0.24	-0.64	0.00
24.33	0.71	-0.24	-0.66	-0.01
24.35	0.70	-0.23	-0.68	-0.02
24.37	0.68	-0.23	-0.69	-0.03
24.38	0.66	-0.24	-0.71	-0.04
24.4	0.65	-0.25	-0.72	-0.04
24.42	0.63	-0.26	-0.73	-0.04
24.43	0.62	-0.27	-0.74	-0.04
24.45	0.61	-0.28	-0.74	-0.03
24.47	0.61	-0.29	-0.74	-0.03
24.48	0.61	-0.29	-0.73	-0.02
24.5	0.62	-0.29	-0.73	-0.02
24.52	0.64	-0.28	-0.72	-0.02
24.53	0.66	-0.26	-0.71	-0.01
24.55	0.68	-0.25	-0.69	-0.01
24.57	0.69	-0.23	-0.69	-0.00
24.58	0.70	-0.21	-0.68	0.00
24.6	0.71	-0.20	-0.68	0.01
24.62	0.71	-0.19	-0.68	0.01
24.63	0.70	-0.19	-0.69	0.03
24.65	0.69	-0.20	-0.70	0.05
24.67	0.67	-0.21	-0.71	0.07
24.68	0.65	-0.22	-0.72	0.10
24.7	0.63	-0.24	-0.72	0.13
24.72	0.62	-0.25	-0.73	0.17
24.73	0.60	-0.26	-0.73	0.20
24.75	0.59	-0.27	-0.73	0.23
24.77	0.58	-0.27	-0.73	0.25
24.78	0.57	-0.27	-0.73	0.27
24.8	0.56	-0.27	-0.73	0.28
24.82	0.56	-0.26	-0.73	0.29
24.83	0.56	-0.26	-0.73	0.29
24.85	0.56	-0.25	-0.74	0.28
24.87	0.55	-0.25	-0.75	0.27
24.88	0.55	-0.24	-0.76	0.25
24.9	0.54	-0.23	-0.77	0.23
24.92	0.53	-0.23	-0.79	0.20
24.93	0.52	-0.22	-0.81	0.18
24.95	0.50	-0.21	-0.83	0.16
24.97	0.48	-0.20	-0.84	0.14
24.98	0.46	-0.19	-0.86	0.12
25	0.44	-0.18	-0.87	0.11
25.02	0.42	-0.17	-0.89	0.09

25.03	0.39	-0.17	-0.90	0.08
25.05	0.36	-0.16	-0.92	0.06
25.07	0.33	-0.16	-0.93	0.05
25.08	0.29	-0.17	-0.94	0.03
25.1	0.24	-0.18	-0.95	0.01
25.12	0.19	-0.20	-0.96	-0.00
25.13	0.14	-0.22	-0.97	-0.02
25.15	0.09	-0.24	-0.97	-0.03
25.17	0.05	-0.26	-0.96	-0.04
25.18	0.00	-0.29	-0.96	-0.05
25.2	-0.03	-0.30	-0.95	-0.06
25.22	-0.07	-0.32	-0.94	-0.06
25.23	-0.10	-0.33	-0.94	-0.07
25.25	-0.12	-0.34	-0.93	-0.07
25.27	-0.15	-0.35	-0.92	-0.08
25.28	-0.17	-0.36	-0.91	-0.09
25.3	-0.19	-0.36	-0.91	-0.10
25.32	-0.21	-0.36	-0.90	-0.10
25.33	-0.22	-0.37	-0.90	-0.11
25.35	-0.23	-0.37	-0.89	-0.12
25.37	-0.24	-0.37	-0.89	-0.12
25.38	-0.24	-0.37	-0.89	-0.13
25.4	-0.24	-0.37	-0.89	-0.13
25.42	-0.24	-0.37	-0.89	-0.14
25.43	-0.24	-0.36	-0.89	-0.14
25.45	-0.24	-0.36	-0.89	-0.15
25.47	-0.24	-0.36	-0.89	-0.15
25.48	-0.24	-0.36	-0.89	-0.15
25.5	-0.23	-0.36	-0.89	-0.15
25.52	-0.23	-0.36	-0.89	-0.14
25.53	-0.22	-0.36	-0.90	-0.14
25.55	-0.21	-0.36	-0.90	-0.13
25.57	-0.19	-0.36	-0.90	-0.12
25.58	-0.18	-0.36	-0.91	-0.11
25.6	-0.16	-0.36	-0.91	-0.10
25.62	-0.14	-0.35	-0.92	-0.09
25.63	-0.12	-0.35	-0.93	-0.08
25.65	-0.10	-0.34	-0.93	-0.06
25.67	-0.09	-0.33	-0.94	-0.05
25.68	-0.08	-0.32	-0.94	-0.03
25.7	-0.07	-0.31	-0.95	-0.01
25.72	-0.06	-0.31	-0.95	0.02
25.73	-0.06	-0.30	-0.95	0.05
25.75	-0.05	-0.29	-0.95	0.09
25.77	-0.04	-0.29	-0.95	0.14
25.78	-0.03	-0.27	-0.94	0.19
25.8	-0.02	-0.26	-0.93	0.24

25.82	-0.01	-0.24	-0.93	0.29
25.83	0.01	-0.22	-0.91	0.35
25.85	0.03	-0.19	-0.90	0.40
25.87	0.04	-0.16	-0.88	0.45
25.88	0.06	-0.13	-0.85	0.51
25.9	0.08	-0.11	-0.82	0.56
25.92	0.09	-0.09	-0.79	0.60
25.93	0.11	-0.08	-0.75	0.65
25.95	0.11	-0.07	-0.72	0.69
25.97	0.12	-0.05	-0.68	0.72
25.98	0.12	-0.04	-0.64	0.76
26	0.12	-0.03	-0.60	0.79
26.02	0.12	-0.00	-0.55	0.83
26.03	0.12	0.02	-0.50	0.86
26.05	0.11	0.06	-0.44	0.89
26.07	0.10	0.09	-0.38	0.91
26.08	0.09	0.13	-0.31	0.94
26.1	0.08	0.16	-0.25	0.95
26.12	0.08	0.18	-0.18	0.96
26.13	0.09	0.20	-0.11	0.97
26.15	0.10	0.21	-0.05	0.97
26.17	0.11	0.22	-0.00	0.97
26.18	0.13	0.23	0.04	0.96
26.2	0.14	0.25	0.08	0.96
26.22	0.14	0.27	0.11	0.95
26.23	0.14	0.29	0.13	0.94
26.25	0.13	0.31	0.14	0.93
26.27	0.11	0.33	0.14	0.93
26.28	0.09	0.34	0.14	0.93
26.3	0.06	0.34	0.13	0.93
26.32	0.02	0.34	0.12	0.93
26.33	-0.01	0.33	0.11	0.94
26.35	-0.04	0.33	0.10	0.94
26.37	-0.07	0.32	0.10	0.94
26.38	-0.09	0.32	0.10	0.94
26.4	-0.10	0.33	0.11	0.93
26.42	-0.11	0.35	0.11	0.92
26.43	-0.12	0.37	0.12	0.92
26.45	-0.13	0.38	0.12	0.91
26.47	-0.13	0.40	0.10	0.90
26.48	-0.14	0.40	0.08	0.90
26.5	-0.15	0.40	0.05	0.90
26.52	-0.17	0.39	0.01	0.91
26.53	-0.18	0.37	-0.04	0.91
26.55	-0.20	0.35	-0.08	0.91
26.57	-0.21	0.34	-0.12	0.91
26.58	-0.23	0.33	-0.15	0.90

26.6	-0.24	0.33	-0.17	0.89
26.62	-0.26	0.34	-0.19	0.89
26.63	-0.27	0.35	-0.19	0.88
26.65	-0.28	0.35	-0.19	0.87
26.67	-0.29	0.36	-0.19	0.87
26.68	-0.29	0.35	-0.18	0.87
26.7	-0.29	0.35	-0.16	0.88
26.72	-0.29	0.34	-0.14	0.88
26.73	-0.29	0.33	-0.13	0.89
26.75	-0.30	0.34	-0.11	0.89
26.77	-0.31	0.35	-0.09	0.88
26.78	-0.33	0.36	-0.08	0.87
26.8	-0.35	0.38	-0.07	0.85
26.82	-0.37	0.40	-0.06	0.84
26.83	-0.39	0.40	-0.07	0.83
26.85	-0.39	0.38	-0.08	0.83
26.87	-0.39	0.35	-0.10	0.84
26.88	-0.38	0.30	-0.13	0.86
26.9	-0.37	0.26	-0.17	0.88
26.92	-0.35	0.21	-0.20	0.89
26.93	-0.33	0.18	-0.24	0.90
26.95	-0.31	0.16	-0.26	0.90
26.97	-0.30	0.15	-0.28	0.90
26.98	-0.30	0.16	-0.29	0.89
27	-0.31	0.17	-0.29	0.89
